# Non-lethal imaging and modeling approaches for estimating dry mass in aquatic larvae

**DOI:** 10.1371/journal.pone.0345767

**Published:** 2026-04-17

**Authors:** Daniela Granados Frias, Najva Akbari, Lauren A. O’Connell, Bryan H. Juarez

**Affiliations:** 1 Department of Biology, Stanford University, Stanford, California, United States of America; 2 Earth System Science Department, Stanford University, Stanford, California, United States of America; Laboratoire de Biologie du Développement de Villefranche-sur-Mer, FRANCE

## Abstract

Body mass is crucial for scaling and comparing physiological rates. Specifically, dry body mass is important in determining an organism’s metabolic rate since it excludes metabolically inactive water weight. While obtaining repeated measurements of body mass throughout an individual’s lifetime is trivial, we can obtain only a single estimate of dry body mass since classical methods require end-point euthanasia. Here, we present imaging and modeling techniques for estimating individual dry body mass in African clawed frog (*Xenopus laevis*) tadpoles, which allow repeated sampling of the same individuals. We applied allometric principles and tested whether external anatomy would yield reliable estimates of dry body mass. Specifically, we describe a procedure to embed tadpoles in agarose media for obtaining morphological data in 3-D and then we evaluate dry mass predictions among nine cross-validated maximum likelihood and machine learning models. The best performing and most flexible model was an allometric model that used estimates of body volume to predict dry body mass. However, other models based on wet body mass or fewer input variables may also be logistically tractable. This research develops a foundation for continued research on the biological importance of dry body mass, particularly in the context of development and physiological ecology.

## Introduction

Body mass is a key feature used to understand processes in physiology, ecology, and evolution. For example, body size is central in studies of organismal growth and development [1–3], physiology [4–6], movement and distribution [7–9], ecogeographical gradients [10–12], and even survival [13]. In the fields of physiological ecology or evolutionary ecology [14], body mass is necessary for understanding the mechanisms that drive the empirical pattern of metabolic scaling [5,15,16] and comparing physiological rates among cells, tissues, individuals, or species of different sizes [5,17–22]. Overall, body mass is an important trait in biology that is correlated with physiological rates or is used to standardize rates to account for body size.

Researchers have quantified mass in many ways when performing comparisons of physiological rates. Commonly used body mass indices include wet mass [23–29], dry mass [23,25,27,29–31], lean mass [23,24,32], dry lean mass [23], or combinations thereof [23,25–27,29,33]. The choice of mass index can depend on the logistics of data collection in the field or lab, affordability, or model assumptions related to correlations among the different mass indices and selected physiological rates. For example, dry body mass is used as an indicator of metabolic rate since it factors out the non-metabolizing mass of water in the body. Previous work required regression-based linear transformations among body mass indices, such as among dry and wet mass [34,35], but these types of methods have not been developed for many taxa. Additionally, integrating physiological rates with individual (offspring) or parental behaviors is crucial for understanding the mechanisms driving rate variation. For example, the ratio of neonate dry mass to egg dry mass (parentotrophy index) is used as a measure of parental resource provisioning which impacts individual growth rates [36,37]. Furthermore, many of the latter indices are typically only obtainable once following euthanasia. While currently available tools allow correlations between wet body mass and physiological rates through time [38–40], the same does not apply to other mass indices. The latter is a barrier in physiological research since it limits our ability to investigate relationships among body mass indices, physiological rates through time (across the seasons, development, or throughout the lifespan), and any important abiotic or biotic covariates (like behavior). Instead, researchers depend on relating repeated measurements of physiological rates through time with a single estimate of body mass obtained at or near the experimental endpoint [23,41–44] or by comparing population means between discretely modeled time points like seasons [25,29,45,46]. What is needed is a method to obtain repeated estimates of dry body mass on the same individuals without the need for euthanasia.

Body mass is the net result of an animal’s metabolic needs and energy consumption, or in other words, body mass is the product of growth [5,15,47]. Since growth may also be expressed not by an organism’s mass, but by an organism’s volume, we can express growth and body mass in terms of changes in volume [48–50]. One approach for estimating body mass through morphology is by applying allometric scaling principles [51–53]. Specifically, measurements of lengths, areas, or volumes on the body can be used to estimate the body volume, which is proportional to body mass. To obtain reliable estimates of dry body mass, one can empirically estimate a linear transformation between body lengths, areas, or volumes and body mass after drying. Few researchers have attempted to develop surrogates for dry or lean body mass in vertebrates. Most research in this area comes from plant biology where the dry body mass of leaves or stems have been estimated through allometric scaling approaches based on linear measurements or surface areas [54,55]. In terrestrial and aquatic invertebrate animals, lengths, wet body masses, and other body measurements are correlated to dry body masses in live, dried, or preserved specimens [56–59]. In vertebrates, related studies have focused on quantifying water loss following preservation as a function of initial wet body mass [60–63]. While wet body mass is an indicator of water loss during preservation, we still lack a general understanding of how morphology, animal or tissue volumes, and dry body mass are linked in vertebrate animals. Some exceptions include studies that have correlated linear measurements in animals with whole-animal or tissue volumes [64–66]. Overall, morphology-based empirical approaches are promising in developing predictive models of dry mass or dehydrating processes.

Amphibian larvae are a great system for learning how anatomy varies with dry body mass. Specifically, anuran (frog and toad) tadpoles are easy to work with and are commercially available. For example, clawed frog (*Xenopus*) larvae are easily obtainable, the husbandry is simple [67,68], and the small size of tadpoles makes them amenable to studies of functional morphology and metabolic rates alike. The larvae of many frogs or toads, including *Xenopus*, are also partly translucent, allowing for easy measurement of a variety of traits [69,70]. As predicted from form-function relationships and the physiology of surface area-to-body ratios, small-bodied (cm-scale) tadpoles will dry faster than larger (m-scale) vertebrates [38,71,72]. Additionally, the permeable skin of tadpoles makes them particularly susceptible to dehydration [73]. Looking forward, correlating dry body masses and physiological rates in amphibian larvae will give insights into the mechanisms driving rate variation, particularly in the context of the environment, growth, and behaviors, making them a target for applied research [73–79].

The purpose of this study is to design new tools for obtaining repeated measures of dry body mass from the same individuals without euthanasia. We accomplish this by measuring all predictor and target variables of interest (including dry body mass) on a group of tadpoles whose body size (and shape) variation is representative of morphological changes throughout an individual tadpole’s life. We then use those data to create and validate regression models that can be used in future studies to estimate dry body mass repeatedly throughout individual lifetimes without euthanasia. Additionally, we seek to estimate the relationship between wet and dry body mass which is important in transforming between different body mass indices. Following allometric principles, we hypothesize that morphological lengths, areas, and volumes are strong indicators of dry body mass. We predict that body volume is a significant predictor of dry body mass and that body volume is the best predictor of dry body mass relative to surface areas and lengths, possibly due to the limitations of using predictors of lower dimensions when predicting dry body mass. Here, we demonstrate how we may reliably estimate dry body mass using several alternative models and these models are suitable for obtaining dry body mass through ontogeny. This study opens the doors for understanding how abiotic and biotic factors impact physiology, growth, and development through an organism’s life, independently of non-metabolizing water content in the body.

## Methods and materials

### Animals and housing

This study was completed in strict accordance with the recommendations in the Guide for the Care and Use of Laboratory Animals of the National Institutes of Health. The protocol was approved by Stanford University under the Administrative Panel on Laboratory Animal Care (APLAC, Protocol #33097). All efforts were made to minimize suffering. *Xenopus laevis* tadpoles of Gosner stages 44–54 (N = 61; Xenopus 1, Corp (Dexter, Michigan, USA)) were placed into aquaria (18.09 cm L x 11.13 cm W x 13.34 cm H). The tank included 0.1X Marc’s modified Ringer’s solution (MMR) to avoid osmotic stress [80], shelter made of PVC pipe, and an air pump with a bubbling stone to aerate the water tank. Tanks were maintained at 25℃ and 50% water changes (0.1X MMR) were performed every 2 days and as needed. Tadpoles were fed crushed tadpole food pellets (Josh’s Frogs, Owosso, Michigan, USA) three times per week. Animals were isolated for 24 hours without food prior to imaging and weighing to allow time for defecation and to avoid confounding our estimates of wet and dry body mass with food mass.

### Imaging and data collection

Tadpole embedding, imaging, and measurement methods are available in the S1 Protocol and at protocols.io: dx.doi.org/10.17504/protocols.io.rm7vzk1wrvx1/v1 [81]. Briefly, each tadpole was anesthetized in a container with 10 mL of 0.03% buffered MS-222 for roughly 1 min [82]. We lightly brushed the tadpole’s body with a paint brush to confirm successful anesthesia by observing complete lack of movement. Next, we filled a chambered coverglass (5 cm L x 2 cm W x 1 cm H; Thermo Scientific, Waltham, Massachusetts, USA) halfway with fluid low-melting point 1.5% agarose. This agarose melts at 42℃, however, in practice, the agarose solution cooled rather quickly after we removed it from heat. We placed the tadpole into this chamber and positioned its body parallel to the length of the coverglass, but closer to one corner of the coverglass to improve the quality of the photographs and measurements. Placing the tadpole medially and parallel to the length of the coverglass resulted in poor quality photographs and decreased measurement accuracy during preliminary trials. After confirming optimal body positioning, we added 2–3 drops of MS-222 with a plastic transfer pipette to ensure anesthesia throughout the entire imaging process and then filled the chambered coverglass containing the tadpole with melted agarose. *Ad hoc* addition of MS-222 was necessary (e.g., if the tadpole twitched) to guarantee the efficacy of the procedure for all tadpoles. Each tadpole was embedded in the agarose for no more than five minutes.

Embedding tadpoles in the chambered coverglass allowed imaging of the whole tadpole from all three dimensions (dorsal, lateral, and frontal). Images were taken using a Leica Si9 microscope at a magnification of 0.6x and 1x. The pictures were taken using the default settings in the LAS EZ v. 3.4.0 (Leica Microsystems, Wetzlar, Germany). We included 1 mm grid paper placed in the same horizontal plane as the tadpole in every image to convert pixels to real distances. After imaging, we removed the tadpoles from the agarose media by gently breaking apart the agarose using a paint brush and then placed them into conditioned (isotonic) DI water to remove any agarose clinging onto the body. Once clean, we transferred the tadpoles into a petri dish to remove excess water and obtained the wet mass on an analytical scale (Model PMF523/E, Fisher Scientific, Pittsburgh, United States). This scale was used for all mass measurements.

We euthanized the tadpoles by applying 20% benzocaine gel to the body and confirming the animal was unresponsive prior to flash freezing in liquid nitrogen. Briefly, a small petri dish was placed in a container filled with liquid nitrogen until equilibrium was reached. The petri dish was then moved to an insulated container and filled with liquid nitrogen. Then, we placed the tadpole into the liquid nitrogen and covered the petri dish with a lid to guarantee euthanasia. The insulated container was filled with liquid nitrogen until the petri dish was halfway immersed and then covered with a lid to minimize heat transfer. We weighed the tadpoles on a pre-weighed glass slide 15 minutes after flash freezing. Next, we placed the glass slide with the tadpole in a separate petri dish and moved it to a drying oven set at 37℃ and left to dry for a minimum of 72 hours. A preliminary study showed tadpoles completely dried after only 48 hours and the mass did not decrease after 1 week. The dry mass was obtained by subtracting the weight of the glass slide from the final weight of the dried tadpole on the glass slide.

**Fig 1:**
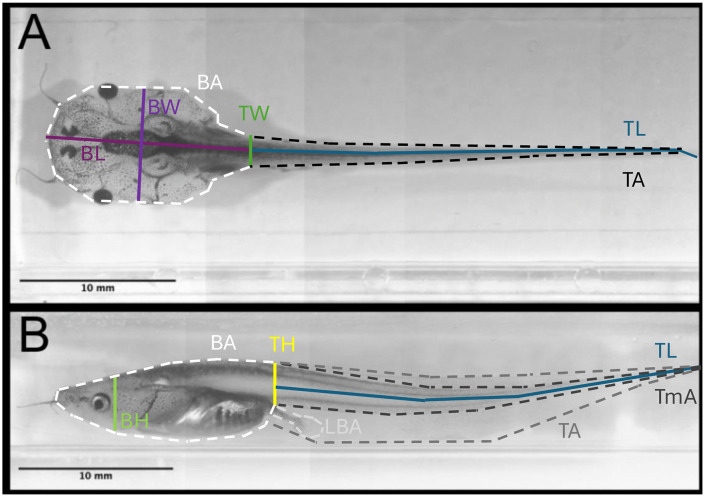
Tadpoles embedded in agarose media and anatomical traits measured. (A) Dorsal measurements obtained in this study (see Table 1). (B) Lateral measurements obtained in this study (see Table 1). BL is body length, BW is body width, BL is body height, BA is body area, TL is tail length, TW is tail width, TH is tail height, TA is tail area, TmA is tail muscle area, and LBA is limb bud area. Images C, D are composite images obtained by stitching individual images (see Methods and Materials).

We obtained a total of 27 measures from each tadpole image using Fiji v. 1.54f [83]. Fifteen were linear measurements (Table 1, Fig 1, S1 Fig) including (1) the dorsal length, width, and area for the body and the tail, (2) the lateral body height, tail length and height, and the body, tail, tail muscle, and limb bud areas, and (3) the frontal body width and area. We also obtained 12 additional (composite) variables, including tail muscle area, tail volume, body volume estimated in different ways (Table 1), and the wet and dry body masses of each tadpole.

**Table 1:** Initial input variables used in all models. M is model (see Data analysis in Methods). View is the 3-dimensional origin of each trait. NA is not available, L is lateral, D is dorsal, F is frontal, and Composite is traits obtained from two views. T is tail, Tm is tail muscle, B is body, A is area, L is length, W is width, and H is height. 1 and 2 indicate traits included in the initial and final fits for each model (M1–8), respectively.

View	Units	Trait	Description	M1	M2	M3	M4	M5	M6–8
NA	g	wet mass		1, 2					1, 2
Dorsal	mm	body length	snout-vent		1				1, 2
	mm	body width	postorbital width		1				1, 2
	mm	tail length	vent to end of tail		1				1, 2
	mm	tail width	width at vent		1, 2				1, 2
	mm^2^	body area				1			1, 2
	mm^2^	tail area				1, 2			1, 2
Lateral	mm	body height	postorbital height		1, 2				1, 2
	mm	tail length			1, 2				1, 2
	mm	tail height	height at vent		1, 2				1, 2
	mm^2^	body area				1			1, 2
	mm^2^	tail area				1			1, 2
	mm^2^	tail muscle area				1			1, 2
	mm^2^	fin area	LTA-LTmA			1, 2			1, 2
	mm^2^	limb bud area			1, 2	1, 2			1, 2
Frontal	mm	body width	eye to eye		1, 2				1, 2
	mm^2^	body area				1, 2			1, 2
Composite	mm^3^	body volume 1	LBA*DBW				1		1, 2
	mm^3^	body volume 2	LBA*FBW				1		1, 2
	mm^3^	body volume 3	LBH*DBA				1, 2		1, 2
	mm^3^	body volume 4	DBL*FBA				1		1, 2
	mm^3^	tail volume 1	LTA*DTW				1		1, 2
	mm^3^	tail volume 2	LTH*DTA				1		1, 2
	mm^3^	tail muscle volume	LTmA*DTW				1, 2		1, 2
	mm^3^	body volume 5	LBH*DBL*DBW					1, 2	1, 2
	mm^3^	tail volume 3	LTH*DTL*DTW					1, 2	1, 2

### Data analysis

We compared the performance of eight models to test our predictions that morphology, namely body volumes, are reliable estimators of dry body mass. The eight models included those of wet body mass (model 1), length measurements (model 2), surface areas (model 3), volumes estimated in two different ways (models 4–5), and all data (models 6–8). The first five models were ordinary least squares (OLS) regression models optimized using least squares in a maximum likelihood framework. Models 6–8 were machine learning models including one random forest, two adaptive lasso, and one neural network. Briefly, random forest models are optimized by averaging across many decision trees, which each use a subset of the data and input variables for prediction [84]. The first lasso model sparsely optimized the mean square error while the second gave the sparsest model within one standard error of the minimum loss, e.g., the mean square error [85]. We included machine learning models since each has beneficial properties that may outperform likelihood-based models. For example, random forest models allow for the modeling of potential non-linearities, the adaptive lasso optimizes prediction ability while reducing the number of necessary input variables and exhibits the oracle property [85], neural networks can model all possible interactions in a dataset, and all can perform automatic feature selection (taking multicollinearity into account). We used variance inflation factors (VIF) and a cutoff of VIF = 10 to remove collinear terms from each likelihood model [86]. For example, we obtained estimates of body width from both dorsal and lateral views but either may serve as an optimal predictor of dry mass. We show the initial and final variables for each model in Table 1. We natural log-transformed all data except limb bud area which we square root transformed since some tadpoles did not have limb buds. All reported statistical tests used an alpha of 0.05. Finally, we evaluated prediction ability in each model after confirming that each model exhibited appropriate model diagnostics.

We used repeated K-fold cross-validation to evaluate model performance as determined primarily by the mean square error and its standard deviation. We also measured the mean absolute error, r^2^, and their standard deviations. Briefly, repeated K-fold cross validation is an extension of cross-validation where cross-validation is repeated many times using K data (training/validation) splits to obtain average performance metrics across repeats [87,88]. Additionally, cross-validation is a standard two-step procedure used to determine the generalizability of predictive models. The first step is to fit the model to a subset of the data (the training set) to obtain parameter estimates. The second step assesses the generalizability of the model by using the parameter estimates to obtain model performance metrics on unseen data (the validation set). Specifically, we used 200 repeats of 5-fold cross-validation to guarantee each randomly sampled validation set contained at least 10 samples (N = 61 samples / 5 folds = 12.2 samples per validation set) and to estimate error for each prediction metric using at least 1,000 (200 x 5) validation sets. We implemented this procedure for models 1–6 using the caret package v. 6.0-94 in R v. 4.4.1 [87,89]. To obtain comparable metrics for the adaptive lasso, we back-calculated the population mean and standard deviations for all performance metrics across 200 samples of (non-repeated) 5-fold cross-validation using the R package glmnet 4.1-8 [90,91]. We were not able to obtain estimates for the *r*^*2*^ of the adaptive lasso since this is not currently implemented in glmnet 4.1-8. Next, we describe how we fitted the neural network model and obtained its performance metrics.

We implemented a feed-forward neural network using keras3 v. 1.2.0 and tensorflow v. 2.16.0 in R [92,93]. In general, a neural network is a network-based machine learning technique that explores network parameters with the goal of minimizing a loss (error) function. Neural networks include two hyperparameters that help define the network and its complexity; the neural layers define the depth of the network while the neurons per layer (neural density) control the number of attempted linear transformations per layer. Here, the linear transformations refer to the weight and bias parameters, similar to a slope and intercept. While data are linearly transformed many times within the neural network, a single parameterized neural network produces a single set of predictions (like regression). Fitting a neural network involves iteratively finding the weights and biases at each neuron that minimize the loss function. This is accomplished by using a training set to sample network parameters (the weights and biases at each neuron), a validation set to tune hyperparameters (where model performance is evaluated after each iteration), and the testing set to give an unbiased estimate of out-of-sample model performance (generalizability) using unseen data.

We used a cross-validation approach for fitting and evaluating neural networks across a range of chosen hyperparameters (neural layers and number of neurons per layer). Prior to fitting the model, we split the data into 60-20-20% training, validation, and testing sets to guarantee the model validation and testing steps were performed on at least 12 samples and the model was trained using at least 30 (N = 36) samples. We selected hyperparameters by determining the hyperparameter combination that resulted in the lowest validation error (mean square error). Here, we define validation error as the median of 3 replicate model fits for each hyperparameter combination. This is necessary to reduce sampling error since fitting neural networks includes a stochastic component that affects model performance. The hyperparameters we varied included the number of neural layers from 0 to 25 (by one) and the number of neurons per layer from 200 to 2,500 (by 100), each using a learning rate of 0.001 across 200 epochs. We selected this arbitrarily broad range after determining the standard suggestions of 1–3 layers and neurons per layer of 0.5X, 1X, and 2X the sample size did not yield usable results, but we still included these in the hyperparameter search [94–96]. We also applied a stop rule to end sampling if the validation error (mean square error) did not decrease after 50 epochs to protect against oversampling local minima across the search space and decrease computation time. We obtained the final out-of-sample performance metrics (mean square error, mean absolute error, and r^2^) using the testing set. All sampled networks contained a normalizing layer (to improve model quality and reduce computation time), used Rectified Linear Unit (ReLU) activation for signal propagation (to avoid vanishing gradients), and used an Adam optimizer for automatic tuning of the learning rate [97,98].

## Results

**Fig 2:**
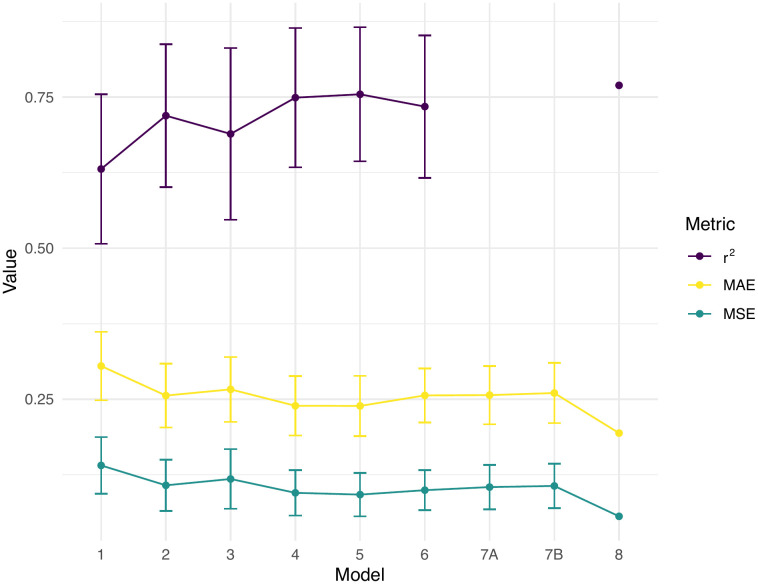
Plot of performance metrics for surveyed models. Models are as in Table 1 and the main text. Models 1–6 are likelihood models and models 6–8 are machine learning models. MAE is the mean absolute error and MSE is the mean square error. Error bars indicate ∓ 1 standard deviation of the mean. r^2^ is not implemented for model 7. Model 8 lacks error bars because metrics are based on a single testing set of N = 12. The theoretical best model has a high r^2^ and low errors (MAE or MSE).

Overall, we found that most models exhibited similar prediction ability with notable differences in the AICc of likelihood models and in the validation or testing mean square error of each model (Fig 2; Table 2). We provide the predictive equations and relevant statistics for each model in the S1 Text. Tadpoles were 22.48–46.04 mm (mean = 33.67, std. dev. = 5.50) in total length, exhibited wet body masses of 4.745–4.982 g (mean = 4.834, std. dev. = 0.062), and had dry body masses of 0.002–0.017 (mean = 0.008, std. dev. = 0.004). We found all tadpoles dried completely within 48 hours. We describe each model below.

**Table 2:** Performance metrics for surveyed models. Rank is the model rank based on MSE and shown only for validation metrics obtained using repeated K-fold cross validation. # Var is the number of final predictor variables. AICc is the Akaike Information Criterion corrected for small sample size for likelihood models. MSE is the mean square error. MAE is the mean absolute error. r^2^ is the coefficient of determination. SD is the standard deviation. *r*^*2*^ is not implemented for model 7. Model 8 lacks standard deviation estimates because metrics are based on a single testing set of N = 12. The theoretical best model has a high *r*^*2*^, low errors (MAE or MSE), and a low AICc.

Model (Rank)	# Var	AICc	MSE	MAE	r^2^	MSE SD	MAE SD	r^2^ SD
1 (8)	1	56.4515	0.1405	0.3050	0.6310	0.0469	0.0566	0.1237
2 (6)	6	38.1121	0.1076	0.2561	0.7192	0.0424	0.0530	0.1182
3 (7)	5	46.4331	0.1181	0.2663	0.6891	0.0494	0.0536	0.1420
4 (2)	2	33.1050	0.0952	0.2392	0.7491	0.0376	0.0491	0.1152
5 (1)	2	30.9366	0.0922	0.2390	0.7546	0.0358	0.0498	0.1110
6 (3)	26	–	0.0996	0.2564	0.7343	0.0331	0.0447	0.1179
7A (4)	6	–	0.1047	0.2568	NA	0.0368	0.0482	NA
7B (5)	5	–	0.1066	0.2603	NA	0.0368	0.0498	NA
8	26	–	0.0563	0.1940	0.7694	NA	NA	NA

**Fig 3:**
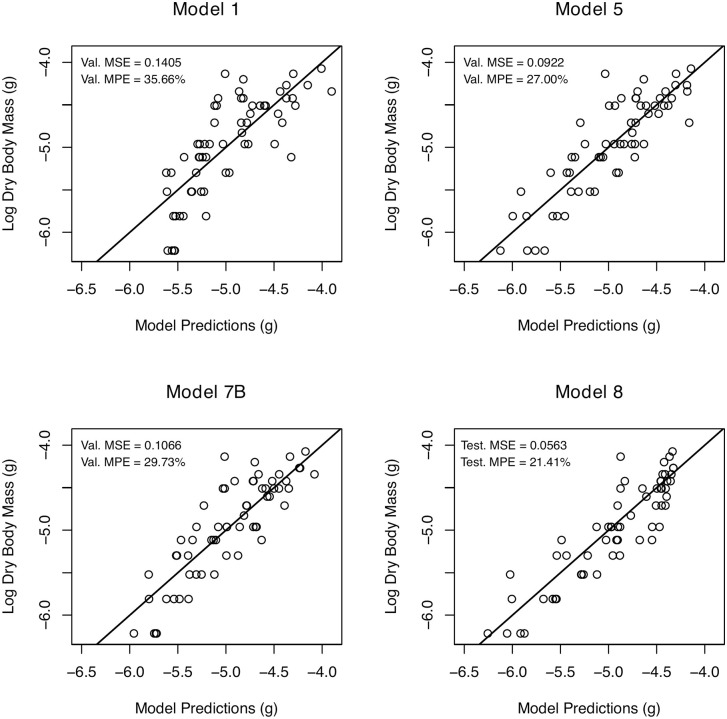
Actual versus predicted plots for select models. These models compare measured and predicted values of dry body mass and include the wet body mass model (1), the best likelihood model (5), an adaptive lasso model (7B), and a neural network model (8). Each point is an individual tadpole. Val. MSE is the validation mean square error and Val. MPE is the validation mean percentage error. Test. MSE and Test. MPE are the mean square error and mean percentage error for the testing set of model 8. The solid line is the 1:1 line. The theoretical best model has low errors (MSE, MPE).

The likelihood models (1–5) exhibited statistically similar validation metrics (Fig 2), with model 5 standing out as the single best likelihood model according to AICc (Table 2). The wet body mass model (1) yielded the predictions with the lowest accuracy (highest validation error). The mean absolute error of the natural log of wet body mass was 0.3050 and yields a mean percentage error of 35.66% = (*e*^0.3050^ −1) × 100% (Fig 3). Despite this, wet body mass is a significant predictor of dry body mass in the measured tadpoles (Table A in S1 Text; *F* = 89.342, *p* < 0.001). The lengths model (2) ranked 6th in predictive ability and exhibited a mean percentage error of 29.19%. The dorsal tail width, lateral body height, and lateral tail height were all significant predictors of dry body mass (Table B in S1 Text; *F* = 13.564–122.775, *p* ≦ 0.001). The surface area model (3) ranked 7th in predictive ability with a mean percentage error of 30.51%. Model 3 had two significant predictors including lateral fin area (Table C in S1 Text; *F* = 8.030, *p* = 0.006) and dorsal tail area (*F* = 118.955, *p* < 0.001). The volumes model (4), whose volumes were estimated from areas and lengths, was the 2nd best model (Table 2). Model 4 exhibited a mean percentage error of 27.02%. Its significant predictors included body volume, as estimated from the product of lateral body height and dorsal body area (Table D in S1 Text; *F* = 155.779, *p* < 0.001), and tail muscle volume which we obtained as the product of lateral tail muscle area and dorsal tail width (Table D in S1 Text; *F* = 8.237, *p* = 0.006). The last likelihood model (5), ranked highest among the comparable likelihood and machine learning models and had a mean percentage error of 27.00% (Fig 3). Its predictors included body volume (Table E in S1 Text; *F* = 161.939, *p* < 0.001) estimated as the product of dorsal body length, dorsal body width, and lateral body height, and tail volume (Table E in S1 Text; *F* = 10.111, *p* = 0.002) obtained as the product of dorsal tail length, dorsal tail width, and lateral tail height.

**Fig 4:**
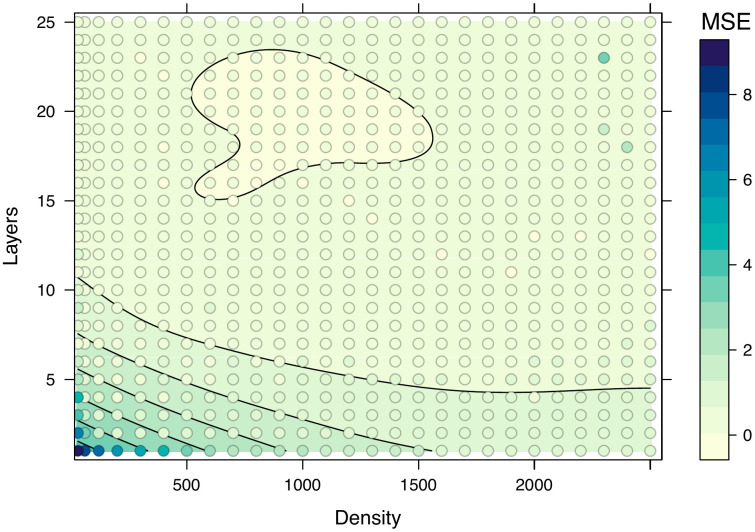
Level plot of hyperparameter search results for neural network (model 8). Layers is the number of neural layers, density is the number of neurons per layer, and MSE is the (validation) mean square error. Contour lines generally correspond to the discrete differences in MSE shown in the legend. The network with the lowest validation error had 11 layers and 1900 neurons.

As a whole, the machine learning models provide flexible and similar alternatives to likelihood models (Fig 2; Table 2). After automatic tuning of the number of variables per split (*m* = 2), the random forest model (6) yielded a mean percentage error of 29.23%. Estimates of variable importance, or the average decrease in mean square error after splitting on each variable, are in Table F in S1 Text. Briefly, lateral body area was the most important variable (importance = 1.231) and the lateral limb bud area was the least important (importance = 0.276). The adaptive lasso model (7) yielded a variety of sparse solutions, where some regression parameters are set to 0, depending on the regularization parameter *λ*. Two solutions include the optimal sparse prediction model (minimum *λ*; model 7A) and the sparsest model within one standard error of the minimum *λ* (model 7B). The mean percentage errors for model 7A and B were 29.28% and 29.73% (Fig 3), respectively. Table G in S1 Text shows the selected variables and coefficient estimates for models 7A and B. Model 7A selected wet body mass, dorsal body length, dorsal tail width, lateral body height, lateral tail height, and frontal body width as predictors. Model 7B selected the same variable set as 7A but excluded dorsal tail width. Finally, the hyperparameter search for the neural network model (8) showed 11 layers and 1900 neurons per layer gave the lowest validation error among surveyed network structures (Fig 4). The hyperparameter search results are found in the Supplementary Material (Table H in S1 Text). We estimated network parameters using the latter hyperparameters and found a mean square error of 0.0563 (Fig 2; Table 2) and mean percentage error of 21.41% for the testing set (Fig 3). However, these performance metrics are not directly comparable with the other models (see Discussion).

## Discussion

Here we present new methods using body lengths, areas, and volumes to estimate the dry body mass of *Xenopus laevis* tadpoles more than once through their lifetime and without lethality. Our approach is a general method that should be validated in other vertebrates. Researchers wanting to apply these methods should take photographs, measure tadpoles, and apply the regression equations of their choice (models 1–8) to estimate dry body mass continuously through the individual’s life. These results align with studies that predict dry body mass using linear measurements in plants [55] and insects [56–59]. Notably, those insect studies found body length and head dimensions to be important predictors of dry body mass and this is similar to what we found in tadpoles. Our finding that surface areas can predict dry body mass is similar to past work in plants [54]. While we predicted body volume would be a good predictor of body mass, we did not predict models using surface areas would perform worse than models using lengths. This is possibly due to surface areas involving neither direct estimation of volumes nor individual and independent predictors (lengths), leading to a relatively inaccurate allometric model connecting spatial dimensions with dry body mass. As in insects [59], we also found wet body mass is correlated to dry body mass, although we identified better morphological predictors. Overall, these interpretations highlight the importance of developing similar predictive models in other animal species. Below, we discuss our findings in greater detail, offer some best practices for users of these methods, and discuss some future directions of this research, particularly within the field of physiological ecology.

### Comparison of models for estimating dry mass

We found several viable modeling alternatives for predicting dry mass. Most models exhibited similar variances with small differences in mean validation metrics (Table 2). While using volumes in model 5 improved our estimates compared to all other likelihood models, some of these models may still be of practical use to many readers. For example, although using wet body mass (model 1) underperformed relative to body volumes (model 5), wet body mass was still a significant predictor of dry body mass. Measuring wet body mass is relatively simple and this simple approach may be suitable for many studies despite a difference in the mean percentage error of 8.66% relative to model 5. Models 2–4 provide intermediate and viable alternatives for obtaining estimates of dry body mass and only require users to obtain four to six independent measurements. To obtain accurate estimates of dry body mass using likelihood models, we recommend using model 5, which includes six measurements: the lateral body height, dorsal body length, dorsal body width, lateral tail height, dorsal tail length, and the dorsal tail width.

We used machine learning models to provide additional alternatives, including the adaptive lasso and neural network models. The adaptive lasso models (7) should be used with caution since they reach a high level of prediction accuracy at the cost of outputting slightly biased estimates. This makes the lasso models appropriate for determining the magnitude of group effects and covariances, but not for accurately estimating or interpreting individual dry body mass. The neural network model (8) exhibited good quantitative performance, but it may not be ideal for many situations and comes with many drawbacks. First, we do not have an ideal way of performing model comparisons among neural networks and other model classes because the validation metrics are based on a single testing set and not cross-validation. While methods such as stratified K-fold cross validation [99] are available for neural networks, this would still require performing a computationally expensive hyperparameter search for each testing set. An alternative that would enable direct comparisons to other models would be to define one or many testing sets for all models at the cost of reducing the sample size of the testing and validation sets for other models. A second drawback to using model 8 for predicting dry mass is that it exhibits the undesirable behavior of predicting the same value for some of the largest tadpoles and this can be seen as a vertical ridge in Fig 3, which can impact downstream applications and interpretability. Third, the neural network model comes with the significant drawback of requiring all of the variables in Table 1 for predictions. As discussed, machine learning models can provide many advantages, given that we understand the properties of the implemented model.

### Future modeling improvements

There are many practical alternatives for data collection and potential future improvements to the presented models. For example, in this study we used agarose media to embed tadpoles in chambered coverglasses but a potentially viable alternative is taking morphological measurements using optical micrometers and measuring tadpoles in chilled water to minimize movement but allow respiration and minimize stress. If users wish to implement end-point euthanasia, measurements may also be taken using photogrammetry [100,101], which may allow for more accurate estimation of body volume. Future improvements to our models include increasing the sample sizes to allow for larger training, validation, and testing sets. Furthermore, robust linear models [102,103] might offer solutions for potential heteroscedasticity issues with predictions made from model 8. We also encourage empirically validating predictions or even comparing predictions among models 1–8 to determine whether the data qualities suit the needs of downstream analyses. In downstream analyses where dry body mass is used as an independent variable, users may set weights or use measurement error models [104,105] where the weights or errors are proportional to the mean absolute error (log scale) or the mean percentage error (raw scale) reported for each model. Future users of these techniques should carefully consider whether the errors associated with each model presented here are compatible with their study design, given the effect sizes they are trying to detect. Specifically, the high r^2^ (0.75–0.77) for the best models presented here should not be interpreted in isolation outside the context of potential errors in the models. Other future directions include improving our knowledge of tissue densities and we discuss these next.

Detailed knowledge of tissue densities and how they change over time would greatly improve predictions of dry body mass [48]. This is because body proportions and estimated body volumes are related to body mass through density. Our approach in this study was to assume constant tissue density throughout the body but we relaxed this assumption by modeling some body parts independently of each other. However, the body is made of tissues of varying densities and water content including fat, muscle, cartilage, and bone [106–108]. Therefore, if we knew the average tissue densities or water content in different body regions, we could more accurately model the relationship between body volumes and wet or dry body mass. These relationships are further complicated by development, since changes in the volumes or densities of particular tissues in amphibians, like cartilage and bone [73], will affect the average tissue density or water content across body regions. For example, developmental changes in the relative proportion of bone and brain tissue in amphibians will change the average tissue density in the skull, in turn changing the relationship between skull volume and skull mass. This means the growth and development of tissues with different densities can alter the relationships between body proportions, volumes, densities, masses, and consequently, physiological rates. Notably, the relationship between metabolic rates and masses in mammals can vary across different tissues and this is likely due to differences in fat content or density [109,110]. Future research using micro-CT or similar methods would allow us to estimate the volumes of various tissues with distinct densities [111,112]. In summary, understanding the density of structures throughout the body and how they change through time is an important area of future research.

The application and future directions of this research should help to advance our understanding of biological processes. We recognize three key areas including identifying the sources of natural variation in physiological rates, the mechanisms underlying growth and metabolism, and the mechanisms linking populations with macroecological and macroevolutionary patterns. In the context of physiological ecology, learning how body mass channels physiological rates (potentially through environmental or biotic covariates) is a question of rich history and open inquiry, with metabolic rates perhaps among the most studied type of physiological rate across taxa [5,15,16,19,34,113,114]. We are still learning how rates might scale up to whole organisms and empirical research often struggles with high degrees of intraspecific variability [16,31,34,115]. Since physiological rates, including metabolic and growth rates, are often correlated with body size or are standardized using body size, understanding how mass indices, including dry mass, vary within and among tissue types, individuals, and species is necessary for deeper knowledge of the anatomical and physiological mechanisms that determine physiological rates. Importantly, recently developed methods allow the integration of intraspecific and interspecific data in a phylogenetic context [116] and the integration of intraspecific and interspecific data in combination with different body mass indices seems crucial in elucidating the mechanisms underlying growth and metabolic scaling. This is because each body mass index makes different assumptions about how body size and different tissues relate to metabolism through mass, density, and metabolic activity. For example, use of dry body mass in such models measures body size independently of water which does not metabolize. Outside the relatively limited context of physiological ecology, learning how physiological rates are regulated through biotic covariates like behavior seems a worthwhile endeavor for gaining an integrative understanding of the behavioral mechanisms that underlie physiological rates. Some examples include learning how metabolic rates, body temperature, and growth rates change due to environmental temperature and at different levels of parental resource provisioning, abilities to thermoregulate, or other behavioral events like hibernation [36,37,117–119]. Furthermore, with continued development of the described methods and sampling of adult amphibians and other vertebrates, we expect knowledge of dry body mass in combination with physiological rates and longitudinal models to yield new insights into the individual-level drivers of macroecological and macroevolutionary patterns, such as patterns of biogeography, ecogeographical gradients, and evolutionary trade-offs [15,31,120,121]. Finally, this research has potential applications for understanding the biology of aging, gestation, obesity, or sleep and how body mass and metabolic rate are related to various disorders and health outcomes in amphibians and other animals [25,33,122,123].

## Conclusion

Here we presented new models (regression equations) for estimating dry body mass throughout an organism’s lifetime using only photographs and morphological measurements. The proposed methods are flexible and provide users with an array of options to suit their particular logistical and analytical needs. We predict similar future studies will show how morphological measurements will yield accurate estimates of dry body mass in other developmental stages for *X. laevis* and in other animal species like amphibians, insects, or fishes. Specifically, extensions of the proposed methods might allow us to obtain dry body mass estimates across individual lifetimes (at the egg, tadpole, and adult stages). We argue that learning more about dry body mass and density across levels of biological organization and taxonomy has great capacity for advancing our understanding of physiological or behavioral ecology.

## Supporting information

S1 File Alternative Language Abstract. (PDF)

S1 Protocol Protocol used for embedding, imaging, and measuring tadpoles. (PDF)

S1 Text ANOVA tables for model fits and hyperparameter search results. (PDF)

S1 Fig Frontal view image of agarose-embedded tadpole. The only measurements obtained from the frontal view were the body width (longest distance from eye to eye) and body area.
(PDF)
