# Supplementary material for: Non-lethal imaging and modeling approaches for estimating dry mass in aquatic larvae
Source: PLoS One. 2026 Apr 17;21(4):e0345767. doi: 10.1371/journal.pone.0345767 (PMC13089712; doi:10.1371/journal.pone.0345767)
Supplement: S1 File — (PDF) [file pone.0345767.s001.pdf]

S1 Alternative Language Abstract

Non-lethal imaging and modeling approaches for estimating dry mass in aquatic larvae

Short Title: Estimating dry mass in aquatic larvae

Authors: Daniela Granados Frias[1], Najva Akbari[1], Lauren A. O'Connell[1], Bryan H. Juarez[1,2,*]

[1]Department of Biology, Stanford University, Stanford, CA 94305, USA

[2]Earth System Science Department, Stanford University, Stanford, CA 94305, USA

***Corresponding Author Email:** bryanhjuarez@gmail.com

Resumen

La masa corporal es crucial para la escalación y comparación de las tasas fisiológicas. En concreto, la masa corporal seca es importante para determinar la tasa metabólica de un organismo, ya que excluye el peso del agua, que es metabólicamente inactiva. Aunque obtener medidas repetidas de la masa corporal a lo largo de la vida de un individuo es sencillo, solo podemos obtener una única estimación de la masa corporal seca, ya que los métodos clásicos requieren la eutanasia del animal al final del experimento. En este estudio, presentamos técnicas de imagen y modelado para estimar la masa corporal seca individual en renacuajos de rana de uñas africana (*Xenopus laevis*), que permiten obtener medidas repetidas de los mismos individuos. Aplicamos principios alométricos y comprobamos si la anatomía externa proporciona estimaciones fiables de la masa corporal seca. En concreto, describimos un procedimiento para incrustar los renacuajos en un medio de agarosa para obtener datos morfológicos en 3D y despues evaluamos las predicciones de masa seca

mediante nueve modelos de máxima verosimilitud y aprendizaje automático con validación cruzada. El modelo más eficaz y flexible es un modelo alométrico que utiliza estimaciones del volumen corporal para predecir la masa corporal seca. Sin embargo, otros modelos basados en la masa corporal húmeda o con menos variables de entrada también pueden ser viables desde el punto de vista logístico. Esta investigación sienta las bases para futuras investigaciones sobre la importancia biológica de la masa corporal seca, particularmente en el contexto del desarrollo y la ecología fisiológica.
