# Supplementary material for: Non-lethal imaging and modeling approaches for estimating dry mass in aquatic larvae
Source: PLoS One. 2026 Apr 17;21(4):e0345767. doi: 10.1371/journal.pone.0345767 (PMC13089712; doi:10.1371/journal.pone.0345767)
Supplement: S1 Protocol — (PDF) [file pone.0345767.s002.pdf]

Jun 25, 2025

Non-lethal 3-D imaging techniques for estimating tadpole morphology

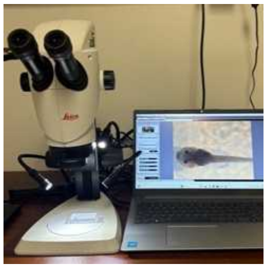

DOI
dx.doi.org/10.17504/protocols.io.rm7vzk1wrvx1/v1

Daniela Granados Frias[1], Najva Akbari[1], Lauren A O'Connell[1], Bryan Juarez[1,2]

[1]Department of Biology, Stanford University; [2]Department of Earth System Science, Stanford University

O'Connell Lab at Stanford

Najva Akbari
Stanford University

Create & collaborate more with a free account

Edit and publish protocols, collaborate in communities, share insights through comments, and track progress with run records.

Create free account

OPEN ACCESS

DOI: https://dx.doi.org/10.17504/protocols.io.rm7vzk1wrvx1/v1

Protocol Citation: Daniela Granados Frias, Najva Akbari, Lauren A O'Connell, Bryan Juarez 2025. Non-lethal 3-D imaging techniques for estimating tadpole morphology. **protocols.io** https://dx.doi.org/10.17504/protocols.io.rm7vzk1wrvx1/v1

License: This is an open access protocol distributed under the terms of the **Creative Commons Attribution License**, which permits unrestricted use, distribution, and reproduction in any medium, provided the original author and source are credited

Protocol status: Working
We use this protocol and it's working

Created: January 17, 2025

Last Modified: June 26, 2025

Protocol Integer ID: 118650

Keywords: Xenopus, anatomy, Fiji, ImageJ, microscopic, dry mass, ontogeny, estimating tadpole morphology, tadpole morphology the majority, tadpole, anesthetizing small aquatic animal, imaging biological specimen, small aquatic animal, biological specimen, various morphological measurement, obtaining various morphological measurement, measurements of dry body mass, imaging, animal, dry body mass, physiological study, stitching image, image, most imaging protocol, drying individual, composite image, euthanasia

Funders Acknowledgements:
Stanford Biology Summer Undergraduate Research Program, New York Stem Cell Foundation, National Institutes of Health
Grant ID: N/A

Abstract

The majority of protocols for imaging biological specimens are lethal and this raises both ethical and scientific concerns. One major limitation of most imaging protocols is that they prohibit studies which span the ontogenetic development of individuals. Here, we present a non-lethal protocol for ethically anesthetizing small aquatic animals (tadpoles), embedding them in an agarose matrix, photographing them in 3-D, and stitching images to form a single composite of larger individuals. We also describe methods for obtaining various morphological measurements (lengths, surface areas, volumes) and a euthanasia technique for drying individuals to obtain measurements of dry body mass, which is an important metric associated with metabolism used in physiological studies. Expected results include high-quality single and composite images of tadpoles ~10-30 mm long viewed from 3 dimensions (dorsal, lateral, frontal) of various morphological measurements.

Materials

Tricaine methanesulfonate (MS-222) Solution: 0.04g MS222, 0.06g bicarbonate sodium, 60 mL DI water.
Agarose Solution
Tadpoles (e.g., stages 44–54)
Chambered coverglass
Detail paint brushes
Large petri dishes
Small petri dishes
Glass slides
Long forceps
Plastic pipettes
5mL graduated cylinder

Troubleshooting

Safety warnings

This protocol involves placing live animals in agarose media heated to 42 Celsius and it is crucial that the media is not hotter to avoid unnecessary discomfort for the animals.

Ethics statement

Prior ethics approval is necessary for using this protocol. We designed this protocol with prior approval from Stanford University under the Administrative Panel on Laboratory Animal Care (APLAC) Protocol #33097.

Before start

Before starting, please make sure to review the materials section and make sure you have all necessary resources to complete the protocol. Some may find writing dilutions by hand or practicing them prior to experimentation is helpful.

Preparing the Materials Needed

1 Liquify a 1.5% agarose solution by heating it up in the microwave in intervals of 30 seconds, stirring it in between each interval.

2 Once fully liquified, move the agarose container onto a hotplate set at 42–45 Celsius.

3 Add a stir bar to the agarose and set the RPM to about 140 such that the agarose solution is being stirred adequately.

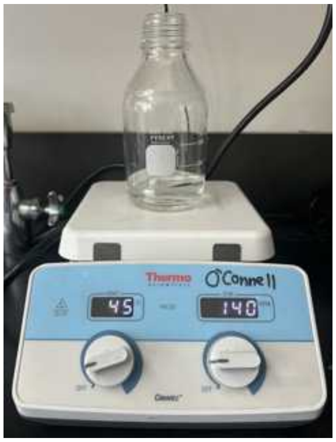

3.1 Measure the temperature of the agarose before using to make sure it's cooled to 42C to ensure that it does not hurt the tadpoles.

4 Prepare a buffered 0.03% MS-222 solution and cover it with aluminum foil to ensure that the sunlight doesn't break down the compound.

4.1 To neutralize the acidity of MS-222, and thus increase its safety, current recommendations include buffering with sodium bicarbonate ($NaHCO_3$) at a ratio of 2 parts sodium bicarbonate:1 part MS-222, on a dry weight basis.

5 Label a large and small Petri dish.

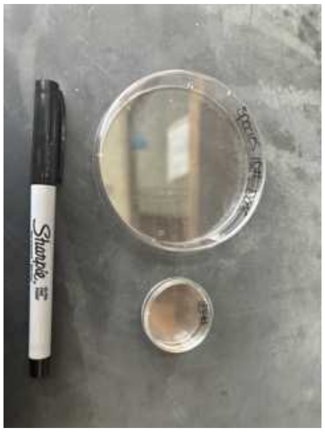

5.1 Using a permanent marker, label the small Petri dish with the tadpole ID.

5.2 Using a permanent marker, label the large Petri dish with the tadpole ID and the date.

Embedding

6 Transfer a tadpole into 0.03% MS222 solution for about 5 minutes to ensure that it is anesthetized. This can be verified by touching the tadpole with a transfer pipette and seeing if it responds by twitching.

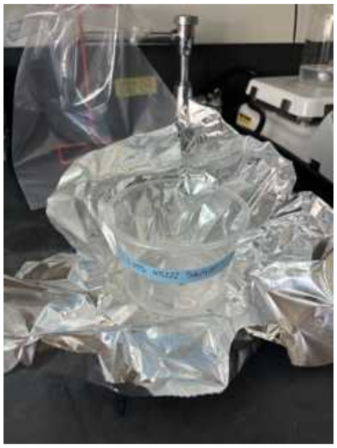

Note: Additional time in MS-222 might be necessary for larger tadpoles, but *Xenopus laevis* tadpoles (stages 44-54) did not require additional time in MS-222.

7 Use a pipette to fill a chambered coverglass with agarose solution about a third of the way full.

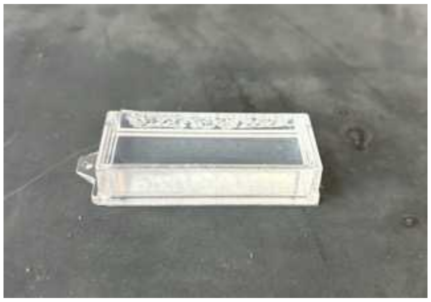

8 Once the agarose solution has solidified and the tadpole is completely anesthetized, fill up the chamber with liquid agarose to the top of the chamber and add the tadpole.

9 While the top layer of agarose is still liquid, use a detail paint brush to align the tadpole to the top right corner of the chamber making sure that the tadpole is straightened.

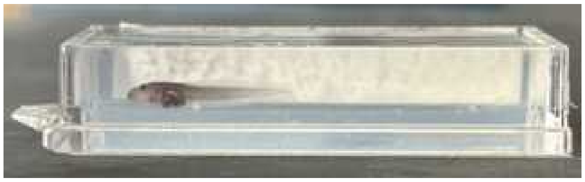

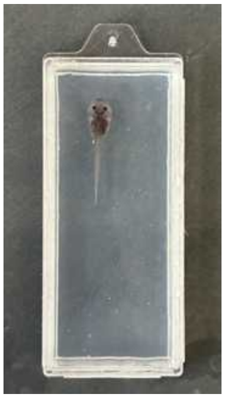

Imaging

10 Plug in the Leica microscope camera USB into the computer.

11 Open LAS EZ program once the microscope lights are green indicating it has successfully connected to the computer.

12 Reset the exposure adjust settings to the automatic settings in LAS EZ.

13 Ensure that the microscope is set up correctly with the following steps:

13.1 Adjust the height to maximize the focus evenly across the tadpoles tail, which is relatively more difficult to image the body due to its transparency.

13.2 Set the magnification to 0.6x, or a magnification appropriate for your animal.

13.3 Turn on the lights and set them at a constant brightness throughout experimentation.

13.4 Adjust the lighting such that there is even lighting and no shadows at each angle when viewed from above. Changes in the outside environment should be inconsequential for the image captured.

14 Place the tadpole chamber on the magnification table and center it such that the full tadpole is visible.

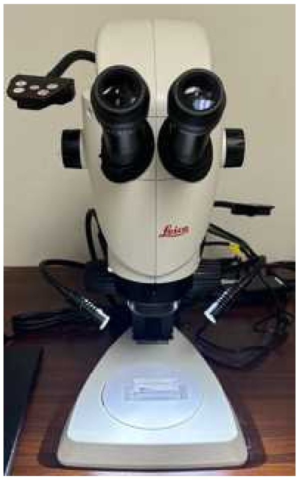

14.1 If the tadpole is too large to fit within the imaging window, take two separate images of the same tadpole such that the two images combined show the entire tadpole. If using >1 image to photograph the tadpole, minimizing lighting differences and keeping movement along a single direction will give the best results when combining >1 images into a single image.

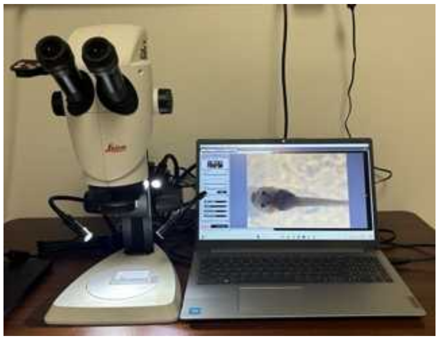

15 Start off by imaging the dorsal view of the tadpole.

16 In LAS EZ program, select "acquire image" and wait for the image to be captured and loaded.

17 Select "Process" tab at the top of the page.

17.1 Go to the information section and add a description including the species, ID, and view type (dorsal (D), lateral (L), and frontal (F).

18 Double click on the image on the bottom of the screen to display an options menu.

18.1 Select "Rename Current Image".

18.2 Name the image with the following format: TadpoleID_View#.

18.3 Click "OK".

19 To the rightmost select "save the currently displayed image".

20 Move the tadpole into the lateral position.

21 Adjust the height of the microscope lens so that the image is once again in focus.

22 Repeat steps 16-19.

23 Move the tadpole into the frontal position.

24 Adjust the height of the microscope lens so that the image is once again in focus.

25 Repeat steps 16-19.

De-embedding

26 After imaging, carefully remove the tadpole from the agarose and place it into a water container to clean off any excess agarose clinging onto the tadpoles body.

27 Place a small plastic boat on a scale and zero it.

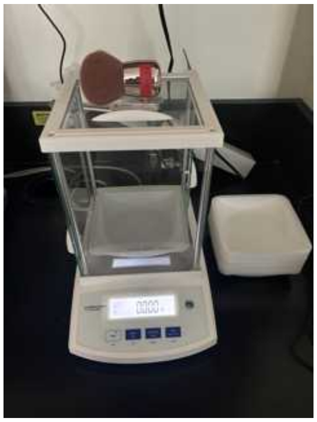

28 Once the tadpole is clean, place the tadpole into a Petri dish and gently pat dry with a Kimwipe.

29 Place the tadpole into the plastic boat and acquire the wet mass.

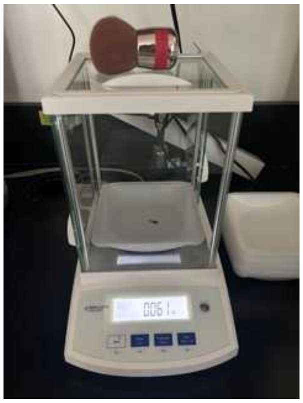

30 Place the tadpole into a 5ml graduated cylinder containing 2 ml of water and obtain the volume by adding the tadpole into the graduated cylinder and calculating the volumetric displacement.

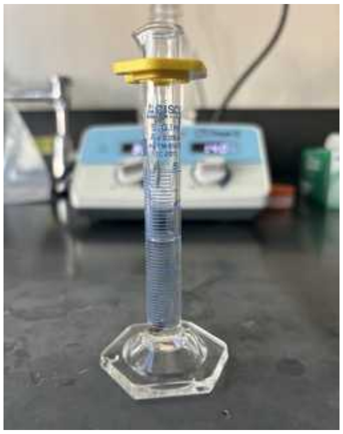

Euthanizing the Tadpoles

31 Using forceps place the small Petri dish into liquid nitrogen until it reaches equilibrium.

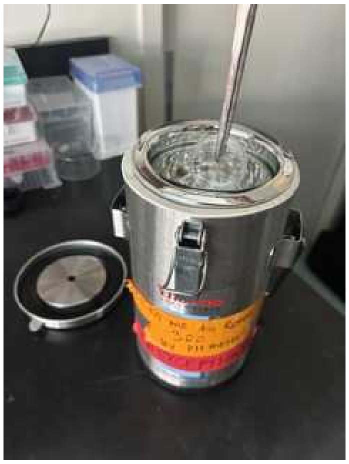

32 Remove the Petri dish from the nitrogen and place it into an insulating container.

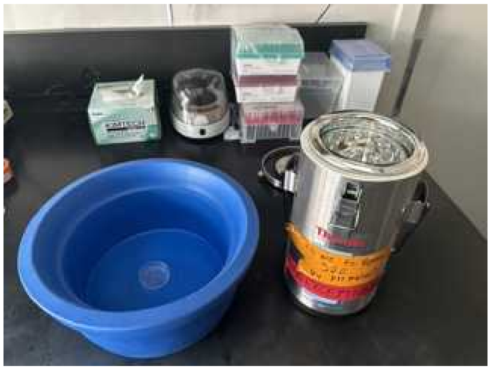

33 Take the cover off of the Petri dish and fill the Petri dish with liquid nitrogen.

34 Remove the tadpole from the graduated cylinder.

35 Gently dry off the tadpole with a Kimwipe.

36 Using tweezers, gently place the tadpole into the small Petri dish containing the liquid nitrogen.

37 Using the forceps, place the cover back onto the Petri dish.

38 Fill the larger insulting container up to half of the height of the Petri dish with liquid nitrogen and cover it with a lid.

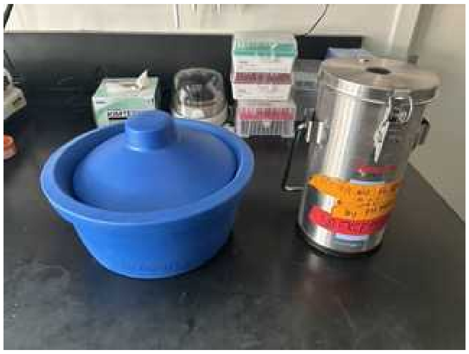

39 After 15 minutes open the lid and remove the Petri dish with the forceps.

40 Place a glass slide onto a scale and obtain the mass.

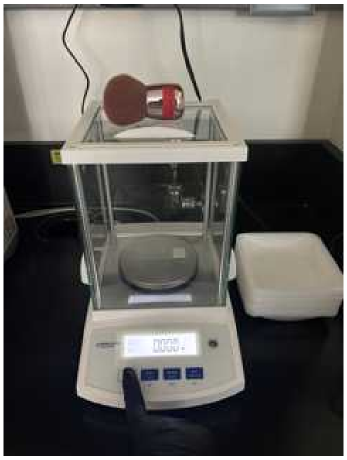

41 Use tweezers to remove the tadpole from the Petri dish and place it onto the pre-weighed glass slide to obtain a second wet mass.

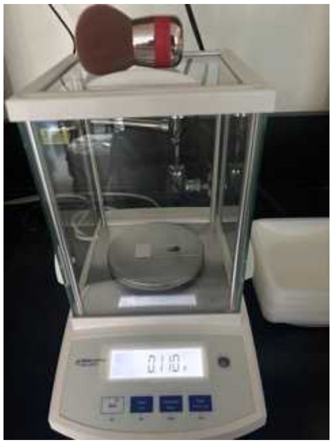

42 Place the tadpole along with the glass slide into a large Petri dish.

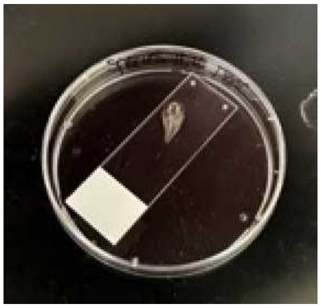

43 Place this Petri dish into the drying oven set at 37 Celsius.

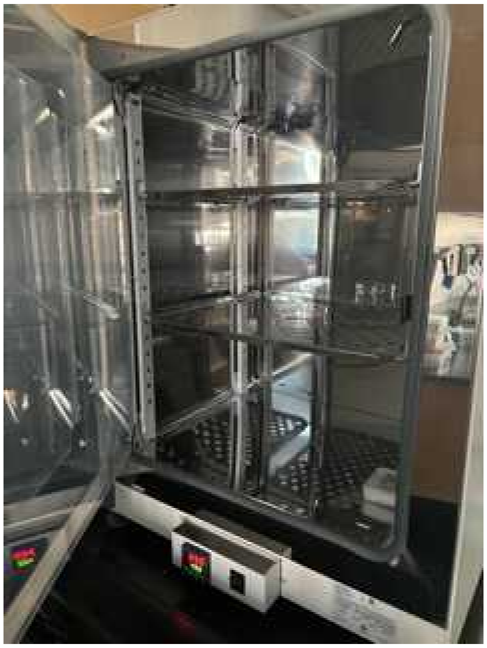

44 Allow the tadpole to dry in the oven for 72 hours.

45 Obtain the mass of the glass slide with the tadpole after every 24-72 hours as necessary to obtain the final dry mass. We found all tadpoles dried completely within 48 hours.

Fiji Measurements

46 Open Fiji (Schindelin et al. 2012).

47 Some tadpoles are too large to be photographed in a single image, depending on the tadpole size and the magnification used. For such cases, we may stitch together two separate images with overlapping regions.

47.1 Load the images to be stitched into Fiji and select Pairwise Stitching from Plugins>Stitching (Preibisch et al. 2009).

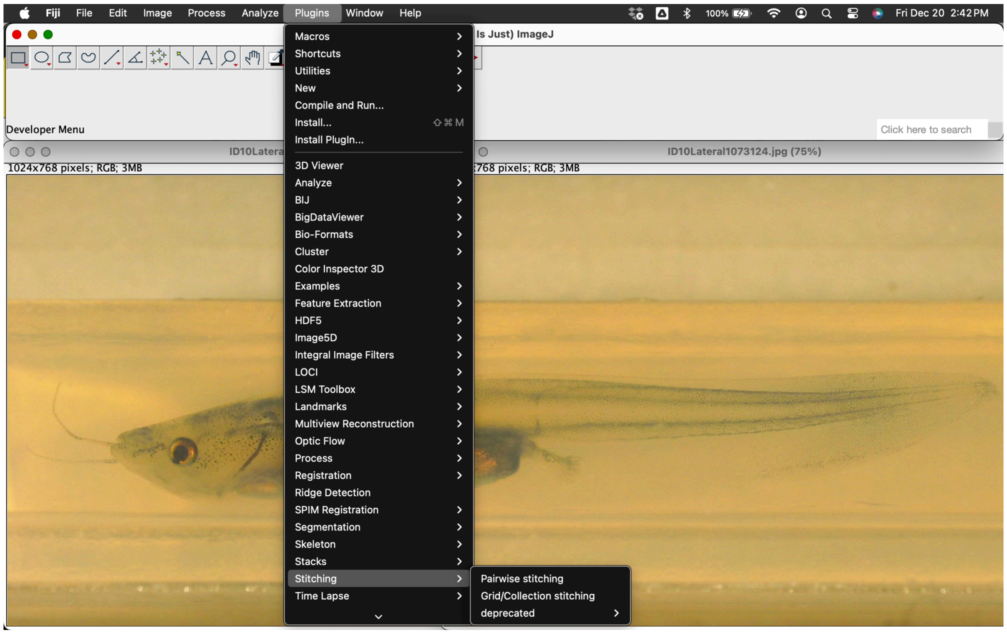

47.2 Confirm the images to be stitched.

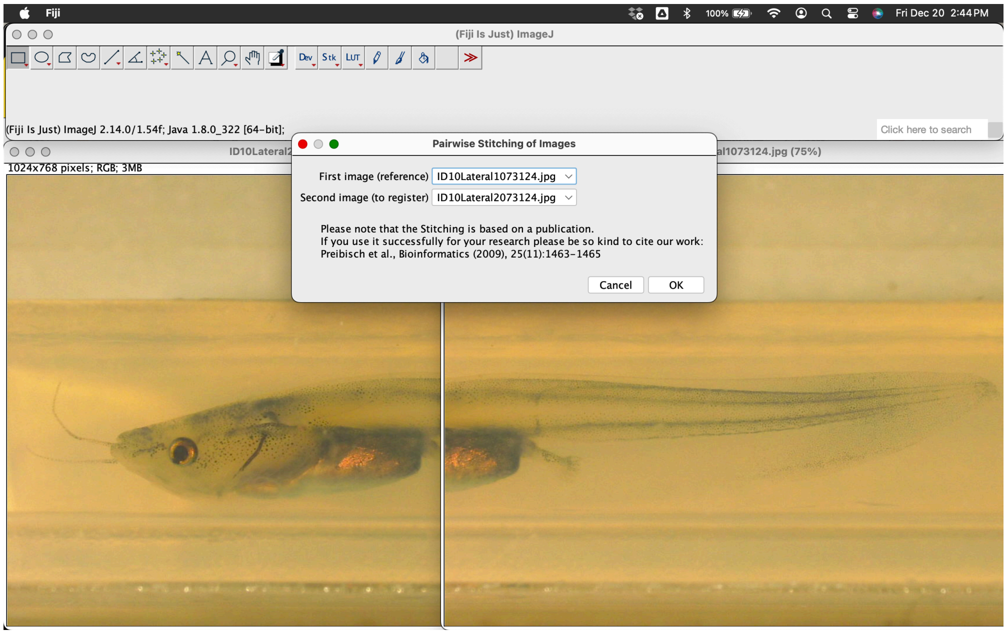

47.3 The linear blending and average methods (100 peaks) gave good results. This depends on the overlapping area of the two images being stitched and the similarity of their RGB pixel channels.

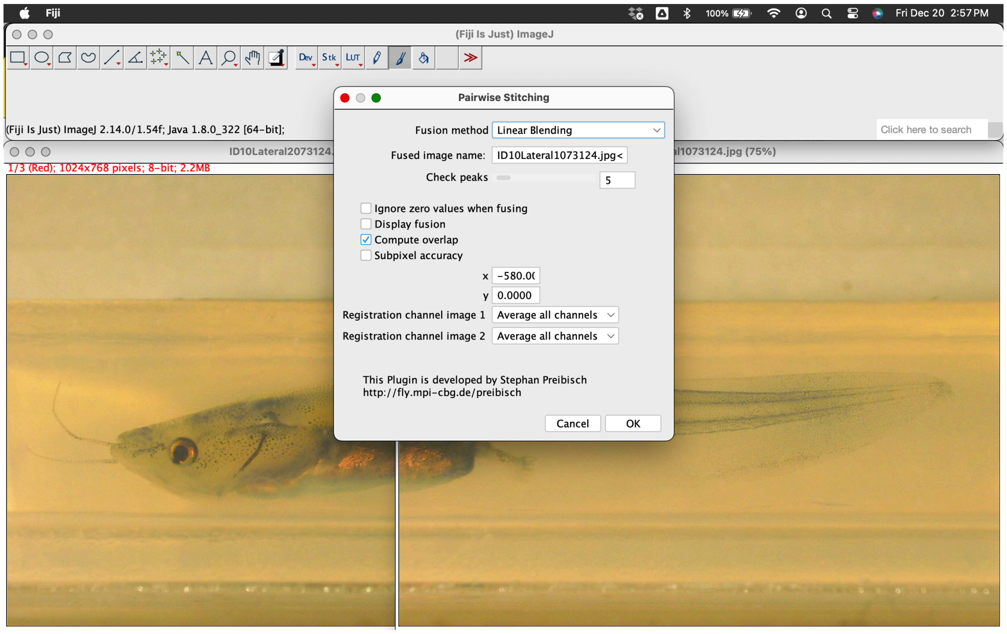

47.4 After pressing "OK", the final stitched images is shown and the stitching correlation is logged. Note: stitching correlations are sensitive to differences in lighting, so the lighting must remain as uniform as possible while taking the original images. We recommend adjusting the positioning/lighting conditions when stitching images so that the correlation among the shared areas between two images being stitched is >0.95.

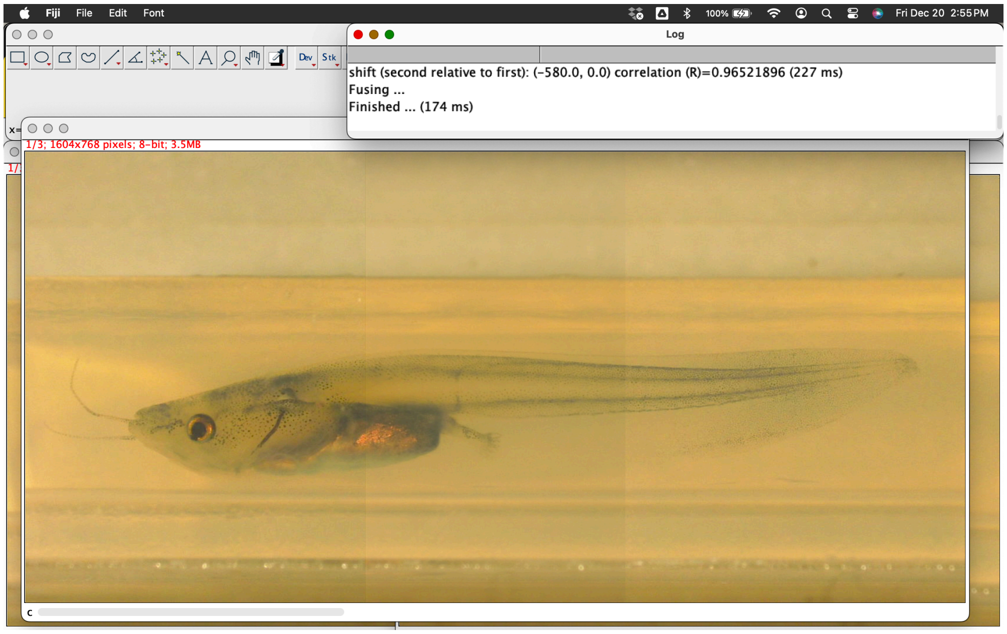

48 Calibrate the measuring tool.

48.1 Select the straight line tool on the menu bar.

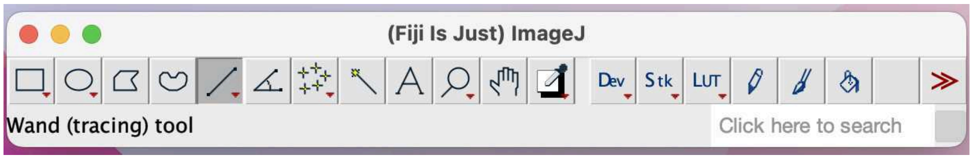

48.2 Drag an image with known distances into the program.

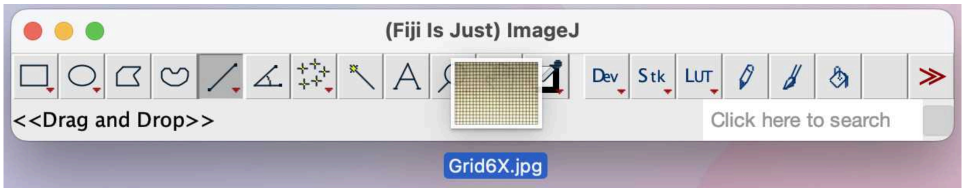

48.3 Click and drag the pointer to draw a line over a known distance.

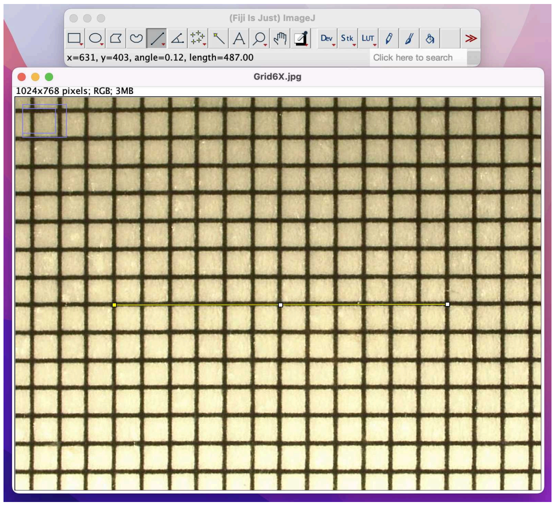

48.4 Go to the 'Analyze' tab.

48.5 Click on 'select scale'.

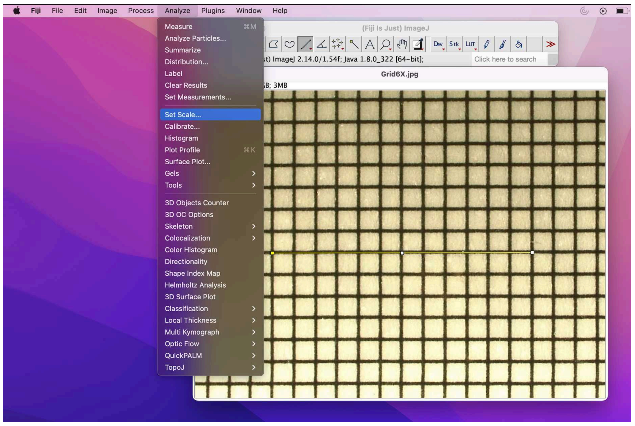

48.6 Enter the known distance and its unit in the 'Known distance' and 'Unit of length'.

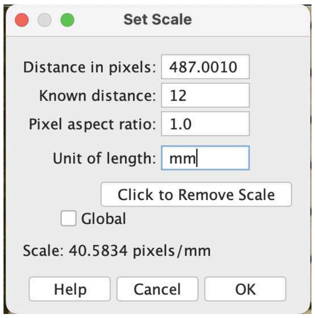

48.7 Click the box next to 'Global' and select 'OK'.

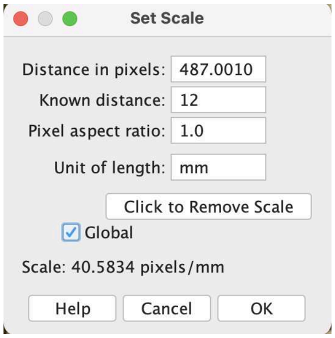

49 Obtain the dorsal Measurements.

49.1 Drag the dorsal image into Fiji.

49.2 Once, the image has been opened through the program, select the segmented line tool.

49.3 Draw a segmented line starting at the tip of the snout going through the middle of the brain and ending at the midpoint of the spine where the body ends at the tail begins.

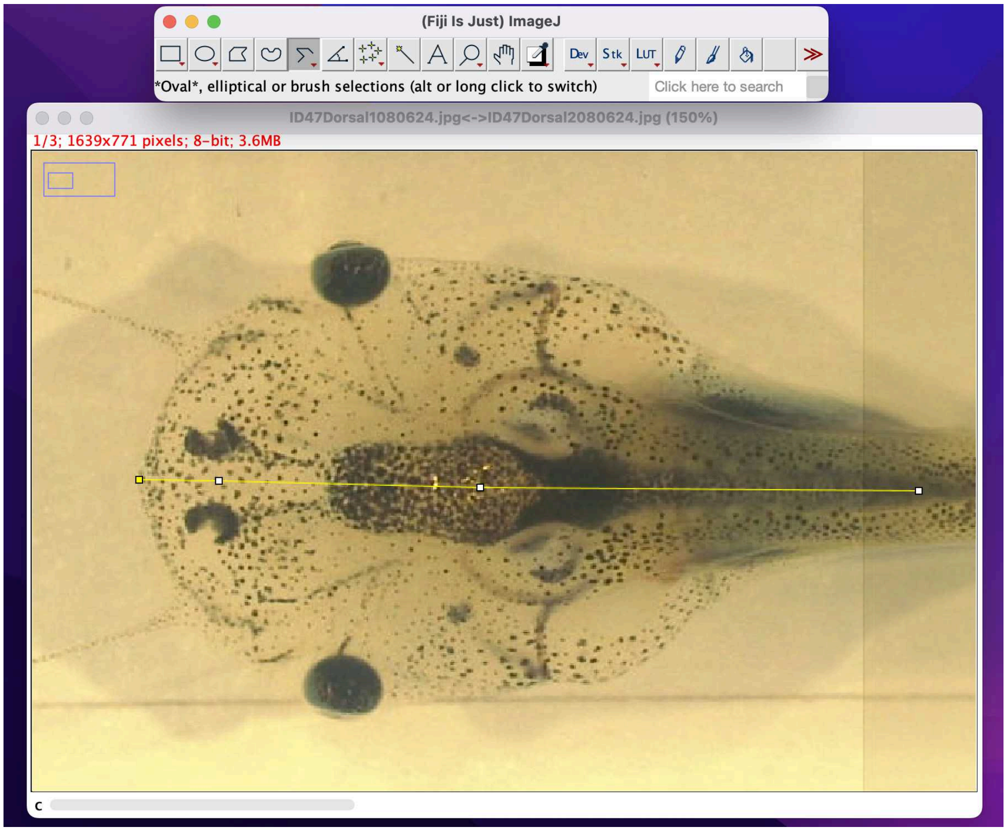

49.4 Click 'command' + 'M' to measure the line. This will give the body length.

49.5 Select the straight line tool.

49.6 Draw a line starting and ending right after the eyes.

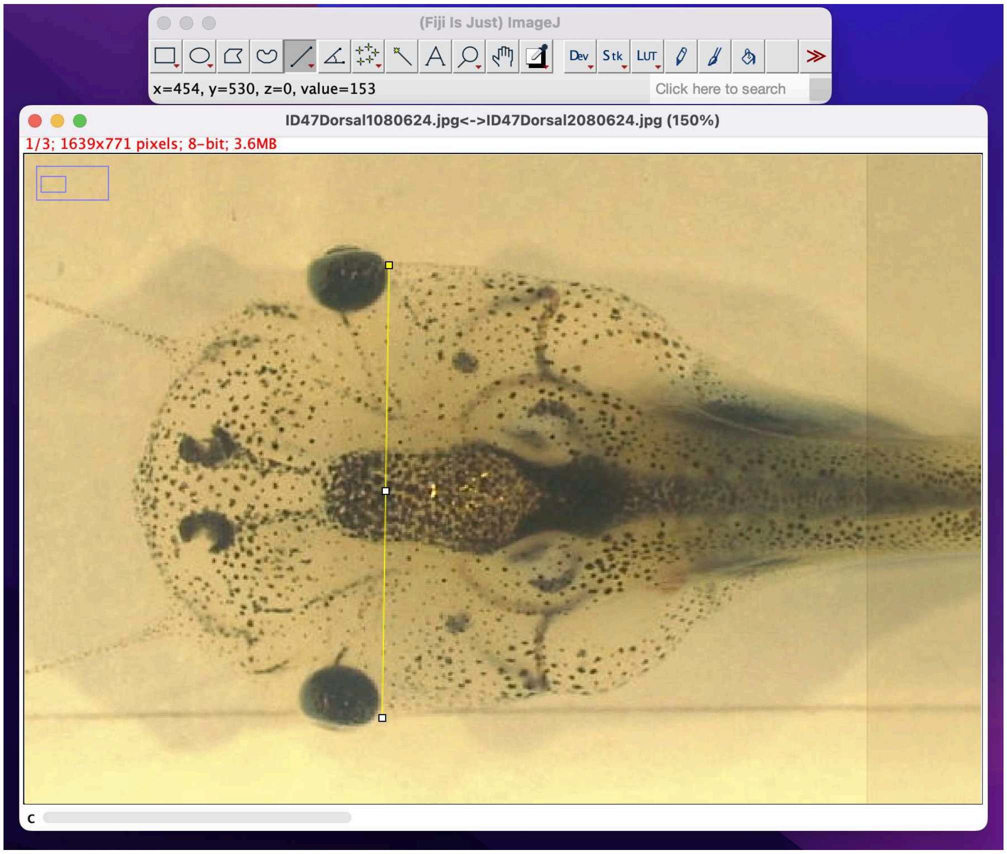

49.7 Click 'command' + 'M' to measure the line. This will give the body width.

49.8 Select the 'polygon selections' tool.

49.9 Carefully select points along the body of the tadpole. The number of points should be kept to a minimum while ensuring that the shape of the tadpole is being accurately portrayed. Using more points than necessary introduces measurement error.

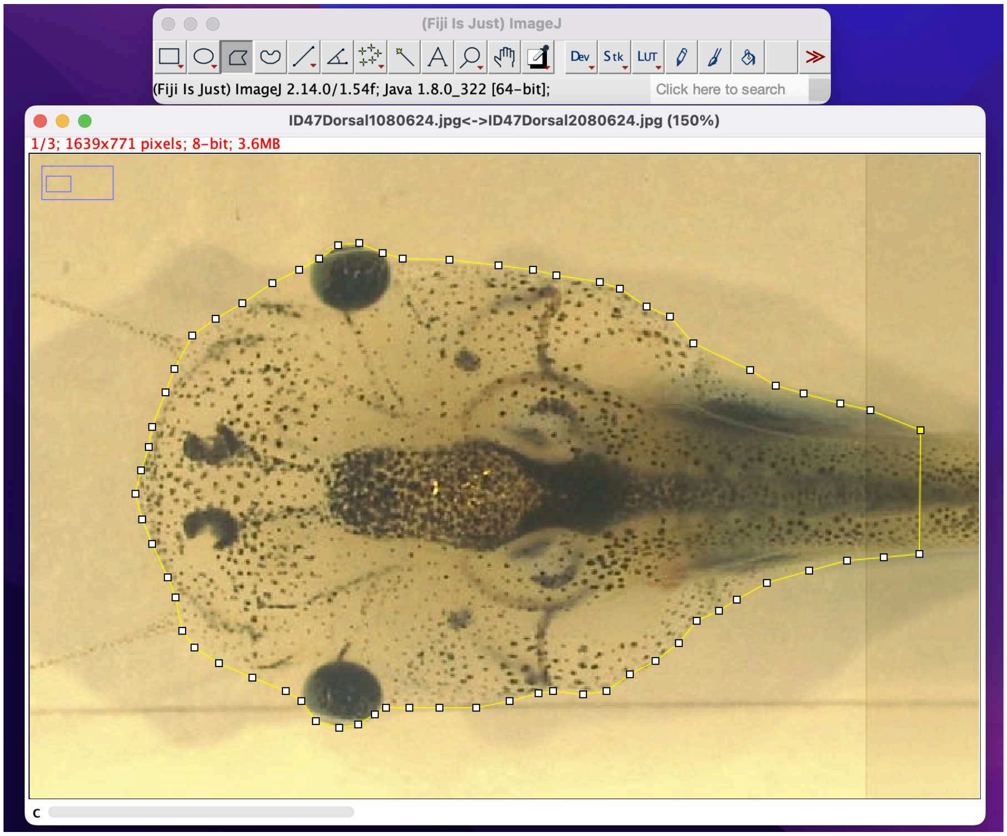

49.10 Once all of the point are set, click 'command' + 'M' to calculate the body area.

49.11 Select the segmented line tool.

49.12 Draw a segmented line following the spine alignment.

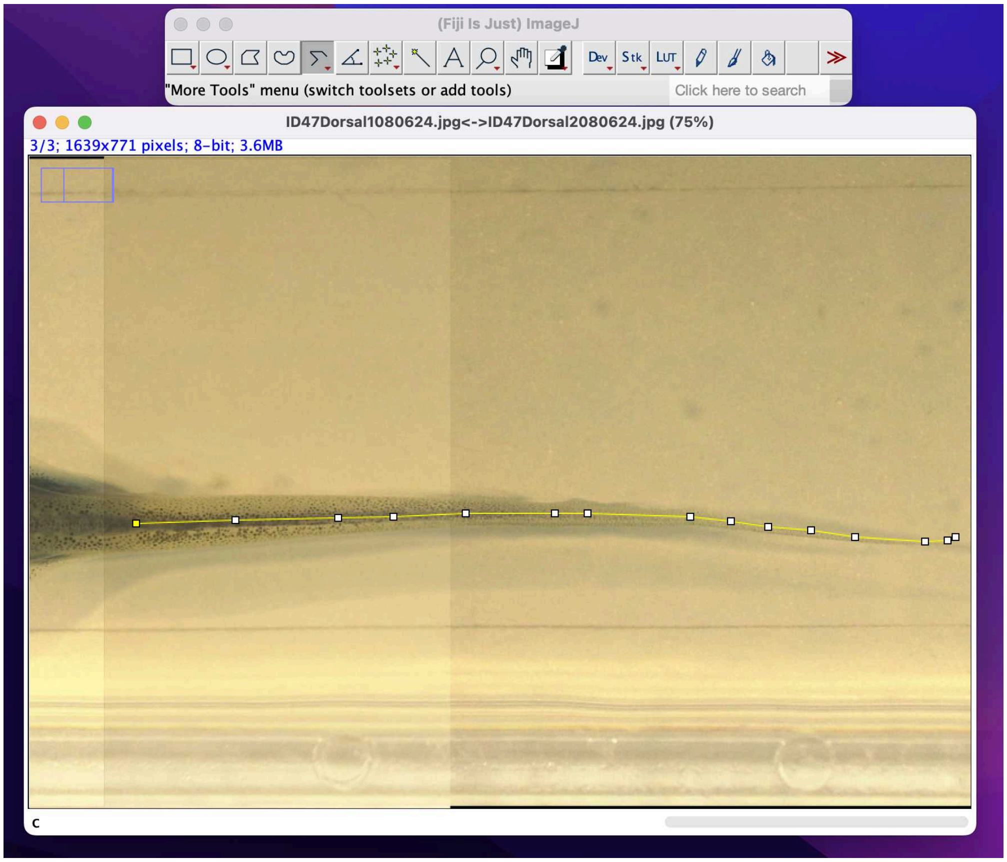

49.13 Click 'command' + 'M' to measure the line. This will give the tail length.

49.14 Select the straight line tool.

49.15 Draw a line from the outermost edge of the start of the tail to the outermost edge on the opposite side of the tail.

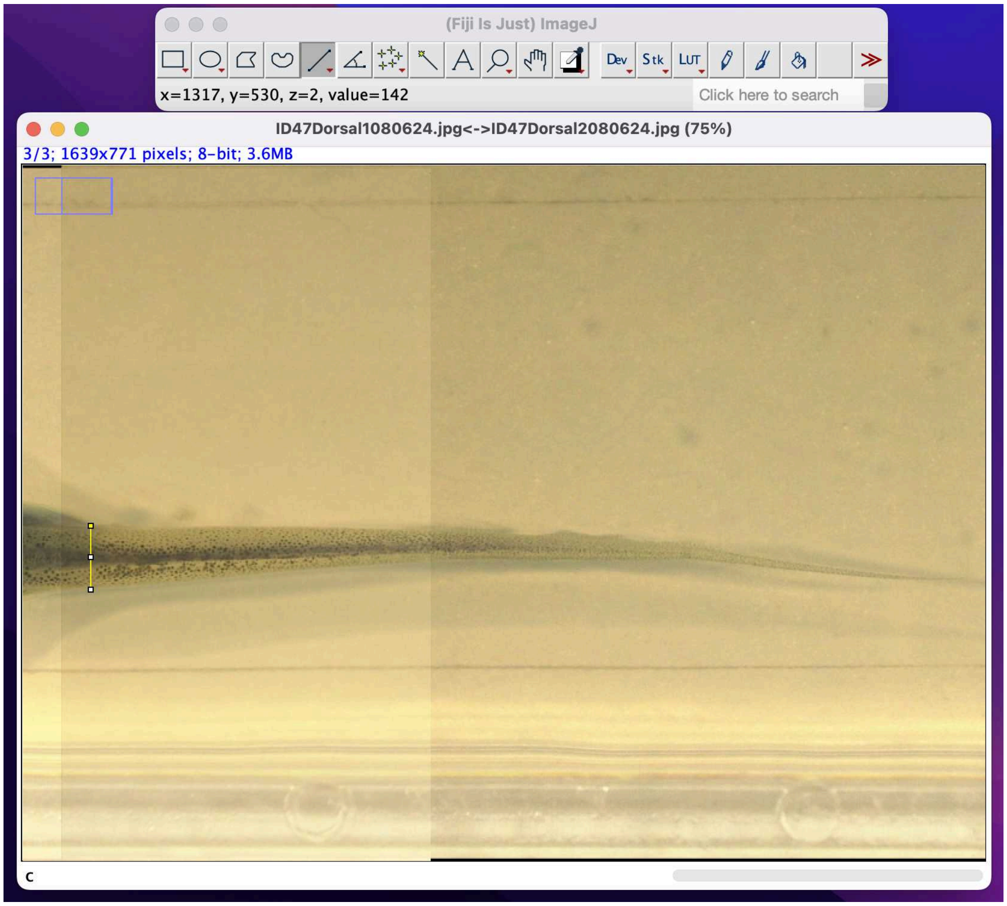

49.16 Click 'command' + 'M' to measure the line. This will give the tail width.

49.17 Select the 'polygon selections' tool.

49.18 Carefully select points along the tail of the tadpole. The number of points should be kept to a minimum while ensuring that the shape of the tadpole is being accurately being portrayed.

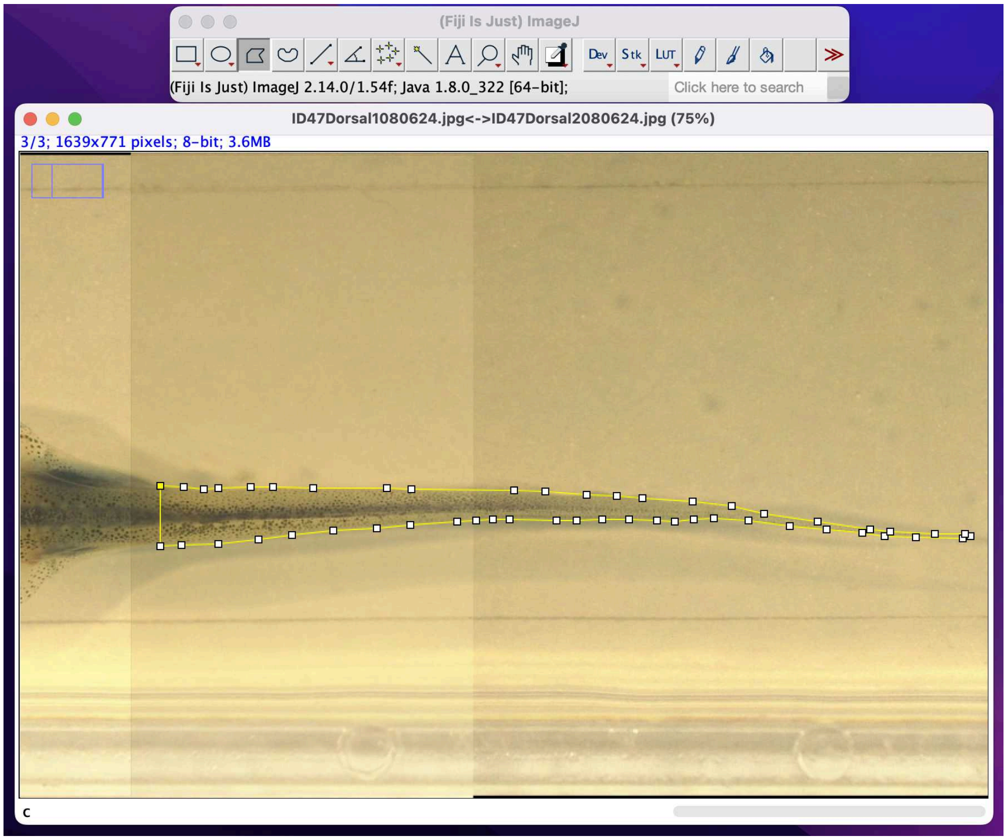

49.19 Once all of the point are set, click 'command' + 'M' to calculate the tail area.

50 Save the measurements.

50.1 Select 'file'.

50.2 Select 'save as'.

50.3 Name the file with the following format: ID#.ViewMeasurements.

50.4 Select 'save'.

51 Obtain the lateral measurements.

51.1 Drag the lateral image into Fiji.

51.2 Once, the image has been opened through the program, select the segmented line tool.

51.3 Draw a segmented line starting at the tip of the snout, going through the midline of the animal, and ending at the midpoint of the spine where the body ends and the tail begins.

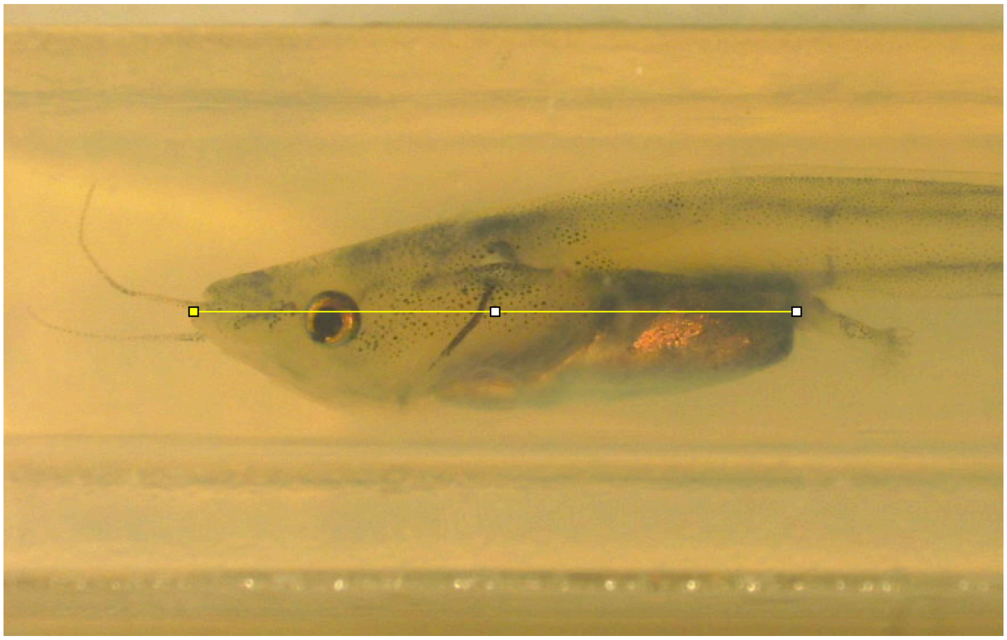

51.4 Click 'command' + 'M' to measure the line. This will give the body length.

51.5 Select the straight line tool.

51.6 Draw a line starting at the outermost part of the head right after the eye ends and end the line at the bottommost part of the body directly below the eye.

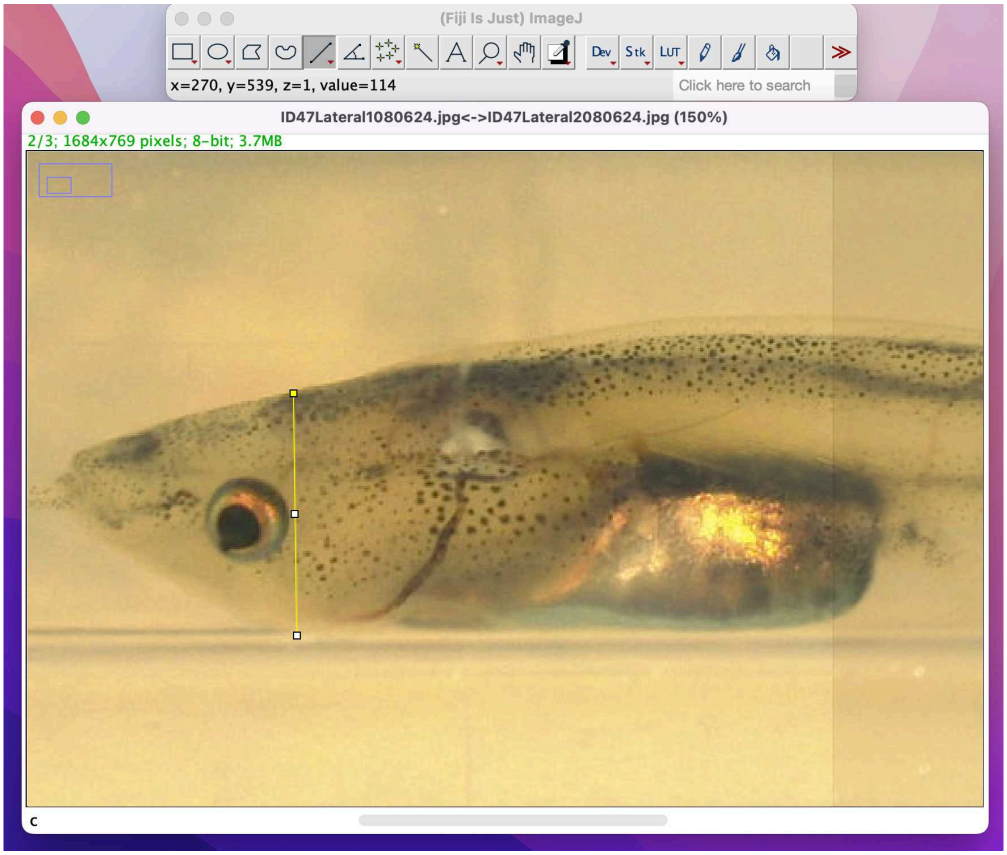

51.7 Click 'command' + 'M' to measure the line. This will give the body height.

51.8 Select the 'polygon selections' tool.

51.9 Carefully select points along the body of the tadpole. The number of points should be kept to a minimum while ensuring that the shape of the tadpole is being accurately being portrayed.

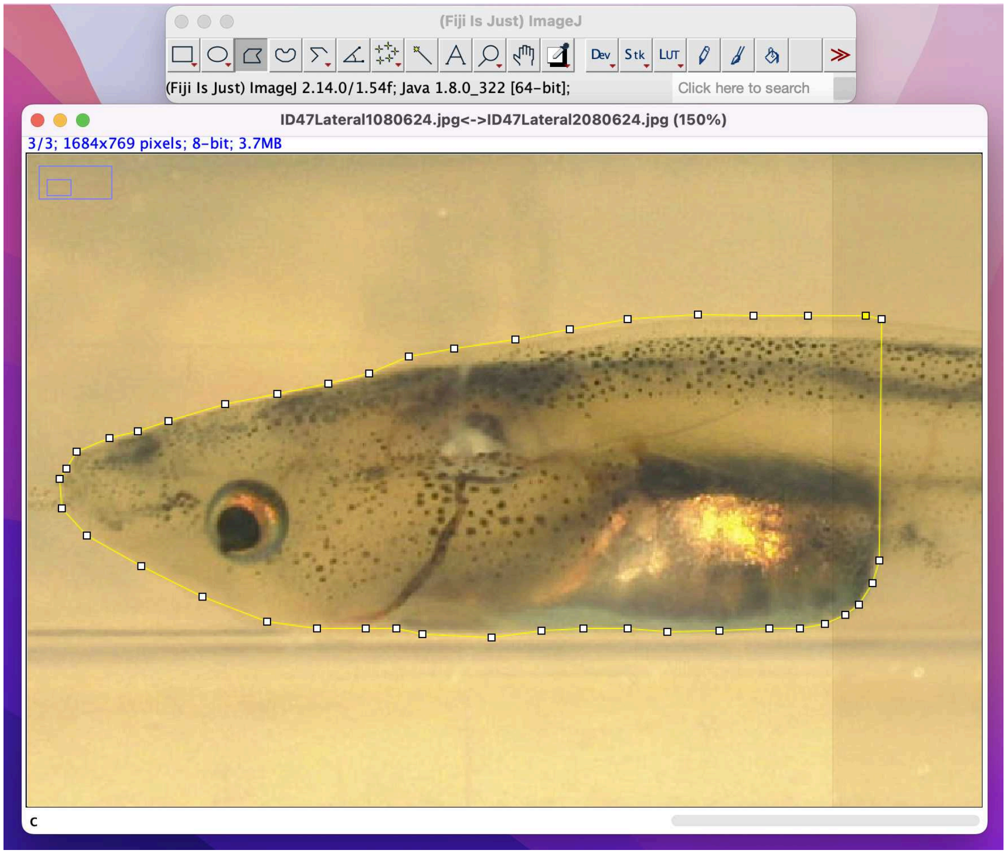

51.10 Once all of the point are set, click 'command' + 'M' to calculate the body area.

51.11 Select the segmented line tool.

51.12 Draw a segmented line following the spine.

51.13 Click 'command' + 'M' to measure the line. This will give the tail length.

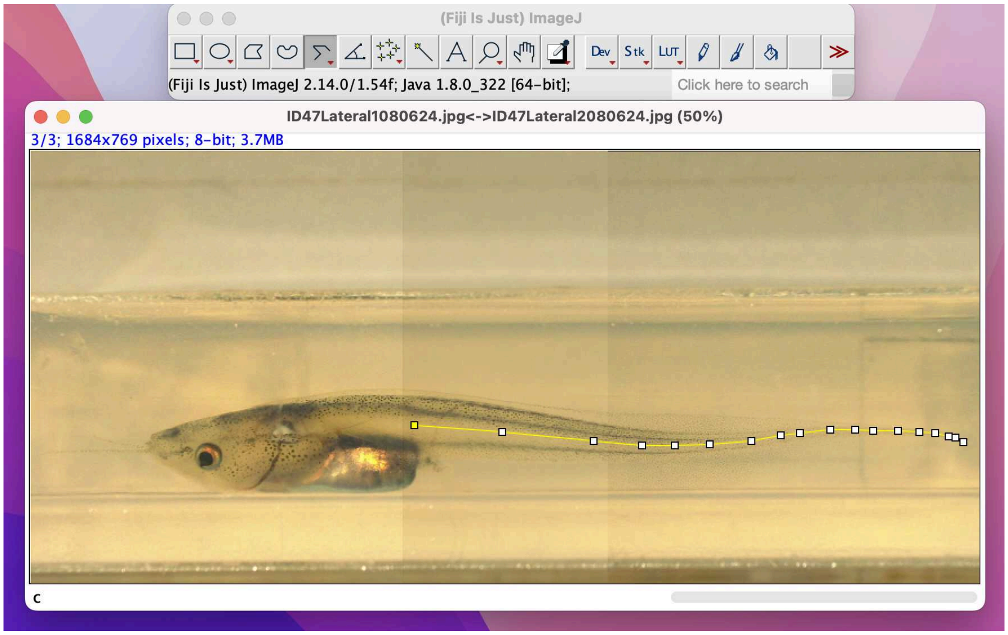

51.14 Select the straight line tool.

51.15 Draw a line from the uppermost edge of the start of the tail to the bottommost edge on the opposite side of the tail.

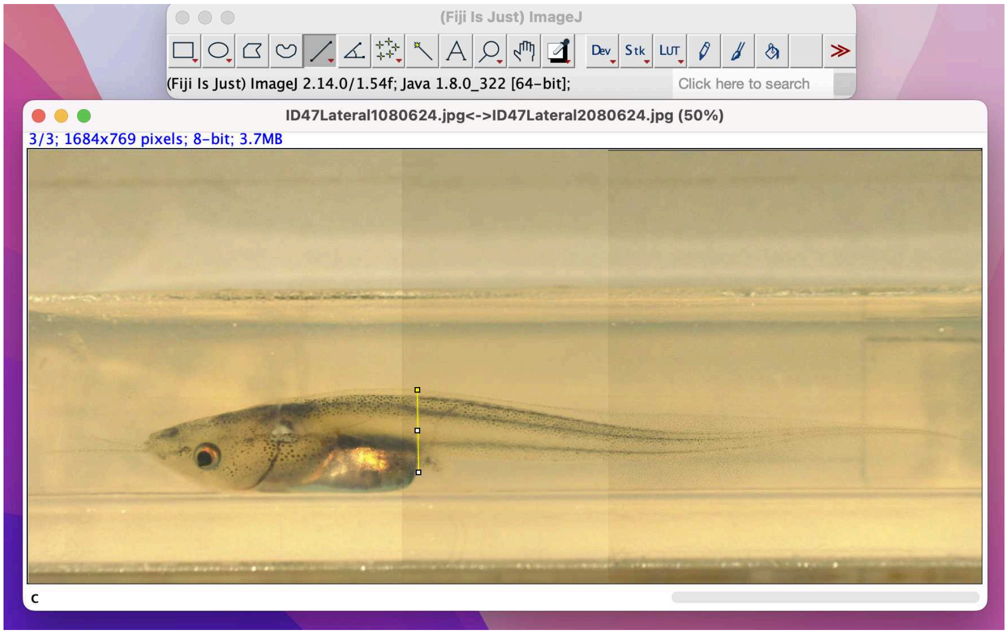

51.16 Click 'command' + 'M' to measure the line. This will give the tail height.

51.17 Select the 'polygon selections' tool.

51.18 Carefully select points along the tail of the tadpole. The number of points should be kept to a minimum while ensuring that the shape of the tadpole is being accurately being portrayed.

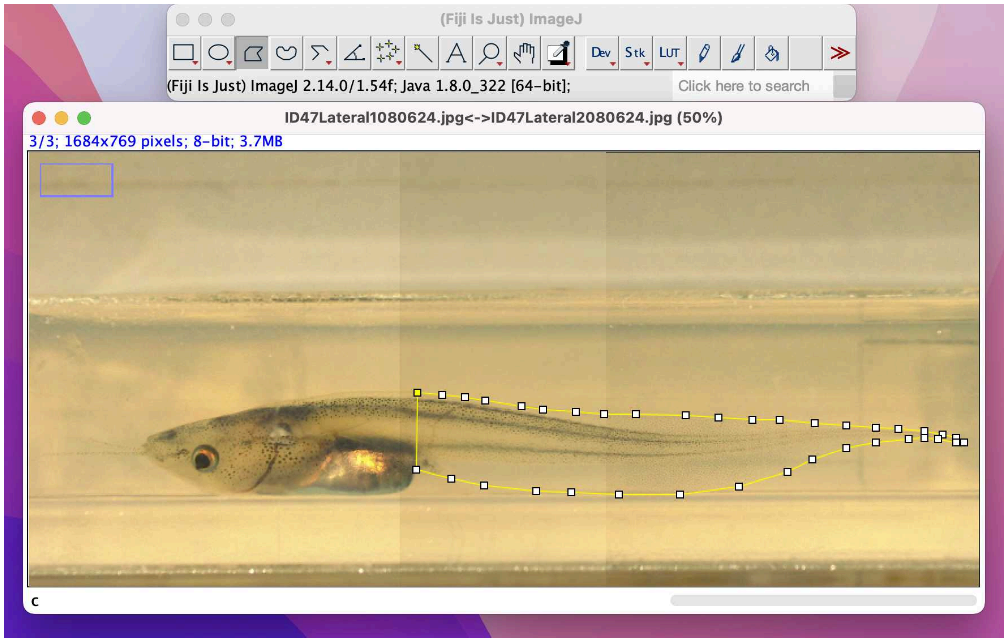

51.19 Once all of the point are set, click 'command' + 'M' to calculate the tail area.

51.20 While still using the polygon selection tool, carefully select points along the dark edges of the tail. The number of points should be kept to a minimum while ensuring that the shape of the tadpole is being accurately being portrayed.

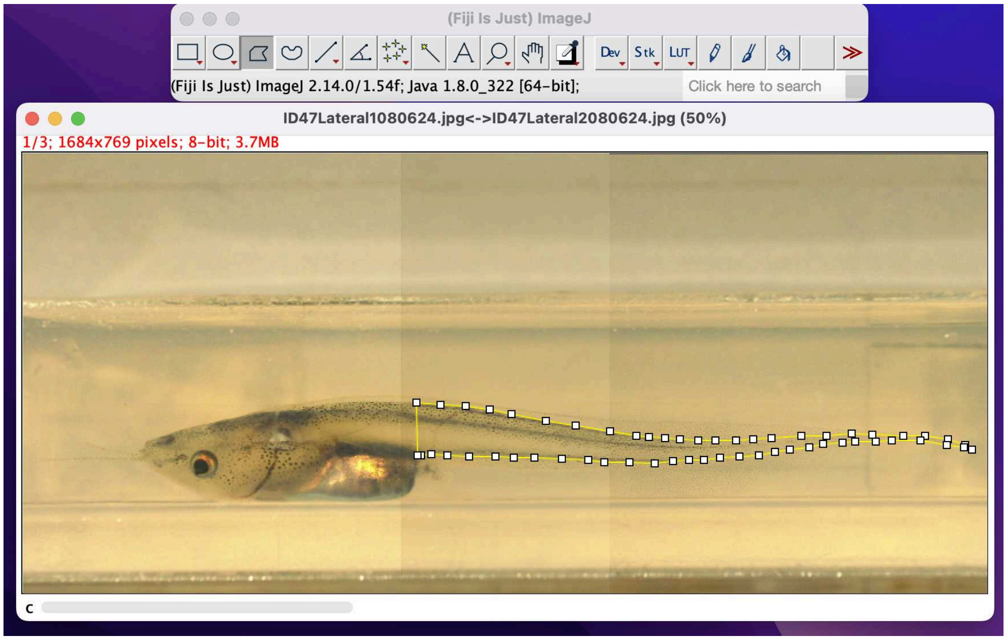

51.21 Once all of the point are set, click 'command' + 'M' to calculate the tail muscle area.

51.22 While still using the polygon selection tool, carefully select points along the limb bud, if present . The number of points should be kept to a minimum while ensuring that the shape of the tadpole is being accurately being portrayed.

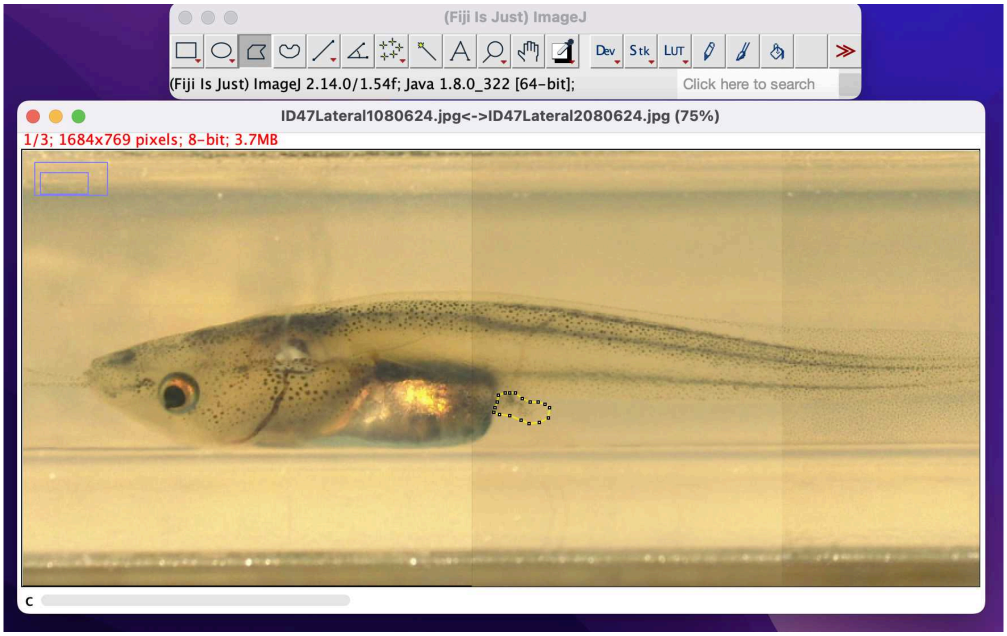

51.23 Once all of the point are set, click 'command' + 'M' to calculate the limb bud area.

52 Repeat step 49 for this set of measurements.

53 Obtain frontal measurements.

53.1 Drag the frontal image into Fiji.

53.2 Once, the image has been opened through the program, select the segmented line tool.

53.3 Click 'command' + 'M' to measure the line. This will give the body height.

53.4 Select the straight line tool.

53.5 Draw a line starting at the leftmost part of the tadpoles head at the center of its eye and ending at the rightmost edge of the tadpoles right eye.

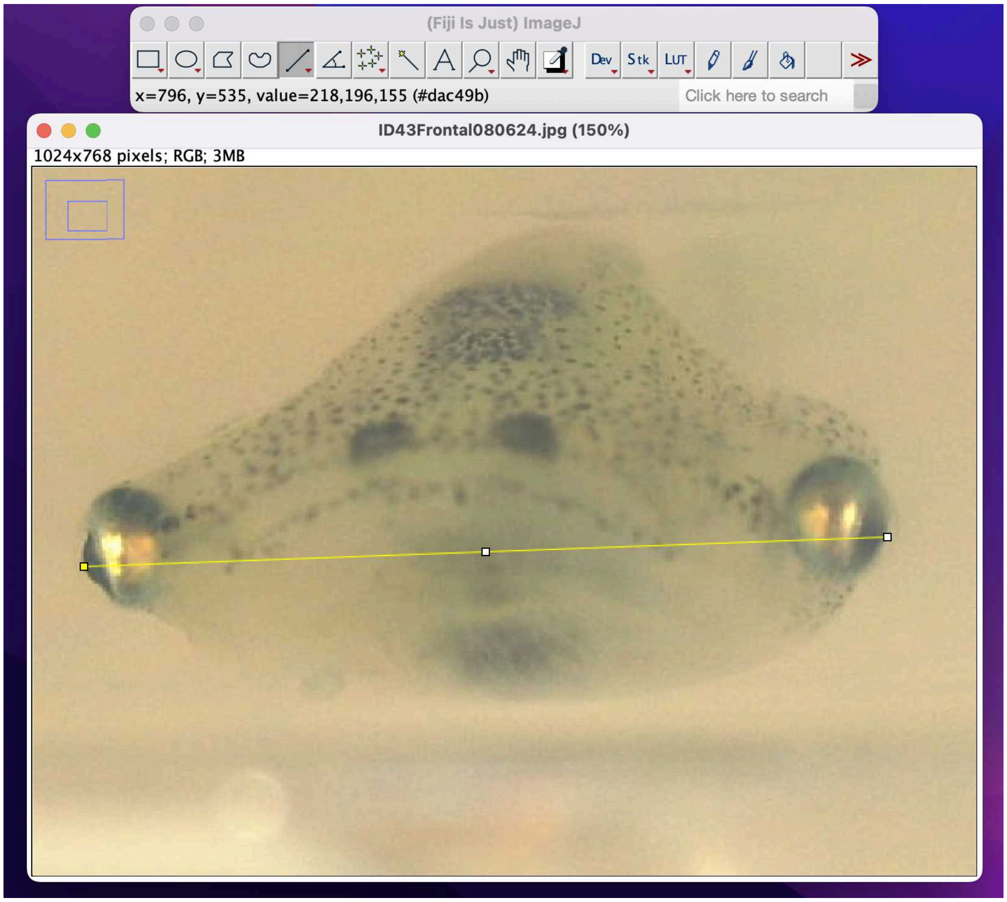

53.6 Click 'command' + 'M' to measure the line. This will give the body width.

53.7 Select the 'polygon selections' tool.

53.8 Carefully select points along the body of the tadpole. Keeping the number of points to a minimum while ensuring that the shape of the tadpole is being accurately being portrayed.

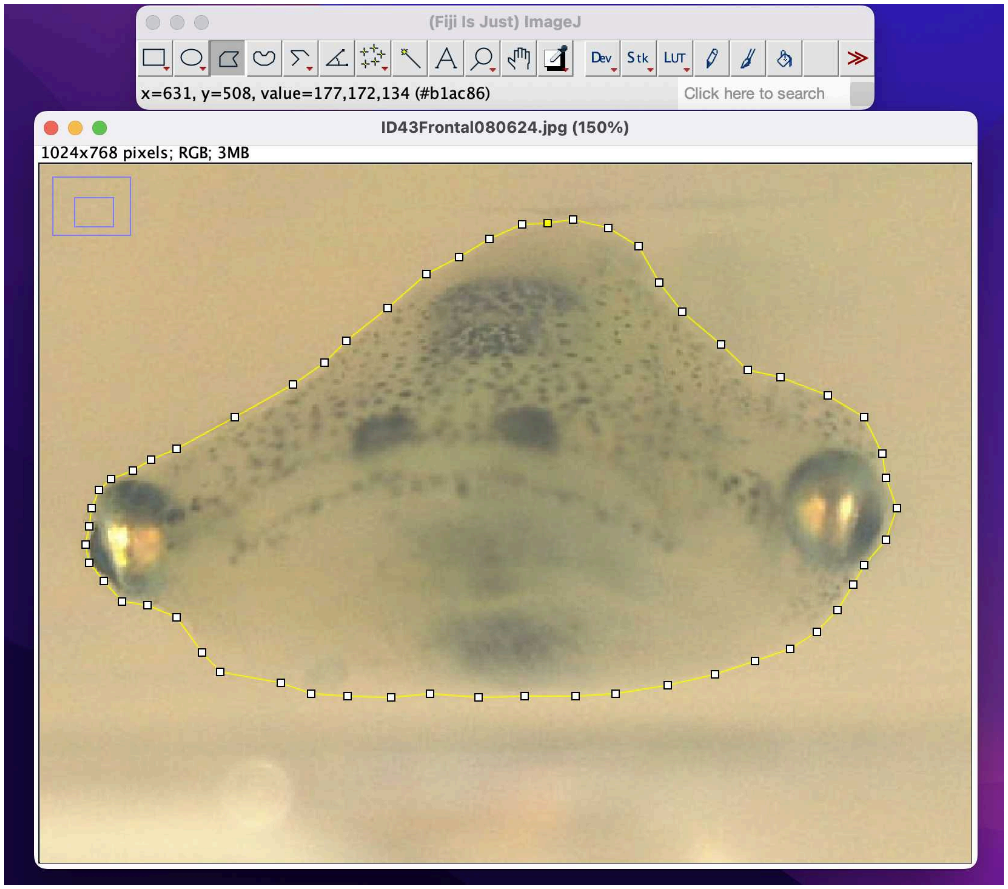

53.9 Click 'command' + 'M' to measure the line. This will give the body area.

54 Repeat steps 49 to 53 for all tadpoles.

Analysis

55 This protocol makes possible morphometric analysis of the anatomical data gathered, along with any other experimental treatments.

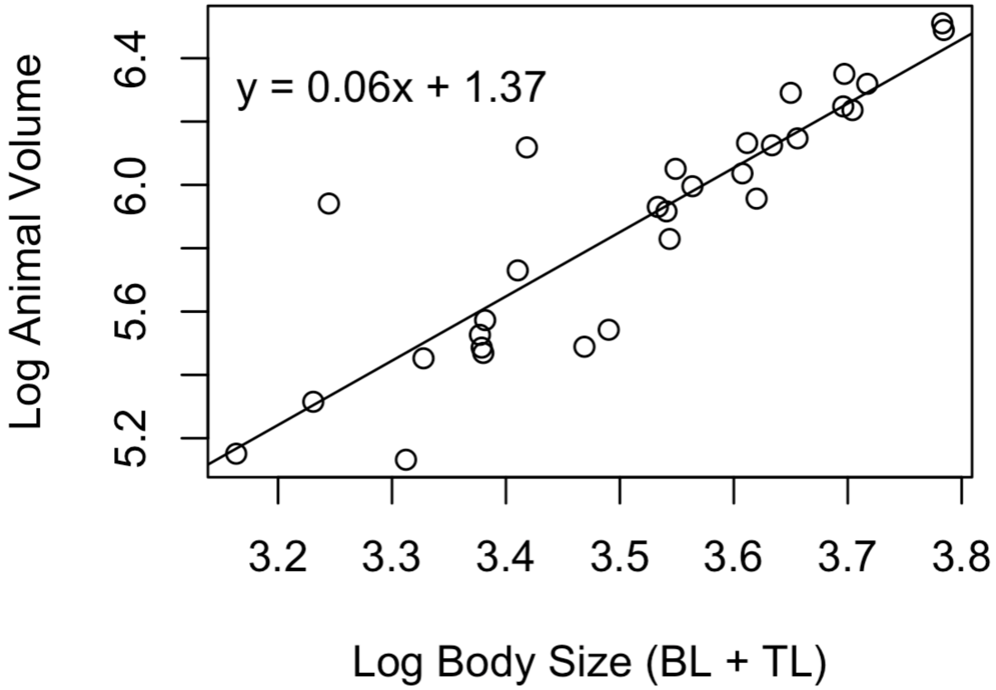

The estimated allometric relationship between (the natural logs of) body volume and body size for N=30 samples. We estimated animal volume as the sum of body and tail volumes. Body volume is the product of dorsal body length, dorsal body width, and lateral body height. Tail volume is the product of dorsal tail length, dorsal tail width, and lateral tail height. Body size is the sum of body length and tail length.

Protocol references

Preibisch, S., Saalfeld, S., & Tomancak, P. (2009). Globally optimal stitching of tiled 3D microscopic image acquisitions. *Bioinformatics*, *25*(11), 1463-1465.

Schindelin, J., Arganda-Carreras, I., Frise, E., Kaynig, V., Longair, M., Pietzsch, T., ... & Cardona, A. (2012). Fiji: an open-source platform for biological-image analysis. *Nature methods*, *9*(7), 676-682.

Acknowledgements

We thank Katie Fiocca, Cesar Mena, and David Ramirez for advice on improving and implementing our protocols.
