# Supplementary material for: Non-lethal imaging and modeling approaches for estimating dry mass in aquatic larvae
Source: PLoS One. 2026 Apr 17;21(4):e0345767. doi: 10.1371/journal.pone.0345767 (PMC13089712; doi:10.1371/journal.pone.0345767)
Supplement: S1 Text — (PDF) [file pone.0345767.s003.pdf]

S1 Text. ANOVA tables for model fits and hyperparameter search results

Non-lethal imaging and modeling approaches for estimating dry mass in aquatic larvae

Short Title: Estimating dry mass in aquatic larvae

Authors: Daniela Granados Frias[1], Najva Akbari[1], Lauren A. O'Connell[1], Bryan H. Juarez[1,2,*]

[1]Department of Biology, Stanford University, Stanford, CA 94305, USA

[2]Earth System Science Department, Stanford University, Stanford, CA 94305, USA

***Corresponding Author Email:** bryanhjuarez@gmail.com

This supplementary material serves to document the ANOVA tables for the eight models fit to evaluate predictions of tadpole dry mass. Additionally, we supply results for the hyperparameter search performed as part of the neural network model (8).

Table A. ANOVA table for Model 1 (wet body mass). Coef is coefficient, DF is degrees of freedom, SS is sum of squares, MS is mean square, *F* is the *F* statistic, and *p* is the *p*-value. Rows with significant *p*-values are in bold. Wet mass was natural log transformed. The intercept for the model is -60.551.

Variable	Coef	DF	SS	MS	*F*	*p*
**Wet mass**	**35.278**	**1**	**12.282**	**12.282**	**89.342**	**<0.001**
Residuals	-	59	8.111	0.137	-	-

Table B. ANOVA table for Model 2 (length). Coef is coefficient, DF is degrees of freedom, SS is sum of squares, MS is mean square, *F* is the *F* statistic, and *p* is the *p*-value. Rows with significant p-values are in bold. DTW is the dorsal tail width, LBH is the lateral body height, LTH is the lateral tail height, LTL is the lateral tail length, FBW is the lateral body width, LLBA is the lateral limb bud area. All variables were natural log transformed, except the LLBA which was square root transformed (see text). The intercept for the model is -10.436.

Variable	Coef	DF	SS	MS	*F*	*p*
**DTW**	**0.241**	**1**	**11.150**	**11.150**	**122.775**	**<0.001**

**LBH**	**0.751**	**1**	**2.810**	**2.810**	**30.942**	**<0.001**
**LTH**	**1.309**	**1**	**1.232**	**1.232**	**13.564**	**0.001**
LTL	0.334	1	0.104	0.104	1.147	0.289
FBW	1.039	1	0.171	0.171	1.878	0.176
LLBA	-0.084	1	0.021	0.021	0.231	0.633
Residuals	-	54	4.904	0.091	-	-

Table C. ANOVA table for Model 3 (surface area). Coef is coefficient, DF is degrees of freedom, SS is sum of squares, MS is mean square, *F* is the *F* statistic, and *p* is the *p*-value. Rows with significant p-values are in bold. DTA is the dorsal tail area, LFA is the lateral fin area, LBA is the lateral body area, LLBA is the lateral limb bud area. All variables were natural log transformed, except the LLBA which was square root transformed (see text). The intercept for the model is -10.201.

Variable	Coef	DF	SS	MS	*F*	*p*
**DTA**	**0.639**	**1**	**13.005**	**13.005**	**118.955**	**<0.001**
**LFA**	**0.401**	**1**	**0.878**	**0.878**	**8.030**	**0.006**
LBA	0.638	1	0.343	0.343	3.139	0.082
LLBA	0.110	1	0.043	0.043	0.395	0.533
Residuals	-	56	6.123	0.109	-	-

Table D. ANOVA table for Model 4 (volumes). Coef is coefficient, DF is degrees of freedom, SS is sum of squares, MS is mean square, *F* is the *F* statistic, and *p* is the *p*-value. Rows with significant p-values are in bold. BV3 is body volume 3 and TMV is tail muscle volume; see table 1. All variables were natural log transformed. The intercept for the model is -10.303.

Variable	Coef	DF	SS	MS	*F*	*p*
**BV3**	**0.639**	**1**	**14.308**	**14.308**	**155.779**	**<0.001**
**TMV**	**0.552**	**1**	**0.757**	**0.757**	**8.237**	**0.006**
Residuals	-	58	5.327	0.092	-	-

Table E. ANOVA table for Model 5 (volumes). Coef is coefficient, DF is degrees of freedom, SS is sum of squares, MS is mean square, *F* is the *F* statistic, and *p* is the *p*-value. Rows with significant p-values are in bold. BV is body volume and TV is tail volume; see table 1. All variables were natural log transformed. The intercept for the model is -11.155.

Variable	Coef	DF	SS	MS	*F*	*p*
**BV5**	**0.717**	**1**	**14.355**	**14.355**	**161.940**	**<0.001**
**TV3**	**0.483**	**1**	**0.896**	**0.896**	**10.111**	**0.002**
Residuals	-	58	5.141	0.089	-	-

Table F. Variable importance table for Model 6 (random forest). Importance is the average decrease in mean square error after splitting on the variable. See Table 1 for more information on variable names.

Variable	Importance
Lateral body area	1.231
Lateral tail area	1.065
Body volume 1	1.048
Tail volume 1	1.010
Body volume 2	0.985
Body volume 3	0.916
Lateral body height	0.830
Body volume 5	0.843
Lateral tail height	0.830
Lateral fin area	0.830
Wet mass	0.784
Frontal body width	0.784
Body volume 4	0.776

Tail muscle volume	0.762
Tail volume 3	0.759
Tail volume 2	0.700
Lateral tail muscle area	0.687
Dorsal body length	0.678
Dorsal body area	0.674
Frontal body area	0.656
Dorsal body width	0.639
Dorsal tail area	0.571
Lateral tail length	0.534
Dorsal tail width	0.400
Dorsal tail length	0.326
Lateral limb bud area	0.276

Table G. Coefficient table for Model 7A and 7B (adaptive lasso). λ is the regularization parameter and 1se is one standard deviation. The minimum *λ* gives the sparsest model that optimizes the MSE and the *λ* 1se coefficients give the sparsest model within one standard error of the minimum. All variables were natural log transformed.

Variable	Coefficient (Minimum *λ*)	Coefficient (*λ* 1se)
Intercept	-15.401	-24.013
Wet mass	3.687	9.665
Dorsal body length	0.444	0.287
Dorsal tail width	0.021	–
Lateral body height	0.461	0.237

Lateral tail height	1.287	1.047
Frontal body width	0.788	0.847

Table H. Hyperparameter search results. Replicate is the replicate run given each combination of Layers and Dense. Layers is the number of neuron layers. Dense is the number of neurons per layer. MSE is the mean square error. MAE is the mean absolute error.

Replicate	Layers	Dense	MSE	MAE	r^2
1	25	2500	0.1050479859	0.276404053	0.05379134417
1	24	2500	0.05105863139	0.1811989248	0.5400947332
1	25	2400	0.08188082278	0.2476375103	0.2624671459
1	24	2400	0.09038837999	0.2530264854	0.1858361959
1	25	2300	0.07324115932	0.2245099545	0.3402879834
1	24	2300	0.1086102277	0.2875313759	0.02170485258
1	25	2200	0.09835572541	0.2570064664	0.1140711904
1	24	2200	0.06159066036	0.2045735866	0.4452286363
1	25	2100	0.1110704914	0.2918871939	-0.000455737114
1	24	2100	0.1110259891	0.2918863297	-5.48E-05
1	25	2000	0.1116821393	0.2918872833	-0.00596511364
1	24	2000	0.1118241176	0.2918859124	-0.007243990898
1	25	1900	0.0639006421	0.1849869192	0.4244217277
1	24	1900	0.1112547368	0.2918848097	-0.002115368843
1	25	1800	0.1113199219	0.2918861508	-0.002702474594
1	24	1800	0.1110462397	0.2918871045	-0.0002373456955
1	25	1700	0.1111041754	0.2918860018	-0.0007591247559
1	24	1700	0.09795633703	0.2742422223	0.1176686287
1	25	1600	0.1061945409	0.282990396	0.04346388578
1	24	1600	0.06018518656	0.2166311294	0.4578883052
1	25	1500	0.08571963012	0.2581447661	0.2278894782
1	24	1500	0.04870004207	0.1937152892	0.5613394976

1	25	1400	0.1110415086	0.2918872237	-0.0001946687698
1	24	1400	0.1110534668	0.2918872237	-0.0003023147583
1	25	1300	0.07101538777	0.2038165033	0.3603364229
1	24	1300	0.1111741513	0.291829735	-0.001389503479
1	25	1200	0.08517681062	0.2563197613	0.2327788472
1	24	1200	0.06246951222	0.2154851407	0.4373124838
1	25	1100	0.06125884131	0.2149774134	0.4482174516
1	24	1100	0.1159666032	0.2918846607	-0.04455697536
1	25	1000	0.04584699124	0.1826306283	0.5870380402
1	24	1000	0.1122440845	0.2918822765	-0.01102674007
1	25	900	0.09790249169	0.2676408291	0.1181536913
1	24	900	0.1110835522	0.2915457785	-0.0005733966827
1	25	800	0.08700911701	0.2589889467	0.2162745595
1	24	800	0.112063624	0.2915497422	-0.009401202202
1	25	700	0.05864118785	0.2170854509	0.4717956781
1	24	700	0.04448094219	0.1898010224	0.5993425846
1	25	600	0.03784148023	0.1688298732	0.6591468453
1	24	600	0.03193143755	0.1638135463	0.7123809457
1	25	500	0.05811721087	0.2087928802	0.4765153527
1	24	500	0.0597111173	0.1802360564	0.4621584415
1	25	400	0.04148078337	0.1774716824	0.6263662577
1	24	400	0.03321680427	0.1526088268	0.7008031607
1	25	300	0.03557477146	0.1656615734	0.6795639992
1	24	300	0.03895042464	0.1729874164	0.6491581202
1	25	200	0.02731432952	0.1464003623	0.7539690733
1	24	200	0.04893632978	0.1837117672	0.5592111349
1	25	120	0.03728257492	0.1770298481	0.6641811132
1	24	120	0.07015493512	0.2129128426	0.3680868149
1	25	60	0.07909999788	0.2366323024	0.2875151634
1	24	60	0.1619143933	0.3361591399	-0.4584268332

1	25	30	0.1733559817	0.3518542647	-0.5614857674
1	24	30	0.04385183379	0.150991872	0.6050091982
1	23	2500	0.07216536999	0.2232542038	0.3499780297
1	22	2500	0.111043334	0.2918864787	-0.0002111196518
1	21	2500	0.0489648059	0.1923407614	0.5589546561
1	23	2400	0.05712645128	0.2107926905	0.485439539
1	22	2400	0.1122549996	0.2918871045	-0.01112508774
1	21	2400	0.06163306907	0.1835646182	0.4448466301
1	23	2300	0.111221537	0.291885525	-0.001816272736
1	22	2300	0.08297137916	0.2498544157	0.252644062
1	21	2300	0.1100618094	0.2873412967	0.008629858494
1	23	2200	0.1015322208	0.275277555	0.08545923233
1	22	2200	0.1110597625	0.2918872833	-0.0003590583801
1	21	2200	0.05736516789	0.2010453194	0.4832893014
1	23	2100	0.07577905804	0.224540323	0.3174281716
1	22	2100	0.07763101161	0.2199014127	0.3007469177
1	21	2100	0.111130096	0.2918857932	-0.0009925365448
1	23	2000	0.1074004024	0.2877082229	0.03260219097
1	22	2000	0.09482036531	0.2266878635	0.1459155679
1	21	2000	0.05032082647	0.1812252998	0.5467404127
1	23	1900	0.0851790756	0.248902753	0.2327584624
1	22	1900	0.1114292368	0.2918872833	-0.003687143326
1	21	1900	0.06184050441	0.2064164132	0.4429782033
1	23	1800	0.1114007011	0.2918843329	-0.003430008888
1	22	1800	0.07727244496	0.2380700558	0.3039765954
1	21	1800	0.05800552294	0.18328242	0.4775213599
1	23	1700	0.111034289	0.2918871045	-0.0001295804977
1	22	1700	0.05263628438	0.1975309849	0.5258842707

1	21	1700	0.05385760218	0.1981663257	0.5148833394
1	23	1600	0.05293899775	0.1984700263	0.5231575966
1	22	1600	0.09618767351	0.2618485987	0.1335996985
1	21	1600	0.1110348701	0.2918747067	-0.0001348257065
1	23	1500	0.05356631801	0.1885941476	0.5175070763
1	22	1500	0.06403020769	0.2038989961	0.4232546687
1	21	1500	0.05393237993	0.1875329018	0.5142097473
1	23	1400	0.06241177395	0.1909599751	0.4378325343
1	22	1400	0.08727669716	0.2531901002	0.2138643861
1	21	1400	0.05520723388	0.2058908492	0.5027266741
1	23	1300	0.06322082877	0.2287543714	0.4305450916
1	22	1300	0.06279724836	0.207873255	0.4343604445
1	21	1300	0.05096087605	0.2016243935	0.5409753323
1	23	1200	0.04889903218	0.1876554489	0.5595471263
1	22	1200	0.1111832112	0.2918794155	-0.001471042633
1	21	1200	0.05065286905	0.198216483	0.5437496901
1	23	1100	0.04684506357	0.183114484	0.5780480504
1	22	1100	0.07684396207	0.242487818	0.3078361154
1	21	1100	0.04958700389	0.195363611	0.5533503294
1	23	1000	0.05535895377	0.18887797	0.5013600588
1	22	1000	0.0519656837	0.1993834078	0.5319246054
1	21	1000	0.05968914554	0.1989078969	0.462356329
1	23	900	0.0592366159	0.2062331736	0.4664324522
1	22	900	0.0490684621	0.2037837058	0.558021009
1	21	900	0.05308138579	0.1912384927	0.5218750238
1	23	800	0.04815066978	0.1742839813	0.5662879348
1	22	800	0.1079354659	0.2827262878	0.0277826786
1	21	800	0.03065425158	0.1360584795	0.7238850594
1	23	700	0.06927368045	0.2103685439	0.3760246634
1	22	700	0.06042525917	0.2245647013	0.4557258487
1	21	700	0.04991044104	0.1976319253	0.5504369736
1	23	600	0.07743597031	0.2367427796	0.302503705
1	22	600	0.05062708259	0.1941881627	0.5439819098

1	21	600	0.06415463984	0.1989109069	0.422133863
1	23	500	0.2158429921	0.3998450339	-0.9441828728
1	22	500	0.04161138833	0.1745065153	0.6251897812
1	21	500	0.03942899778	0.1805447042	0.6448475122
1	23	400	0.04264159873	0.1785395592	0.6159102917
1	22	400	0.02683269978	0.1319669783	0.7583072782
1	21	400	0.04755732417	0.1765086651	0.5716323853
1	23	300	0.04084904492	0.1495029926	0.6320565343
1	22	300	0.05809498951	0.1966760159	0.4767155051
1	21	300	0.05356506631	0.1747266352	0.5175182819
1	23	200	0.04690700024	0.1867646724	0.5774901509
1	22	200	0.1600936204	0.3618484437	-0.4420263767
1	21	200	0.04927467555	0.2029353678	0.5561635494
1	23	120	0.05447316915	0.1907355338	0.5093387365
1	22	120	0.07427476346	0.2256399095	0.3309778571
1	21	120	0.06330359727	0.2214565724	0.4297995567
1	23	60	0.05077562481	0.1951525211	0.5426439047
1	22	60	0.08692535013	0.2439321578	0.2170290947
1	21	60	0.1605079919	0.3367913961	-0.4457587004
1	23	30	0.1805832684	0.3475268483	-0.6265847683
1	22	30	0.2519858778	0.3968514502	-1.26973629
1	21	30	0.1644021124	0.3537375033	-0.4808347225
1	20	2500	0.0847152397	0.2562821805	0.2369363904
1	19	2500	0.06543410569	0.2215441763	0.4106092453
1	18	2500	0.04276100546	0.1763596535	0.6148347855
1	17	2500	0.1017239466	0.2796545029	0.08373230696
1	16	2500	0.04669002816	0.1820251942	0.5794445276
1	20	2400	0.0549473539	0.2149893343	0.5050675273
1	19	2400	0.08743433654	0.260571301	0.2124444246
1	18	2400	0.06370834261	0.2349280864	0.4261538386
1	17	2400	0.05128703639	0.2045444548	0.5380374193
1	16	2400	0.05780149624	0.2052056342	0.4793591499
1	20	2300	0.0556628108	0.1947637498	0.4986231327
1	19	2300	0.1107812673	0.2914524674	0.0021494627
1	18	2300	0.04040636495	0.174394995	0.6360439062

1	17	2300	0.06266903132	0.2130855024	0.4355152845
1	16	2300	0.04796377569	0.1763458699	0.5679713488
1	20	2200	0.08170025796	0.2309225053	0.2640935183
1	19	2200	0.05868621543	0.2007000446	0.4713901281
1	18	2200	0.04778825492	0.17852512	0.569552362
1	17	2200	0.05415131524	0.1915568858	0.5122377872
1	16	2200	0.04625754431	0.1751757562	0.5833400488
1	20	2100	0.1296996176	0.2991761565	-0.1682556868
1	19	2100	0.1006520241	0.2721307874	0.09338748455
1	18	2100	0.06254352629	0.2199015617	0.4366457462
1	17	2100	0.05349040776	0.2050072253	0.5181908011
1	16	2100	0.130742237	0.3008320332	-0.1776468754
1	20	2000	0.0593697913	0.1866879463	0.4652329087
1	19	2000	0.08752964437	0.2141618729	0.2115859985
1	18	2000	0.0440325439	0.1847056448	0.6033815145
1	17	2000	0.04898989573	0.1816961765	0.5587286949
1	16	2000	0.1832243651	0.4025993347	-0.6503741741
1	20	1900	0.05124863237	0.1983290166	0.5383833647
1	19	1900	0.06226445362	0.2046820223	0.4391595125
1	18	1900	0.05288159847	0.1933577955	0.5236746073
1	17	1900	0.04755515605	0.1864216328	0.5716519356
1	16	1900	0.04942952842	0.2018158436	0.5547687411
1	20	1800	0.06031662226	0.203271538	0.4567043781
1	19	1800	0.05447684973	0.2012695372	0.5093054771
1	18	1800	0.1111590415	0.29178828	-0.001253247261
1	17	1800	0.05445660278	0.1911893338	0.5094878674
1	16	1800	0.02312210202	0.1224199757	0.7917301059
1	20	1700	0.06115233153	0.2286365032	0.4491768479
1	19	1700	0.06883743405	0.2209536135	0.3799540997
1	18	1700	0.07328383625	0.2272786647	0.3399035931
1	17	1700	0.06184850261	0.2148864716	0.4429061413
1	16	1700	0.05414770171	0.2039284259	0.5122703314
1	20	1600	0.07017678767	0.2284487784	0.3678900003
1	19	1600	0.06709638238	0.2059112489	0.3956364393

1	18	1600	0.07819230855	0.2502501905	0.2956910133
1	17	1600	0.05027722567	0.1980626136	0.5471332073
1	16	1600	0.05318040773	0.2035206854	0.5209830999
1	20	1500	0.07966437191	0.2045556605	0.2824315429
1	19	1500	0.04999446124	0.2024774998	0.5496801734
1	18	1500	0.05625620484	0.2093821466	0.4932782054
1	17	1500	0.05442658812	0.1925926656	0.509758234
1	16	1500	0.04418480396	0.1716001034	0.6020100117
1	20	1400	0.07519581169	0.25339064	0.3226816654
1	19	1400	0.05624029785	0.2089475691	0.493421495
1	18	1400	0.06210398674	0.219955489	0.4406049252
1	17	1400	0.05401607603	0.1994266957	0.5134558678
1	16	1400	0.05439745262	0.20652242	0.5100207329
1	20	1300	0.0605487451	0.1914285123	0.4546135664
1	19	1300	0.0577741079	0.2040175498	0.479605794
1	18	1300	0.05182734877	0.19794707	0.5331706405
1	17	1300	0.03738829121	0.1607443839	0.6632288694
1	16	1300	0.03294489533	0.1453474015	0.7032523155
1	20	1200	0.07405900955	0.2390948832	0.3329212666
1	19	1200	0.05968154222	0.2208092213	0.4624248147
1	18	1200	0.04639979824	0.1867194623	0.5820586681
1	17	1200	0.04520409927	0.1768798828	0.5928288698
1	16	1200	0.04835162684	0.1850871593	0.5644778013
1	20	1100	0.046300482	0.1889761388	0.5829533339
1	19	1100	0.06677286327	0.218664974	0.3985504508
1	18	1100	0.04723698646	0.1712614596	0.5745178461
1	17	1100	0.04798309132	0.1952822655	0.5677973628
1	16	1100	0.04702219367	0.1873207539	0.5764525533
1	20	1000	0.07547681779	0.192909047	0.3201504946
1	19	1000	0.086103715	0.2181117088	0.2244299054
1	18	1000	0.0358408615	0.1572875082	0.6771672368
1	17	1000	0.04339193925	0.172581479	0.609151721
1	16	1000	0.02842115425	0.1538472176	0.7439994812
1	20	900	0.09585695714	0.2848543525	0.1365785599
1	19	900	0.05973274633	0.2011689693	0.461963594

1	18	900	0.05689587444	0.1978377849	0.4875164032
1	17	900	0.02927240171	0.1274541914	0.7363319397
1	16	900	0.03003497422	0.1394298971	0.7294631004
1	20	800	0.04536437243	0.1914586127	0.5913851857
1	19	800	0.08434845507	0.2687434554	0.2402402163
1	18	800	0.0354905799	0.1528042853	0.6803223491
1	17	800	0.03970588744	0.1667540073	0.6423534155
1	16	800	0.03932302445	0.169717595	0.645802021
1	20	700	0.03389905766	0.1463094205	0.6946578026
1	19	700	0.03038643859	0.1567155421	0.7262973785
1	18	700	0.06323614717	0.199262768	0.4304071069
1	17	700	0.05675011128	0.1849993169	0.4888293743
1	16	700	0.1855427921	0.3928056359	-0.671257019
1	20	600	0.05808219314	0.2117530406	0.4768307805
1	19	600	0.02955342457	0.1375614703	0.7338006496
1	18	600	0.04017022997	0.1567471474	0.638170898
1	17	600	0.05984013155	0.182329461	0.4609963298
1	16	600	0.03852198645	0.1638996601	0.6530172825
1	20	500	0.1289515048	0.3276751041	-0.161517024
1	19	500	0.1149561554	0.3127580583	-0.0354553461<br>1
1	18	500	0.1178234369	0.3007174432	-0.0612821579
1	17	500	0.2853138447	0.4362095892	-1.569934368
1	16	500	0.2500571907	0.4475750923	-1.252363682
1	20	400	0.04662609845	0.1940259933	0.5800203681
1	19	400	0.1281742454	0.2940762043	-0.1545159817
1	18	400	0.0389460735	0.1657795012	0.64919734
1	17	400	0.3747172356	0.5284980536	-2.375226021
1	16	400	0.04130239412	0.1629261076	0.6279730797
1	20	300	0.1439164281	0.3400036693	-0.2963119745
1	19	300	0.08441780508	0.2329470217	0.2396155596
1	18	300	0.1126642227	0.3064614236	-0.0148110389<br>7
1	17	300	0.3647288382	0.508125186	-2.285256386
1	16	300	0.06683896482	0.2334084958	0.39795506

1	20	200	0.1450648308	0.3411764503	-0.3066561222
1	19	200	0.3229042292	0.5101057291	-1.908525705
1	18	200	0.1036546379	0.2850475311	0.06634181738
1	17	200	0.1119992882	0.2959724367	-0.008821725845
1	16	200	0.1836504638	0.385661453	-0.6542122364
1	20	120	0.1266174316	0.3305025995	-0.1404931545
1	19	120	0.137013033	0.3090812862	-0.2341303825
1	18	120	0.1647933424	0.3321406245	-0.4843586683
1	17	120	0.1015004963	0.2651575208	0.085744977
1	16	120	0.1326449811	0.2922247648	-0.1947857141
1	20	60	0.1919145137	0.370054692	-0.7286497355
1	19	60	0.1718409508	0.3565496802	-0.5478392839
1	18	60	0.1750671417	0.357851088	-0.5768988132
1	17	60	0.07010753453	0.2321081161	0.3685138226
1	16	60	0.1559556276	0.3628018796	-0.4047538042
1	20	30	0.1124085039	0.282966435	-0.01250779629
1	19	30	0.3376783729	0.4965181947	-2.041602135
1	18	30	0.7205979228	0.7588707209	-5.490709782
1	17	30	0.4431816936	0.5495370626	-2.991912365
1	16	30	0.2879812717	0.4869731963	-1.593961
1	15	2500	0.04732123017	0.1957713068	0.5737590194
1	14	2500	0.570014596	0.6516364813	-4.134346008
1	13	2500	0.06099639088	0.2105743438	0.450581491
1	12	2500	0.0364597626	0.1582065523	0.6715925336
1	11	2500	0.0590229705	0.2240590155	0.4683568478
1	15	2400	0.05400567129	0.2077038735	0.5135496259
1	14	2400	0.05735886097	0.2005571872	0.4833461046
1	13	2400	0.04696432501	0.1892781705	0.5769737959
1	12	2400	0.04780405015	0.1813232452	0.5694100857
1	11	2400	0.04205774516	0.1864831001	0.6211693287
1	15	2300	0.05348423868	0.2084723413	0.5182464123
1	14	2300	0.05074778944	0.1961366236	0.5428946614
1	13	2300	0.03425068408	0.1494475305	0.6914905906

1	12	2300	0.0463411659	0.1849428713	0.5825868845
1	11	2300	0.04429822043	0.1801960915	0.6009885073
1	15	2200	0.06338714063	0.2195665389	0.4290469885
1	14	2200	0.05394716188	0.187940076	0.5140765905
1	13	2200	0.0312675871	0.1461604089	0.7183605433
1	12	2200	0.1481145918	0.2882616222	-0.3341265917
1	11	2200	0.09149651229	0.2806414962	0.1758548617
1	15	2100	0.05405181646	0.1981663704	0.5131340027
1	14	2100	0.9865299463	0.83434093	-7.886064529
1	13	2100	0.03999041393	0.1502564847	0.639790535
1	12	2100	0.4261175096	0.5786269903	-2.838208437
1	11	2100	0.02945344523	0.1396948844	0.7347012162
1	15	2000	0.3544658124	0.5420523882	-2.192813396
1	14	2000	0.05559812114	0.2101980746	0.4992057681
1	13	2000	0.03379981965	0.1454081088	0.6955516934
1	12	2000	0.04283482209	0.1819378436	0.614169836
1	11	2000	0.06269963086	0.2057002485	0.4352397323
1	15	1900	0.03051517345	0.134749651	0.7251378298
1	14	1900	0.05256352946	0.1740167588	0.5265395641
1	13	1900	0.04403159767	0.1846576184	0.603390038
1	12	1900	0.1503356993	0.3056359887	-0.3541328907
1	11	1900	0.03165536374	0.1310002804	0.7148676515
1	15	1800	0.0500763841	0.1903148592	0.5489422679
1	14	1800	0.0346972011	0.1491860896	0.687468648
1	13	1800	0.04141562432	0.1770093441	0.626953125
1	12	1800	0.3352075815	0.5046866536	-2.019346952
1	11	1800	0.07302514464	0.2461469173	0.3422337174
1	15	1700	0.0513783209	0.1984931976	0.5372152328
1	14	1700	0.05397773907	0.1978508532	0.5138012171
1	13	1700	0.05529871583	0.1891676486	0.5019026995
1	12	1700	0.0314441137	0.151039511	0.7167704701
1	11	1700	0.387652576	0.5478368998	-2.49173975
1	15	1600	0.04978252202	0.1789435446	0.551589191
1	14	1600	0.04129159823	0.1688881814	0.6280702949
1	13	1600	0.0304683242	0.1324298382	0.7255598307

1	12	1600	0.04258292168	0.1725250781	0.6164388061
1	11	1600	0.461347878	0.52898103	-3.155542374
1	15	1500	0.033432208	0.1584390104	0.6988629103
1	14	1500	0.07253644615	0.222570464	0.3466356397
1	13	1500	0.134899348	0.3256402314	-0.2150917053
1	12	1500	0.04720432684	0.1770672798	0.574811995
1	11	1500	0.03904566914	0.1711103022	0.6483002901
1	15	1400	0.03928196058	0.165150404	0.6461718678
1	14	1400	0.04584454745	0.1498954743	0.5870600939
1	13	1400	0.3868625164	0.560711205	-2.484623194
1	12	1400	0.06931435317	0.2379470766	0.3756582737
1	11	1400	0.06734511256	0.2140328884	0.3933960199
1	15	1300	0.05060943961	0.2011705339	0.5441408157
1	14	1300	0.02846534923	0.1362323761	0.7436013818
1	13	1300	0.2895100713	0.4710600376	-1.607731342
1	12	1300	0.03115810081	0.1355374306	0.7193467021
1	11	1300	0.2510817051	0.4315856099	-1.261591911
1	15	1200	0.04300976545	0.1664431989	0.6125941277
1	14	1200	0.03621502221	0.1459103525	0.6737970114
1	13	1200	0.07547669113	0.2528301179	0.3201516867
1	12	1200	0.05537767336	0.1936689317	0.5011914372
1	11	1200	0.04486035556	0.1720056981	0.5959250927
1	15	1100	0.03867840022	0.1769123077	0.6516084075
1	14	1100	0.04231775925	0.1799221039	0.6188272238
1	13	1100	0.02814676799	0.1286626756	0.7464709282
1	12	1100	0.03617428988	0.1668896675	0.674163878
1	11	1100	0.1541607976	0.3522985578	-0.3885871172
1	15	1000	0.04247071221	0.1692239344	0.617449522
1	14	1000	0.02820653841	0.1274963915	0.745932579
1	13	1000	0.36302948	0.5230851173	-2.269949675
1	12	1000	0.1324035972	0.3268436491	-0.1926114559
1	11	1000	0.2460546196	0.4274764061	-1.216310978
1	15	900	0.02622305974	0.1196177006	0.7637985349
1	14	900	0.0473032929	0.1792618334	0.5739206076
1	13	900	0.05069407076	0.1911573857	0.5433784723

1	12	900	0.06466398388	0.234085083	0.4175460339
1	11	900	0.07202323526	0.2338404655	0.3512582779
1	15	800	0.1852559149	0.3994701505	-0.6686730385
1	14	800	0.03503758088	0.1472030133	0.6844027042
1	13	800	0.06349183619	0.2233561575	0.428104043
1	12	800	0.2960441709	0.4684242606	-1.666586637
1	11	800	0.5178567767	0.6186767817	-3.664540291
1	15	700	0.02516577207	0.1423768103	0.7733219862
1	14	700	0.08225087076	0.2716069221	0.2591339946
1	13	700	0.1337201297	0.3344936967	-0.2044700384
1	12	700	0.2101603448	0.4286956191	-0.8929971457
1	11	700	0.4031125009	0.5533917546	-2.630993366
1	15	600	0.06210104376	0.2102908641	0.4406314492
1	14	600	0.3159123063	0.4995997548	-1.845546722
1	13	600	0.1442072839	0.3581988811	-0.2989319563
1	12	600	0.2532204986	0.4580750465	-1.280857086
1	11	600	0.2770306468	0.4683925509	-1.495324373
1	15	500	0.2218291759	0.4244596362	-0.9981029034
1	14	500	0.4280846715	0.5530819893	-2.855927467
1	13	500	0.2742769718	0.4703775346	-1.470520735
1	12	500	0.1675563902	0.3216418326	-0.5092464685
1	11	500	0.3576648831	0.5278441906	-2.221628666
1	15	400	0.1483162344	0.3486820161	-0.3359428644
1	14	400	0.1801352799	0.3784507513	-0.6225494146
1	13	400	0.05882219598	0.2234524786	0.4701652527
1	12	400	0.2191688269	0.414463222	-0.974140048
1	11	400	0.3582873344	0.5024736524	-2.227235317
1	15	300	0.1895013154	0.3707527518	-0.7069131136
1	14	300	0.1839360446	0.3808969855	-0.6567845345
1	13	300	0.2460055649	0.4292078912	-1.215869188
1	12	300	0.2719197571	0.4606151581	-1.449288368
1	11	300	0.3140559793	0.4481975138	-1.828825951
1	15	200	0.1522643566	0.3146449625	-0.3715051413
1	14	200	0.1547571123	0.3388321698	-0.3939583302
1	13	200	0.3636860847	0.4838778973	-2.275863886

1	12	200	0.1693876237	0.3642406762	-0.5257411003
1	11	200	0.4281234741	0.5345422029	-2.856276989
1	15	120	0.2745643258	0.4633372426	-1.473109007
1	14	120	0.07943394035	0.256927669	0.2845071554
1	13	120	0.1082488298	0.2893489301	0.02496010065
1	12	120	0.2696302235	0.4349524975	-1.428665638
1	11	120	0.3692054152	0.522145927	-2.325578928
1	15	60	0.1853169054	0.3718132377	-0.6692224741
1	14	60	0.2719115615	0.4812524319	-1.449214697
1	13	60	0.2687041461	0.4037569165	-1.420324326
1	12	60	0.4696958959	0.5735018253	-3.230736256
1	11	60	0.4320183694	0.5819295645	-2.891359806
1	15	30	0.1999073327	0.3629048169	-0.8006442785
1	14	30	0.5832718611	0.6082317233	-4.253759384
1	13	30	0.4257109761	0.5827208757	-2.834546566
1	12	30	0.6906698942	0.7326062322	-5.22113657
1	11	30	0.4650268555	0.4938617349	-3.188680172
1	10	2500	0.1010598987	0.2702259421	0.08971363306
1	9	2500	0.484013468	0.5895616412	-3.359700203
1	8	2500	0.9079252481	0.8201072812	-7.178040504
1	7	2500	0.5475785732	0.6199581027	-3.932256222
1	6	2500	0.6119397879	0.7028720975	-4.511983395
1	10	2400	0.1495500356	0.345754087	-0.3470561504
1	9	2400	0.10410285	0.2961258888	0.06230455637
1	8	2400	0.304412365	0.4572626054	-1.741962194
1	7	2400	0.5885924101	0.6588212252	-4.30168438
1	6	2400	0.5445146561	0.6349844337	-3.904658318
1	10	2300	0.08115824312	0.2282865942	0.2689756751
1	9	2300	0.7592795491	0.7114694715	-5.839130402
1	8	2300	0.6299346685	0.6828837395	-4.674070358
1	7	2300	0.7321885824	0.7522730231	-5.59511137
1	6	2300	0.7973906994	0.7633951306	-6.182412624
1	10	2200	0.04581724852	0.1934915483	0.5873060226
1	9	2200	0.1992497891	0.3978889585	-0.7947214842
1	8	2200	0.3953929245	0.5073716044	-2.561460018

1	7	2200	0.4375546575	0.5567554235	-2.941227198
1	6	2200	0.5818756819	0.6365426779	-4.241183758
1	10	2100	0.1552572548	0.3516046107	-0.3984632492
1	9	2100	0.7251817584	0.7509803176	-5.531998158
1	8	2100	0.5963980556	0.6458201408	-4.371992588
1	7	2100	0.7439990044	0.7502721548	-5.70149231
1	6	2100	0.7711516619	0.749160409	-5.946066856
1	10	2000	0.05886069685	0.2289471626	0.4698185325
1	9	2000	0.5792604685	0.6655985117	-4.217627048
1	8	2000	0.5497390032	0.5770014524	-3.951715946
1	7	2000	0.5097712278	0.6129057407	-3.591710567
1	6	2000	0.5801920295	0.6621972322	-4.226018429
1	10	1900	0.2088950127	0.4261564612	-0.8815997839
1	9	1900	0.727075696	0.709652245	-5.549057484
1	8	1900	0.6233996153	0.693213582	-4.615205765
1	7	1900	0.3150995374	0.4803682268	-1.838225842
1	6	1900	0.6440125704	0.6803523302	-4.800875187
1	10	1800	0.348398596	0.525765121	-2.138163567
1	9	1800	0.4648089409	0.6000355482	-3.18671751
1	8	1800	0.5858706832	0.6223025322	-4.277168274
1	7	1800	0.6977078319	0.7329992056	-5.284529686
1	6	1800	0.6206325293	0.6699656248	-4.590281963
1	10	1700	0.07302457094	0.2207769454	0.342238903
1	9	1700	0.4052985311	0.5449579954	-2.650683641
1	8	1700	0.6361078024	0.6632813215	-4.729673862
1	7	1700	0.3717415333	0.5200868249	-2.348422527
1	6	1700	0.7378989458	0.739050746	-5.646546364
1	10	1600	0.1734813154	0.3809311092	-0.5626146793
1	9	1600	0.2153981477	0.4093378484	-0.9401760101
1	8	1600	0.3618902862	0.5383911133	-2.259688616
1	7	1600	0.5111968517	0.5998097658	-3.604551792
1	6	1600	0.5987696648	0.6627055407	-4.393354416
1	10	1500	0.5604965091	0.6471480131	-4.048613071
1	9	1500	0.1101522222	0.2910269797	0.007815480232

1	8	1500	0.5053120852	0.604321301	-3.551545143
1	7	1500	0.5629141927	0.6069041491	-4.070390224
1	6	1500	0.2635069489	0.468034178	-1.373511076
1	10	1400	0.1532122791	0.3584595621	-0.3800435066
1	9	1400	0.3925278485	0.5075514913	-2.535653114
1	8	1400	0.1741728783	0.3766959012	-0.5688438416
1	7	1400	0.5105332136	0.6137876511	-3.598573685
1	6	1400	0.550470233	0.6479007006	-3.958302498
1	10	1300	0.4127284884	0.5555628538	-2.717608452
1	9	1300	0.1298499107	0.3354506493	-0.1696093082
1	8	1300	0.2506862581	0.451213181	-1.258029938
1	7	1300	0.3147013783	0.4827118814	-1.834639311
1	6	1300	0.654776752	0.6800971031	-4.897832394
1	10	1200	0.2660116255	0.4471670091	-1.396071672
1	9	1200	0.4204034805	0.5581099391	-2.786740065
1	8	1200	0.1688607931	0.3588345945	-0.5209957361
1	7	1200	0.3605754972	0.4869875312	-2.24784565
1	6	1200	0.6342816949	0.6482795477	-4.713225365
1	10	1100	0.05148379132	0.1922225058	0.5362651944
1	9	1100	0.2360471934	0.3901514709	-1.126170158
1	8	1100	0.3794293106	0.5492243767	-2.417669535
1	7	1100	0.5928317308	0.6147329211	-4.339869022
1	6	1100	0.6186646819	0.6440842152	-4.572556973
1	10	1000	0.3952963352	0.5707237124	-2.560590029
1	9	1000	0.3624177873	0.4719552994	-2.26444006
1	8	1000	0.3631783426	0.5002908707	-2.271290541
1	7	1000	0.3147444129	0.4538860321	-1.835027218
1	6	1000	0.7798096538	0.7450600863	-6.024053097
1	10	900	0.2025540769	0.4109710157	-0.8244844675
1	9	900	0.2981296182	0.4472963214	-1.685371161
1	8	900	0.5991128683	0.6324282885	-4.396446228
1	7	900	0.4507285953	0.5505199432	-3.05989027
1	6	900	0.6995566487	0.6668474078	-5.301182747
1	10	800	0.3383350074	0.484877497	-2.047516823
1	9	800	0.5053299665	0.5972170234	-3.551706314

1	8	800	0.3768514693	0.5210895538	-2.394449711
1	7	800	0.3496192098	0.5112271309	-2.149158239
1	6	800	0.6501156688	0.6534786224	-4.855848312
1	10	700	0.2717402577	0.3851033449	-1.447671652
1	9	700	0.362947315	0.4980916381	-2.269209623
1	8	700	0.488920778	0.577907443	-3.403902531
1	7	700	0.4687538147	0.5826446414	-3.222250462
1	6	700	0.8186607361	0.7373560667	-6.374000072
1	10	600	0.6533421874	0.703636229	-4.88491106
1	9	600	0.7672346234	0.7397784591	-5.910784721
1	8	600	0.452688694	0.5894762874	-3.077545643
1	7	600	0.5163265467	0.5819753408	-3.650756836
1	6	600	0.5571786761	0.5960017443	-4.018728256
1	10	500	0.5181623697	0.5963699818	-3.667292595
1	9	500	0.30059129	0.4662080705	-1.707544088
1	8	500	0.4109795094	0.5306138992	-2.701854467
1	7	500	0.5869776607	0.6372232437	-4.287139416
1	6	500	0.4890604615	0.556938827	-3.405160427
1	10	400	0.2472949922	0.3983023763	-1.227483511
1	9	400	0.3382910192	0.4943981767	-2.047120571
1	8	400	0.5256053805	0.6247957945	-3.734334946
1	7	400	0.658554256	0.6617445946	-4.931858063
1	6	400	0.6369010806	0.6277540922	-4.736819267
1	10	300	0.3523924351	0.5174297094	-2.174137592
1	9	300	0.3053899407	0.4748397768	-1.750767469
1	8	300	0.4925465584	0.5787546039	-3.436561108
1	7	300	0.4603794515	0.5493642092	-3.146819115
1	6	300	0.5833098292	0.6091710925	-4.254101753
1	10	200	0.3059907556	0.4774124026	-1.756179333
1	9	200	0.2903018892	0.4135403633	-1.614863634
1	8	200	0.5425392389	0.6344278455	-3.886864662
1	7	200	0.3289633691	0.4797507823	-1.963102818
1	6	200	0.58481282	0.6266815066	-4.267639637
1	10	120	0.6016222835	0.685282886	-4.419049263
1	9	120	0.5417732	0.6406642795	-3.879965305

1	8	120	0.4251858592	0.5467220545	-2.829816818
1	7	120	0.3382975161	0.4511137009	-2.047178984
1	6	120	0.3033386171	0.4957548082	-1.732290506
1	10	60	0.2588640749	0.4762846529	-1.331690788
1	9	60	0.3790925145	0.536215663	-2.414635658
1	8	60	0.3462104201	0.5045265555	-2.118453741
1	7	60	0.5717070103	0.5724875331	-4.149590492
1	6	60	0.2813553214	0.4448298514	-1.534278154
1	10	30	0.9755450487	0.9292967916	-7.787118912
1	9	30	1.803253412	1.273212433	-15.24261475
1	8	30	1.214118958	0.9785524607	-9.936048508
1	7	30	0.5975111723	0.6302709579	-4.382019043
1	6	30	1.063141346	0.9562388659	-8.576133728
1	5	2500	0.7046343088	0.7072554827	-5.346919537
1	4	2500	0.7053962946	0.6906601191	-5.353782654
1	3	2500	0.9560673833	0.7959655523	-7.611676216
1	2	2500	0.5199311972	0.5725960732	-3.683225632
1	1	2500	0.7222194672	0.7118577957	-5.505315781
1	5	2400	1.042165875	0.8462377787	-8.387198448
1	4	2400	0.6742998362	0.669888854	-5.073684216
1	3	2400	0.6623319983	0.6948984861	-4.965885639
1	2	2400	0.9476755261	0.7861174345	-7.536087036
1	1	2400	0.795694828	0.7233170271	-6.167137146
1	5	2300	0.9459007978	0.840868175	-7.520101547
1	4	2300	0.7145411372	0.7119832039	-5.436153889
1	3	2300	0.7917197943	0.7486959696	-6.131332874
1	2	2300	0.7812181115	0.7233858705	-6.036739349
1	1	2300	0.8962076306	0.8028792143	-7.072495461
1	5	2200	0.7103925943	0.7146446705	-5.398786068
1	4	2200	0.8397760391	0.7142770886	-6.564194202
1	3	2200	1.0357517	0.8436347842	-8.329423904
1	2	2200	0.6988582611	0.6872650385	-5.294891834
1	1	2200	0.9121991992	0.822444737	-7.216538429
1	5	2100	0.6605862379	0.6376036406	-4.95016098
1	4	2100	0.7869368792	0.7424381971	-6.088251114

1	3	2100	0.6823910475	0.6778534651	-5.146565437
1	2	2100	0.7540588975	0.6760619283	-5.792105675
1	1	2100	0.8735660315	0.7462683916	-6.868554115
1	5	2000	0.7499070764	0.7174172401	-5.754708767
1	4	2000	0.5218508244	0.5891118646	-3.700516224
1	3	2000	0.751568675	0.6563212872	-5.769675732
1	2	2000	0.6465662718	0.6753513217	-4.823877335
1	1	2000	0.953101337	0.80948174	-7.584959984
1	5	1900	0.5371534228	0.5814309716	-3.83835268
1	4	1900	0.8598869443	0.7631812096	-6.745340824
1	3	1900	0.9328004718	0.8215726614	-7.402101517
1	2	1900	0.7970756292	0.7318607569	-6.179574966
1	1	1900	1.137225389	0.8956903219	-9.243436813
1	5	1800	0.5988602638	0.6453590989	-4.394170761
1	4	1800	0.9023803473	0.7577720881	-7.128095627
1	3	1800	0.5362256169	0.6280695796	-3.829995632
1	2	1800	0.6894600391	0.6704691052	-5.210238457
1	1	1800	1.186540842	0.9567505121	-9.687641144
1	5	1700	0.7166618109	0.7129654884	-5.455255985
1	4	1700	0.6937904358	0.6962730289	-5.249244213
1	3	1700	0.7925998569	0.7540574074	-6.139259815
1	2	1700	0.6336572766	0.6383780241	-4.70760107
1	1	1700	1.127580404	0.9117973447	-9.156560898
1	5	1600	0.6768186688	0.6870008707	-5.096372604
1	4	1600	0.7472695112	0.7912863493	-5.730950832
1	3	1600	0.7506099343	0.702604413	-5.761039734
1	2	1600	0.8048680425	0.7164966464	-6.249763966
1	1	1600	1.105330706	0.9214162827	-8.956150055
1	5	1500	0.7974420786	0.7533239126	-6.182875633
1	4	1500	0.8587694168	0.7801576853	-6.735274792
1	3	1500	0.8403714895	0.779299438	-6.569557667
1	2	1500	0.6333913803	0.6496180296	-4.705205917
1	1	1500	1.333083391	1.024585605	-11.00760746
1	5	1400	0.7253412008	0.7465719581	-5.533434391
1	4	1400	0.9989373088	0.8041178584	-7.997822762

1	3	1400	0.9075397253	0.7768939137	-7.174568176
1	2	1400	0.7133669257	0.7010281682	-5.425577164
1	1	1400	1.334081411	1.033024669	-11.01659775
1	5	1300	0.9852073789	0.834335804	-7.87415123
1	4	1300	0.8380178213	0.7067549825	-6.54835701
1	3	1300	0.6807283163	0.68534863	-5.131588459
1	2	1300	0.7378931046	0.7267097235	-5.646494389
1	1	1300	1.66043222	1.152582884	-13.95616817
1	5	1200	0.434984684	0.54843539	-2.918078661
1	4	1200	0.9293435812	0.7770869136	-7.37096405
1	3	1200	0.7875216007	0.7213905454	-6.09351778
1	2	1200	0.8373254538	0.7696192861	-6.542120934
1	1	1200	1.576843143	1.125044584	-13.20324802
1	5	1100	0.7461415529	0.7005993128	-5.720790863
1	4	1100	0.8724048734	0.7319310308	-6.858094692
1	3	1100	0.9681669474	0.850628078	-7.720661163
1	2	1100	0.5854467154	0.6258322597	-4.273349285
1	1	1100	1.76733017	1.215090513	-14.91903973
1	5	1000	0.7976706028	0.7374491096	-6.184933662
1	4	1000	0.6795555353	0.6825877428	-5.121024609
1	3	1000	0.7480747104	0.6716580987	-5.738204002
1	2	1000	0.7673137188	0.7467979193	-5.911497116
1	1	1000	1.753231764	1.177200913	-14.79205036
1	5	900	0.7156662345	0.7283498049	-5.446288109
1	4	900	0.7397791147	0.7010387182	-5.663482666
1	3	900	0.7980782986	0.7509505749	-6.188605785
1	2	900	0.6365656853	0.6881777048	-4.733798027
1	1	900	1.850157976	1.214247942	-15.66510201
1	5	800	0.7938241363	0.7348892093	-6.150287151
1	4	800	0.8406608701	0.7558576465	-6.572164059
1	3	800	0.8927315474	0.7776848078	-7.041185379
1	2	800	0.7223138213	0.7244547009	-5.506165504
1	1	800	2.787332058	1.529105425	-24.1065979
1	5	700	0.8044689298	0.7262617946	-6.246168613
1	4	700	0.7339082956	0.6754136682	-5.610601425

1	3	700	0.8382486105	0.7667943835	-6.55043602
1	2	700	0.8208398819	0.773278296	-6.393628597
1	1	700	2.545150757	1.483948112	-21.92517662
1	5	600	0.5751644373	0.6480192542	-4.180733204
1	4	600	0.6193489432	0.6423226595	-4.578720093
1	3	600	0.9744960666	0.7950732112	-7.77767086
1	2	600	0.7271279097	0.7360081077	-5.549528122
1	1	600	3.095847607	1.626501799	-26.88551903
1	5	500	0.6581066251	0.6392448545	-4.927825928
1	4	500	0.6779827476	0.6460546255	-5.106858253
1	3	500	0.7642322779	0.7220022678	-5.883741379
1	2	500	0.8063343763	0.7550505996	-6.262971878
1	1	500	3.474116087	1.759567857	-30.29273415
1	5	400	0.7745116949	0.7082787752	-5.976332664
1	4	400	0.6276021004	0.6623459458	-4.653059483
1	3	400	0.6283239126	0.6456508636	-4.659561157
1	2	400	1.088171244	0.9083018303	-8.801587105
1	1	400	4.588820934	1.935151696	-40.33332062
1	5	300	0.4373353124	0.566727519	-2.939251423
1	4	300	0.7817600369	0.758041203	-6.041620731
1	3	300	0.9343057871	0.7882011533	-7.415660858
1	2	300	0.864390254	0.8339188695	-6.785903931
1	1	300	5.451209068	2.119271278	-48.10119247
1	5	200	0.6161950231	0.6581181288	-4.550311565
1	4	200	0.3992568851	0.5067126155	-2.596264362
1	3	200	0.7555000186	0.7509515882	-5.805086613
1	2	200	1.199038029	0.909693718	-9.800208092
1	1	200	6.232726574	2.222792864	-55.14062881
1	5	120	0.7233833075	0.7722937465	-5.515799046
1	4	120	1.171999931	0.9408705831	-9.556665421
1	3	120	1.042012572	0.8835274577	-8.385818481
1	2	120	1.118036151	0.8931998014	-9.07059288
1	1	120	7.277992249	2.431043863	-64.55574799
1	5	60	1.015077233	0.8463400006	-8.143200874
1	4	60	0.5357901454	0.5867851973	-3.82607317

1	3	60	1.025495529	0.8226495981	-8.237042427
1	2	60	3.548249722	1.763624907	-30.96048546
1	1	60	7.163125038	2.428289413	-63.52108765
1	5	30	2.473006487	1.469680071	-21.27534485
1	4	30	3.425296068	1.693806291	-29.85299301
1	3	30	4.258665562	1.935350776	-37.359478
1	2	30	6.256563663	2.256596088	-55.35533524
1	1	30	8.936886787	2.839442968	-79.49806213
2	25	2500	0.11102359	0.2918872237	-3.33E-05
2	24	2500	0.08453030139	0.2402699888	0.2386022806
2	25	2400	0.1110726744	0.2918871343	-0.0004754066467
2	24	2400	0.1020644531	0.2787695527	0.08066523075
2	25	2300	0.1110439077	0.2918871045	-0.0002162456512
2	24	2300	0.1108986884	0.2888867259	0.001091778278
2	25	2200	0.1111745015	0.2918760777	-0.001392602921
2	24	2200	0.1110217199	0.2918872237	-1.65E-05
2	25	2100	0.07053096592	0.2121945918	0.3646997213
2	24	2100	0.1123735756	0.2874467373	-0.01219308376
2	25	2000	0.1110314354	0.2918872833	-0.0001039505005
2	24	2000	0.08165191114	0.2254633009	0.2645289898
2	25	1900	0.0712769255	0.2314617187	0.3579806089
2	24	1900	0.05357626826	0.1774529964	0.5174174309
2	25	1800	0.1111067906	0.2918871939	-0.0007826089859
2	24	1800	0.0772170797	0.2139208764	0.3044753075
2	25	1700	0.1120852083	0.2918872237	-0.009595751762
2	24	1700	0.1110270023	0.2918870449	-6.40E-05
2	25	1600	0.1110651344	0.2918873429	-0.0004074573517
2	24	1600	0.1110303998	0.2918872833	-9.47E-05

2	25	1500	0.1117095947	0.2918872237	-0.006212353706
2	24	1500	0.1113958135	0.2918872237	-0.00338602066
2	25	1400	0.1110384241	0.2918865681	-0.0001668930054
2	24	1400	0.1111430377	0.2918810248	-0.001109242439
2	25	1300	0.0982228294	0.2266095579	0.1152682304
2	24	1300	0.1110568643	0.2918871939	-0.0003329515457
2	25	1200	0.1114087701	0.2918872237	-0.003502726555
2	24	1200	0.06966027617	0.24218373	0.3725424409
2	25	1100	0.1042425856	0.2748215795	0.06104588509
2	24	1100	0.05893976614	0.2144807279	0.469106257
2	25	1000	0.1122244745	0.2918871045	-0.01085007191
2	24	1000	0.06173516437	0.2142172754	0.4439270496
2	25	900	0.08061269671	0.2608982027	0.2738896608
2	24	900	0.1116757393	0.2918854356	-0.005907416344
2	25	800	0.1126597375	0.2916257381	-0.01477074623
2	24	800	0.04043182731	0.1806778908	0.6358145475
2	25	700	0.1231393069	0.2918854654	-0.1091643572
2	24	700	0.05301455781	0.1880814135	0.5224769711
2	25	600	0.03540484235	0.1509039402	0.6810946465
2	24	600	0.03958470374	0.1674249619	0.6434449553
2	25	500	0.0855435729	0.2548030019	0.2294753194
2	24	500	0.1016205922	0.2775413096	0.0846632719
2	25	400	0.04979567602	0.1764312685	0.5514707565
2	24	400	0.04995971173	0.1915597916	0.5499931574
2	25	300	0.04028797895	0.1765558273	0.6371102929
2	24	300	0.04025772586	0.168943733	0.6373828053
2	25	200	0.03512858972	0.1406663358	0.683582902
2	24	200	0.04375602677	0.1686533391	0.6058722138

2	25	120	0.03968094289	0.1438892782	0.642578125
2	24	120	0.05715398118	0.2201615274	0.485191524
2	25	60	0.06165554374	0.1850316972	0.4446442127
2	24	60	0.06718476117	0.2211570293	0.3948403597
2	25	30	0.1459441036	0.3440655172	-0.314576149
2	24	30	0.05837485194	0.1939277649	0.4741947055
2	23	2500	0.07961131632	0.2256927043	0.2829095125
2	22	2500	0.111647442	0.2918872237	-0.005652546883
2	21	2500	0.05058019236	0.1864852458	0.5444042683
2	23	2400	0.1049189791	0.2625047266	0.05495333672
2	22	2400	0.100570336	0.2606256902	0.09412336349
2	21	2400	0.04708014801	0.1795348674	0.5759305358
2	23	2300	10.55329132	2.941648245	-94.05765533
2	22	2300	0.1122540608	0.2917147577	-0.01111662388
2	21	2300	0.08903099597	0.2548669875	0.1980627179
2	23	2200	0.1119948998	0.2918872833	-0.00878226757
2	22	2200	0.09882338345	0.2695391178	0.1098588109
2	21	2200	0.06459774822	0.2417954952	0.4181426167
2	23	2100	0.07542616129	0.221216917	0.3206068277
2	22	2100	0.111082375	0.2918866277	-0.000562787056
2	21	2100	0.07926757634	0.2469751388	0.2860056758
2	23	2000	0.1110296026	0.2918871045	-8.74E-05
2	22	2000	0.1110843867	0.2918871343	-0.000580906868
2	21	2000	0.1404342949	0.3153015971	-0.2649470568
2	23	1900	0.1110846847	0.2918866277	-0.0005836486816
2	22	1900	0.06821481884	0.2163311988	0.3855622411
2	21	1900	0.05681322888	0.2010332644	0.4882608652
2	23	1800	0.07133551687	0.2264853418	0.3574528694
2	22	1800	0.1109866947	0.2918305993	0.0002990365028
2	21	1800	0.05959958956	0.2119046152	0.4631629586

2	23	1700	0.1001451761	0.2797114849	0.09795290232
2	22	1700	0.09886537492	0.2650032938	0.1094805598
2	21	1700	0.05954272673	0.192720741	0.4636752009
2	23	1600	0.06632809341	0.2408305109	0.4025566578
2	22	1600	0.05445238948	0.1527802497	0.5095258951
2	21	1600	0.07444553822	0.2193486691	0.32943964
2	23	1500	0.1086435467	0.2843267322	0.02140474319
2	22	1500	0.06758859754	0.2210955173	0.391202867
2	21	1500	0.06663624942	0.2193936408	0.3997810483
2	23	1400	0.1114773527	0.2918838561	-0.0041204690<br>93
2	22	1400	0.09825949371	0.2735112309	0.1149380207
2	21	1400	0.1004475802	0.268102169	0.09522902966
2	23	1300	0.08026736975	0.239776656	0.2770001292
2	22	1300	0.06835380942	0.1984539926	0.3843103051
2	21	1300	0.06463736296	0.1908357143	0.4177857637
2	23	1200	0.1110348925	0.2918860018	-0.0001350641<br>251
2	22	1200	0.06336318702	0.2271221131	0.4292627573
2	21	1200	0.04160648957	0.1722784936	0.6252339482
2	23	1100	0.111021325	0.2918864787	-1.29E-05
2	22	1100	0.1138749346	0.2916209102	-0.0257164239<br>9
2	21	1100	0.0722765401	0.2138531655	0.3489767313
2	23	1000	0.111009635	0.2918654978	9.24E-05
2	22	1000	0.0314854607	0.1243404374	0.7163980603
2	21	1000	0.03558364511	0.1704567969	0.6794840693
2	23	900	0.0534222424	0.2069860399	0.5188047886
2	22	900	0.05455420539	0.2060670853	0.5086087584
2	21	900	0.08100169152	0.2477394044	0.2703858018
2	23	800	0.05855015665	0.1904699355	0.4726156592
2	22	800	0.03863002732	0.1663582325	0.6520441175
2	21	800	0.0392623283	0.1570770741	0.6463487148
2	23	700	0.07463203371	0.2112498283	0.3277598619
2	22	700	0.04518833384	0.1713190973	0.5929708481

2	21	700	0.04616373777	0.1856014282	0.5841850042
2	23	600	0.04838200659	0.1891766042	0.564204216
2	22	600	0.05549592897	0.206776768	0.5001262426
2	21	600	0.03350637853	0.1646216363	0.6981948614
2	23	500	0.02527599037	0.1256371439	0.7723292112
2	22	500	0.0429113023	0.1767111272	0.6134809852
2	21	500	0.04276215658	0.1688075066	0.6148244143
2	23	400	0.04484312981	0.1792368442	0.5960802436
2	22	400	0.02772494592	0.1319625825	0.7502704859
2	21	400	0.03434555978	0.1407256573	0.6906359792
2	23	300	0.03531011194	0.1494383812	0.6819479465
2	22	300	0.07365301996	0.2289125919	0.3365781903
2	21	300	0.04603425041	0.1767164767	0.5853513479
2	23	200	0.03696117923	0.1484313458	0.6670761108
2	22	200	0.03438600153	0.1385287344	0.6902717352
2	21	200	0.118613936	0.2999518812	-0.0684025287<br>6
2	23	120	0.08642062545	0.2567695677	0.2215753198
2	22	120	0.07259577513	0.237131685	0.3461012244
2	21	120	0.09434645623	0.2751716077	0.1501842737
2	23	60	0.2943175137	0.4954039454	-1.651033878
2	22	60	0.04119313881	0.1621417999	0.6289571524
2	21	60	0.04161295295	0.1719954461	0.6251757145
2	23	30	0.0677850768	0.1890277416	0.3894330859
2	22	30	0.1754640043	0.2917804122	-0.5804735422
2	21	30	0.2965423465	0.5010792017	-1.671073675
2	20	2500	0.05416681245	0.187056452	0.5120981932
2	19	2500	0.04361083359	0.1767998636	0.6071799994
2	18	2500	0.1270172894	0.295753181	-0.1440947056
2	17	2500	0.05416256189	0.1776367128	0.5121364594
2	16	2500	0.03956963122	0.1492806971	0.6435807347
2	20	2400	0.04970294237	0.1910910159	0.5523059964
2	19	2400	0.1138344854	0.2809764445	-0.0253521204
2	18	2400	6.406734467	2.463784695	-56.70798492
2	17	2400	0.06188004464	0.2188130319	0.4426220059

2	16	2400	0.07574383169	0.2185670882	0.3177454472
2	20	2300	0.06277159601	0.2274601012	0.4345914721
2	19	2300	0.08081127703	0.2332613021	0.2721009254
2	18	2300	0.09438201785	0.2265377939	0.1498639584
2	17	2300	0.04932084307	0.1946916133	0.5557477474
2	16	2300	0.0622857213	0.1959570348	0.4389679432
2	20	2200	0.05944986269	0.2038346231	0.4645116329
2	19	2200	0.06852693111	0.2156405002	0.3827509284
2	18	2200	0.05501261353	0.1942253113	0.5044797063
2	17	2200	0.06284756958	0.2204588354	0.4339071512
2	16	2200	0.05757143348	0.1989659369	0.481431365
2	20	2100	0.05950100347	0.2170713842	0.4640510082
2	19	2100	0.0721739307	0.2109415978	0.3499009609
2	18	2100	0.1035603285	0.2794352472	0.06719130278
2	17	2100	0.1108347028	0.2911817431	0.001668095589
2	16	2100	0.0495423004	0.182649374	0.5537529588
2	20	2000	0.07769858092	0.2436291724	0.3001382351
2	19	2000	0.8875427246	0.678989768	-6.994447231
2	18	2000	0.05320192128	0.1975528747	0.5207893252
2	17	2000	0.07446177304	0.2166712284	0.3292934895
2	16	2000	0.03376091272	0.1395533979	0.6959021091
2	20	1900	0.06053169444	0.1904242039	0.4547671676
2	19	1900	0.1193857342	0.2720153928	-0.07535433769
2	18	1900	0.04492805153	0.1819983423	0.5953153372
2	17	1900	0.04745656252	0.189639613	0.5725400448
2	16	1900	0.04145053774	0.1792864352	0.6266386509
2	20	1800	0.04324465618	0.1871760786	0.610478282
2	19	1800	0.08065777272	0.2624304891	0.273483634
2	18	1800	0.04094038904	0.1717338115	0.6312338114
2	17	1800	0.04544733837	0.1766034663	0.5906379223
2	16	1800	0.4592944086	0.6004685163	-3.13704586
2	20	1700	0.1110825166	0.291867882	-0.0005640983582

2	19	1700	0.05538481474	0.1834268123	0.5011271238
2	18	1700	0.05926845223	0.2023018897	0.4661456943
2	17	1700	0.04874209687	0.1926629096	0.56096071
2	16	1700	0.04103900865	0.1786295921	0.6303454638
2	20	1600	0.05882974342	0.2028499097	0.4700973034
2	19	1600	0.1103581041	0.283999145	0.005961000919
2	18	1600	0.05018835142	0.2040072381	0.5479336977
2	17	1600	0.04658756778	0.1794287711	0.5803673863
2	16	1600	0.03936039284	0.1632910669	0.6454654336
2	20	1500	0.06773547083	0.2309166938	0.3898799419
2	19	1500	0.05139933899	0.1993526518	0.5370259285
2	18	1500	0.05279467255	0.1806010306	0.5244575739
2	17	1500	0.06410668045	0.1923047006	0.4225658774
2	16	1500	0.04294674844	0.1825690269	0.6131616831
2	20	1400	0.06190207601	0.2026888877	0.4424235821
2	19	1400	0.05450846627	0.206425339	0.5090207458
2	18	1400	0.04928313941	0.1787543297	0.5560873747
2	17	1400	0.05487359315	0.2021072358	0.5057319403
2	16	1400	0.04059332609	0.1734000146	0.6343598962
2	20	1300	0.0604765527	0.220566839	0.4552638531
2	19	1300	0.09186373651	0.2680617273	0.1725470424
2	18	1300	0.0576111488	0.2133761346	0.4810736775
2	17	1300	0.05706639215	0.1999777257	0.4859805107
2	16	1300	0.1972730756	0.3942916989	-0.7769163847
2	20	1200	0.05952066183	0.2125842124	0.4638739228
2	19	1200	0.05583697557	0.210495472	0.4970543385
2	18	1200	0.04530893639	0.178194806	0.5918845534
2	17	1200	0.05723003298	0.1948471963	0.4845065475
2	16	1200	0.04682607204	0.192185685	0.5782191157
2	20	1100	0.04819555208	0.178378731	0.5658836365
2	19	1100	0.05044318363	0.1885220557	0.5456383228
2	18	1100	0.03868380934	0.1658185422	0.6515597105
2	17	1100	0.0508374162	0.2002233565	0.5420873165
2	16	1100	0.03546551988	0.1474471092	0.6805480719

2	20	1000	0.03370775655	0.1493814886	0.6963809729
2	19	1000	0.040934477	0.1789233685	0.6312870383
2	18	1000	0.06734871119	0.2134047449	0.393363595
2	17	1000	0.044519227	0.1645219326	0.5989977717
2	16	1000	0.05747173354	0.1808805913	0.4823294282
2	20	900	0.05239901692	0.1968211681	0.5280213952
2	19	900	0.07104212791	0.219635874	0.3600955606
2	18	900	0.03953151777	0.1645557433	0.6439239979
2	17	900	0.03173902258	0.1347498	0.7141140699
2	16	900	0.02777639963	0.1302261353	0.7498070002
2	20	800	0.1063947305	0.2699847817	0.04166066647
2	19	800	0.03745809942	0.1764430106	0.6626001596
2	18	800	0.04675997049	0.1854673922	0.5788145065
2	17	800	0.04134168848	0.1730780602	0.6276190877
2	16	800	0.03012989834	0.1413370073	0.7286081314
2	20	700	0.08127366006	0.2587530017	0.2679360509
2	19	700	0.05108414218	0.1887844056	0.5398650169
2	18	700	0.04483537376	0.1933914721	0.5961501002
2	17	700	0.03431254625	0.1515709907	0.6909333467
2	16	700	0.039801687	0.1685015708	0.641490519
2	20	600	0.04377291352	0.1611394435	0.6057201624
2	19	600	0.04961838573	0.1767401695	0.5530676246
2	18	600	0.05199377611	0.199155286	0.531671524
2	17	600	0.04866686463	0.1692821532	0.5616383553
2	16	600	0.3479382396	0.5356529355	-2.134016991
2	20	500	0.03859484941	0.142512843	0.6523609161
2	19	500	0.03672255203	0.1416606009	0.6692254543
2	18	500	0.1284860373	0.3169694841	-0.1573244333
2	17	500	0.04300120845	0.17589131	0.6126711369
2	16	500	0.1983746588	0.3915922046	-0.7868387699
2	20	400	0.03699592501	0.1646465808	0.6667631269
2	19	400	0.03499467298	0.1648623049	0.6847891808
2	18	400	0.03399527445	0.1492331028	0.693791151
2	17	400	0.02216133289	0.109333083	0.8003841639
2	16	400	0.04312477633	0.1637424529	0.6115581393

2	20	300	0.04148013517	0.1390915364	0.6263720989
2	19	300	0.05469670147	0.2012488842	0.507325232
2	18	300	0.1936267167	0.3973881602	-0.7440721989
2	17	300	0.0603877902	0.1721586287	0.4560633302
2	16	300	0.1592536122	0.353759855	-0.4344600439
2	20	200	0.05670139939	0.2065027654	0.4892681241
2	19	200	0.04458851367	0.1821608543	0.5983736515
2	18	200	0.1149700135	0.3045117855	-0.0355802774<br>4
2	17	200	0.1263547242	0.2942652106	-0.1381267309
2	16	200	0.1934299916	0.3926934302	-0.742300272
2	20	120	0.1166427284	0.287738502	-0.0506470203<br>4
2	19	120	0.1072242409	0.2801514566	0.03418898582
2	18	120	0.09543581307	0.2738854885	0.1403719783
2	17	120	0.2587443292	0.4627905488	-1.330612183
2	16	120	0.1638078392	0.3501979709	-0.4754818678
2	20	60	0.06662885845	0.1814536154	0.3998476267
2	19	60	0.1996169984	0.3997415602	-0.7980290651
2	18	60	0.1391629875	0.3074189723	-0.2534959316
2	17	60	0.1679209173	0.3274611831	-0.5125299692
2	16	60	0.2186493427	0.3591472208	-0.969460845
2	20	30	0.235598892	0.3743869662	-1.122132301
2	19	30	0.2398757935	0.3837510049	-1.160655975
2	18	30	0.7949341536	0.7927731872	-6.160285473
2	17	30	0.1153356805	0.2691049874	-0.0388739109
2	16	30	0.04237275943	0.1478286684	0.6183318496
2	15	2500	0.0499551408	0.1926860809	0.5500343442
2	14	2500	0.06360828131	0.2135932893	0.4270551205
2	13	2500	0.03132914007	0.1426668614	0.7178061008
2	12	2500	0.03348856419	0.1372294426	0.6983553171
2	11	2500	0.0319035463	0.1477847993	0.7126321793
2	15	2400	0.06090840697	0.2015992105	0.4513739347
2	14	2400	0.03511964157	0.1496489942	0.683663547
2	13	2400	0.05366751552	0.2019988	0.5165954828

2	12	2400	0.04612593725	0.168964535	0.584525466
2	11	2400	0.07280345261	0.2549176812	0.3442305923
2	15	2300	0.04935730249	0.1906712949	0.5554193258
2	14	2300	0.04338699579	0.1653045714	0.6091961861
2	13	2300	0.05925731733	0.2040863037	0.4662460089
2	12	2300	0.02963116206	0.1324497759	0.7331004143
2	11	2300	0.04656948149	0.1727414131	0.5805302858
2	15	2200	0.3861600757	0.4539269805	-2.47829628
2	14	2200	0.03083523549	0.1500855982	0.7222548723
2	13	2200	0.04409977049	0.1759531051	0.602775991
2	12	2200	0.03857877851	0.1614434272	0.6525057554
2	11	2200	0.1880844384	0.4061702788	-0.6941506863
2	15	2100	0.05444784835	0.2003457099	0.5095667839
2	14	2100	0.04691373184	0.1778244525	0.577429533
2	13	2100	0.03884058073	0.1585389078	0.6501475573
2	12	2100	0.05861722305	0.2047677487	0.4720115662
2	11	2100	0.03582013771	0.1546028554	0.6773538589
2	15	2000	0.04603912681	0.1854796857	0.5853074789
2	14	2000	0.04323453829	0.1812837124	0.6105694771
2	13	2000	0.04083815962	0.1834270954	0.6321545839
2	12	2000	0.0385110341	0.1640175283	0.6531159282
2	11	2000	0.07033272833	0.2548757195	0.3664853573
2	15	1900	0.05975977331	0.2216149867	0.4617201686
2	14	1900	0.03582245857	0.1381216496	0.6773329973
2	13	1900	0.04426533729	0.1633132398	0.6012846231
2	12	1900	0.05650258064	0.2132148743	0.4910589457
2	11	1900	0.02723639831	0.126379922	0.7546709776
2	15	1800	0.03894562274	0.1385148466	0.6492013931
2	14	1800	0.06122180074	0.2146880627	0.4485510588
2	13	1800	0.04660676792	0.1865848601	0.5801944733
2	12	1800	0.290395081	0.4916450381	-1.615702868
2	11	1800	0.04366539419	0.1670705825	0.6066886187
2	15	1700	0.03204255551	0.1469232589	0.7113800645
2	14	1700	0.2050716877	0.4079046845	-0.8471615314
2	13	1700	0.09147782624	0.2768092752	0.1760231256

2	12	1700	0.1125703081	0.3143622875	-0.01396512985
2	11	1700	0.770442009	0.737390399	-5.939674854
2	15	1600	0.04012076557	0.1754746437	0.6386164427
2	14	1600	0.04893568903	0.1796589345	0.5592169762
2	13	1600	0.04351632297	0.1588606387	0.6080312729
2	12	1600	0.03853750974	0.1511539966	0.65287745
2	11	1600	0.1783631742	0.3754166365	-0.60658741
2	15	1500	0.1065590978	0.2905724645	0.0401802063
2	14	1500	0.0399640426	0.1577771157	0.6400281191
2	13	1500	0.04919041321	0.209278062	0.556922555
2	12	1500	0.03840004653	0.1652960777	0.6541156173
2	11	1500	0.2351022214	0.4566346109	-1.117658377
2	15	1400	0.03501341492	0.1603643447	0.6846203804
2	14	1400	0.04392518848	0.1836183071	0.6043484807
2	13	1400	0.09964932501	0.2723939419	0.1024191976
2	12	1400	0.1544343531	0.3622862399	-0.391051054
2	11	1400	0.1812832355	0.3801773489	-0.6328896284
2	15	1300	0.03340316564	0.1541949809	0.6991245151
2	14	1300	0.03754260018	0.1511853188	0.6618390083
2	13	1300	0.0446880199	0.1779462844	0.5974773765
2	12	1300	0.1265275776	0.3256413341	-0.1396837234
2	11	1300	0.05676745623	0.2136688679	0.4886730909
2	15	1200	0.0333910659	0.1488312185	0.699233532
2	14	1200	0.03667423129	0.1714820862	0.6696607471
2	13	1200	0.343901813	0.4836068153	-2.097659111
2	12	1200	0.05394370481	0.1800851375	0.5141077638
2	11	1200	0.454274714	0.5794168711	-3.091831207
2	15	1100	0.3098524511	0.5092867613	-1.790963173
2	14	1100	0.03625737876	0.1464754641	0.673415482
2	13	1100	0.05199009925	0.1520446837	0.5317046642
2	12	1100	0.6997847557	0.6904029846	-5.303237438
2	11	1100	0.502558589	0.6247750521	-3.526743412
2	15	1000	0.04949049279	0.1821120232	0.5542196035
2	14	1000	0.1242798343	0.3275713921	-0.1194374561

2	13	1000	0.116349414	0.3254154325	-0.04800498486
2	12	1000	0.5240887403	0.6251446605	-3.720674038
2	11	1000	0.3535951972	0.5322757959	-2.184971333
2	15	900	0.1496211439	0.3010292947	-0.3476966619
2	14	900	0.03035576083	0.151208207	0.7265737057
2	13	900	0.1728313863	0.3859142661	-0.5567605495
2	12	900	0.1235482693	0.3277406096	-0.1128480434
2	11	900	0.04403717816	0.1871265471	0.6033397913
2	15	800	0.03404927999	0.1556292474	0.6933047175
2	14	800	0.04068401456	0.1712121964	0.6335430145
2	13	800	0.1179836243	0.315564394	-0.06272506714
2	12	800	0.08991090953	0.2852879167	0.1901370287
2	11	800	0.3406592011	0.4374467731	-2.068451643
2	15	700	0.0351155065	0.1673210561	0.6837007999
2	14	700	0.128380686	0.3309455514	-0.1563754082
2	13	700	0.4101317823	0.5795475245	-2.694218874
2	12	700	0.1697273552	0.391748786	-0.5288012028
2	11	700	0.2300497591	0.4410893917	-1.0721488
2	15	600	0.2141036093	0.3902445138	-0.9285155535
2	14	600	0.2061724961	0.3892016411	-0.8570768833
2	13	600	0.297662884	0.4497274458	-1.681166887
2	12	600	0.2874418199	0.4580880105	-1.589101791
2	11	600	0.5382525921	0.6145508289	-3.84825325
2	15	500	0.04028048366	0.156891197	0.637177825
2	14	500	0.2289605141	0.4459160268	-1.062337637
2	13	500	0.2314742506	0.4337502122	-1.084980011
2	12	500	0.1147477403	0.2980944216	-0.03357815742
2	11	500	0.5758634806	0.6241117716	-4.187029839
2	15	400	0.1747532189	0.3845673203	-0.5740710497
2	14	400	0.08300554752	0.2716667652	0.2523363233
2	13	400	0.2862746119	0.4425701499	-1.578588486
2	12	400	0.2825356424	0.4518126845	-1.544909954

2	11	400	0.195932433	0.3968693316	-0.7648407221
2	15	300	0.1130602583	0.3111051917	-0.01837837696
2	14	300	0.3079714775	0.4761431813	-1.774020672
2	13	300	0.2612705827	0.4464048445	-1.35336709
2	12	300	0.2417147905	0.4136398435	-1.177220345
2	11	300	0.3088217676	0.4588752687	-1.781679392
2	15	200	0.1991434991	0.3785487115	-0.7937641144
2	14	200	0.3160905242	0.5192252398	-1.847152233
2	13	200	0.2803148627	0.4783340394	-1.524906635
2	12	200	0.3169099987	0.4377842844	-1.854533434
2	11	200	0.3944176137	0.5173377991	-2.552675009
2	15	120	0.1595124304	0.3465217054	-0.43679142
2	14	120	0.2301793396	0.3968507648	-1.073316097
2	13	120	0.09957682341	0.3006106913	0.1030722857
2	12	120	0.2659114897	0.4751781523	-1.395169497
2	11	120	0.3045101166	0.4483820796	-1.742842674
2	15	60	0.2738009691	0.4667021334	-1.466233253
2	14	60	0.1232729182	0.2849588394	-0.110367775
2	13	60	0.3558727801	0.5752708316	-2.205486536
2	12	60	0.3192355037	0.5005482435	-1.875479937
2	11	60	0.3093481958	0.4462311864	-1.786421299
2	15	30	0.6136286259	0.7412122488	-4.527194977
2	14	30	0.2185053527	0.4054307044	-0.9681639671
2	13	30	0.1880225241	0.3745232522	-0.6935930252
2	12	30	0.9773514867	0.9368928671	-7.803390503
2	11	30	0.3475679755	0.5587615371	-2.130681992
2	10	2500	0.0348681882	0.1288189888	0.6859284639
2	9	2500	0.1167355031	0.3087793291	-0.05148279667
2	8	2500	0.379203856	0.543664813	-2.415638685
2	7	2500	0.5988443494	0.6566621661	-4.394027233
2	6	2500	0.6705670357	0.6738069057	-5.040061951
2	10	2400	0.02886629105	0.1533935517	0.7399899364
2	9	2400	0.0431484282	0.1786716878	0.6113450527

2	8	2400	0.4284875989	0.556270957	-2.859556675
2	7	2400	0.5892444849	0.5810490251	-4.307557106
2	6	2400	1.093076348	0.8921922445	-8.845769882
2	10	2300	0.1624182314	0.3641201854	-0.4629651308
2	9	2300	0.1462149173	0.3328230977	-0.3170154095
2	8	2300	0.7698369026	0.7409884334	-5.934224606
2	7	2300	0.5673978925	0.6661216021	-4.110776424
2	6	2300	0.5553429723	0.6303479075	-4.002192974
2	10	2200	0.07901452482	0.2561094165	0.2882849574
2	9	2200	0.06962607801	0.2250908911	0.3728504181
2	8	2200	0.367864877	0.515663445	-2.313503981
2	7	2200	0.3796721101	0.5411081314	-2.419856548
2	6	2200	0.4559336603	0.6232998967	-3.10677433
2	10	2100	0.4660433233	0.6124299765	-3.197835922
2	9	2100	0.5142668486	0.6375902891	-3.632204056
2	8	2100	0.4111860693	0.5418683887	-2.703715086
2	7	2100	0.5488653779	0.6312901378	-3.943846703
2	6	2100	0.7476508617	0.7254391909	-5.734385967
2	10	2000	0.08349700272	0.2467140257	0.2479095459
2	9	2000	0.5403038263	0.6090518832	-3.866729259
2	8	2000	0.7793289423	0.7203575969	-6.019723415
2	7	2000	0.5318146944	0.6302958727	-3.790265083
2	6	2000	0.5478682518	0.615280807	-3.934865475
2	10	1900	0.08613671362	0.2695749402	0.2241326571
2	9	1900	0.2797321379	0.442587465	-1.519657612
2	8	1900	0.5209777951	0.594353199	-3.692652225
2	7	1900	0.4453573823	0.5378248096	-3.011509418
2	6	1900	0.4950809479	0.5790215731	-3.45938921
2	10	1800	0.03914508224	0.1730425805	0.6474047899
2	9	1800	0.3918581307	0.568114996	-2.529620647
2	8	1800	0.7029387355	0.7085937262	-5.331646442
2	7	1800	0.4873049259	0.5908055305	-3.389347553
2	6	1800	0.4229406416	0.5610786676	-2.809593201
2	10	1700	0.1260754168	0.3049381971	-0.1356109381
2	9	1700	0.4775952399	0.5826799273	-3.301888466

2	8	1700	0.5849224329	0.6339620948	-4.268627167
2	7	1700	0.497374624	0.5995779037	-3.48004961
2	6	1700	0.5904974937	0.6563145518	-4.318843842
2	10	1600	0.4135534763	0.5460250974	-2.725039244
2	9	1600	0.4782591462	0.6054133177	-3.307868481
2	8	1600	0.6170214415	0.6495357752	-4.55775547
2	7	1600	0.3906987906	0.5310609341	-2.519178152
2	6	1600	0.5303448439	0.6282709241	-3.777024746
2	10	1500	0.3047670126	0.471875906	-1.745156765
2	9	1500	0.0805940479	0.2374288142	0.2740576267
2	8	1500	0.2862833142	0.4470860958	-1.578666925
2	7	1500	0.3755460382	0.5381657481	-2.382691145
2	6	1500	0.6519098282	0.7295438051	-4.872008801
2	10	1400	0.1104392782	0.279476881	0.005229890347
2	9	1400	0.54298985	0.6550875306	-3.890923977
2	8	1400	0.6612375379	0.6688818932	-4.956027031
2	7	1400	0.5208691955	0.6018480062	-3.691674232
2	6	1400	0.5533608794	0.6372511983	-3.984339714
2	10	1300	0.4710362852	0.5959793329	-3.242809772
2	9	1300	0.6900353432	0.7108133435	-5.215420246
2	8	1300	1.056284547	0.8864756823	-8.514370918
2	7	1300	0.261788249	0.4407885671	-1.358030081
2	6	1300	0.5582699776	0.6321467161	-4.028557777
2	10	1200	0.03949885815	0.1536014974	0.6442182064
2	9	1200	0.2382025272	0.4276928306	-1.145584106
2	8	1200	0.1225716844	0.2829240263	-0.1040514708
2	7	1200	0.5905860066	0.649882257	-4.319641113
2	6	1200	0.6633023024	0.6856295466	-4.974625587
2	10	1100	0.07059197128	0.2506462038	0.3641502857
2	9	1100	0.09685234725	0.2404953986	0.12761271
2	8	1100	0.5857359171	0.6590941548	-4.275954247
2	7	1100	0.4136330485	0.5444883108	-2.725756168
2	6	1100	0.5041443706	0.6258875728	-3.541027069
2	10	1000	0.1333260834	0.2935696542	-0.200920701

2	9	1000	0.1889753342	0.3756939769	-0.7021753788
2	8	1000	0.4173358381	0.5497674942	-2.759108543
2	7	1000	0.6048644781	0.6923384666	-4.448252678
2	6	1000	0.706866622	0.6192041636	-5.367026329
2	10	900	0.2176035941	0.3907800317	-0.9600412846
2	9	900	0.344283402	0.5034751892	-2.101096153
2	8	900	0.4572263658	0.5592755079	-3.118418217
2	7	900	0.6127415895	0.6311744452	-4.519205093
2	6	900	0.687746644	0.6440864801	-5.194805622
2	10	800	0.2857903838	0.4891405702	-1.574226618
2	9	800	0.3159657419	0.4620448053	-1.84602809
2	8	800	0.4867425859	0.5497265458	-3.384282589
2	7	800	0.5922138095	0.6355595589	-4.334303379
2	6	800	0.6731203794	0.6760490537	-5.06306076
2	10	700	0.2201737463	0.4426116943	-0.9831917286
2	9	700	0.369620055	0.5096706152	-2.329313755
2	8	700	0.3914418519	0.4844834805	-2.525871038
2	7	700	0.434576422	0.578228116	-2.914401054
2	6	700	0.6190266609	0.6315800548	-4.575817108
2	10	600	0.4205727577	0.5215784907	-2.788264751
2	9	600	0.4643371105	0.5758139491	-3.182467461
2	8	600	0.4663788378	0.5977581143	-3.200858116
2	7	600	0.5187715292	0.6180340052	-3.672780037
2	6	600	0.6005710959	0.6541422009	-4.409580708
2	10	500	0.4378014207	0.5633142591	-2.943449974
2	9	500	0.4859792292	0.5810448527	-3.377406597
2	8	500	0.5444890261	0.597527802	-3.904427052
2	7	500	0.6935111284	0.6888141036	-5.246727943
2	6	500	0.5411533117	0.6394542456	-3.874381065
2	10	400	0.3193517923	0.4989210963	-1.876527548
2	9	400	0.4784080982	0.5905688405	-3.3092103
2	8	400	0.5535945296	0.5991333723	-3.986444473
2	7	400	0.4747334421	0.5556587577	-3.276111603
2	6	400	0.6529112458	0.6392278671	-4.881029129
2	10	300	0.3377524316	0.4754734039	-2.042269468

2	9	300	0.4476836622	0.5779431462	-3.032463551
2	8	300	0.4727074206	0.5706965327	-3.257862091
2	7	300	0.5603461266	0.6267212033	-4.047258377
2	6	300	0.5748025179	0.6061657667	-4.177472591
2	10	200	0.2677186728	0.4604381621	-1.411447763
2	9	200	0.4091822505	0.5417972803	-2.685666084
2	8	200	0.4677409232	0.5564634204	-3.213127136
2	7	200	0.5466893911	0.6037203074	-3.924247265
2	6	200	0.6742655039	0.6923391223	-5.073375225
2	10	120	0.3194517493	0.4686090052	-1.877428055
2	9	120	0.2246755064	0.3947227597	-1.023741007
2	8	120	0.3127877712	0.4385432303	-1.81740284
2	7	120	0.6357957125	0.659303844	-4.726862431
2	6	120	0.4358002543	0.5508495569	-2.925424814
2	10	60	0.4657816291	0.6193081737	-3.195478916
2	9	60	0.4125990868	0.5435558558	-2.716442585
2	8	60	0.5265948176	0.6407731771	-3.743247509
2	7	60	0.5147787929	0.6158310771	-3.636815548
2	6	60	0.3420907557	0.5165041685	-2.081346273
2	10	30	0.5440911055	0.6039292216	-3.900843143
2	9	30	2.07264924	1.322395563	-17.66916847
2	8	30	0.8031233549	0.6897998452	-6.234048843
2	7	30	0.8084148169	0.7965527773	-6.281711102
2	6	30	3.282051563	1.664249659	-28.5627327
2	5	2500	0.6173039675	0.670863986	-4.56030035
2	4	2500	0.8492034674	0.7826226354	-6.649110794
2	3	2500	0.5317389369	0.6397596598	-3.789582253
2	2	2500	0.8112341166	0.7507668734	-6.307105541
2	1	2500	0.8096210361	0.771117568	-6.292575836
2	5	2400	0.6086607575	0.6578410268	-4.482447147
2	4	2400	0.9970418811	0.8379094005	-7.98074913
2	3	2400	0.9037765265	0.7908416986	-7.14067173
2	2	2400	1.032225251	0.8773962855	-8.297659874
2	1	2400	0.8745290637	0.7782473564	-6.87722826
2	5	2300	0.8265789747	0.7402973175	-6.44532299

2	4	2300	0.840413928	0.7371726036	-6.56994009
2	3	2300	1.022930384	0.8595947027	-8.213936806
2	2	2300	0.7957369089	0.7222554088	-6.167515755
2	1	2300	0.839364171	0.7674234509	-6.560483932
2	5	2200	0.8912107348	0.7736905813	-7.027486801
2	4	2200	0.8768018484	0.7731766701	-6.89770031
2	3	2200	0.580889225	0.6461926103	-4.232298374
2	2	2200	0.7944415808	0.7561212182	-6.15584898
2	1	2200	0.9602950215	0.8526831865	-7.649755478
2	5	2100	0.6970031261	0.7078076601	-5.27818203
2	4	2100	0.7339799404	0.6744617224	-5.611246586
2	3	2100	0.912422955	0.7566848993	-7.218553543
2	2	2100	0.7593194842	0.7042124867	-5.839489937
2	1	2100	0.812294662	0.7741971016	-6.316658497
2	5	2000	0.4541319907	0.5241427422	-3.090545654
2	4	2000	0.6962608099	0.685233891	-5.271495342
2	3	2000	0.8507404327	0.7782007456	-6.662954807
2	2	2000	0.6282054186	0.6518067122	-4.658493519
2	1	2000	0.9548597336	0.8348089457	-7.600798607
2	5	1900	0.7580821514	0.7082357407	-5.828344822
2	4	1900	0.642750144	0.6544507146	-4.789504051
2	3	1900	0.7326819897	0.6904216409	-5.599555492
2	2	1900	0.8181056976	0.7334935069	-6.369000435
2	1	1900	1.028221846	0.8636945486	-8.261599541
2	5	1800	0.470970571	0.6009255648	-3.242218018
2	4	1800	1.02461946	0.9003303647	-8.229151726
2	3	1800	0.4661521018	0.6102856994	-3.198815823
2	2	1800	0.7989581227	0.7413800359	-6.196530819
2	1	1800	1.212404013	0.9978988767	-9.920601845
2	5	1700	0.6619395018	0.6957998872	-4.962350368
2	4	1700	0.6953213215	0.722394526	-5.26303339
2	3	1700	0.734241724	0.7147892118	-5.613604546
2	2	1700	0.7164703012	0.6597551107	-5.453530312
2	1	1700	1.176433444	0.9326151609	-9.596600533
2	5	1600	0.742086947	0.708702445	-5.684269905

2	4	1600	0.7196531296	0.7155632973	-5.482199669
2	3	1600	0.9031484723	0.7710622549	-7.135014534
2	2	1600	0.6748020053	0.6593470573	-5.07820797
2	1	1600	1.179988146	0.9471683502	-9.62861824
2	5	1500	0.7823683619	0.7509694099	-6.047100544
2	4	1500	0.6945719719	0.6774622798	-5.25628376
2	3	1500	0.9020032883	0.7921122313	-7.124699593
2	2	1500	0.809691608	0.727829814	-6.293211937
2	1	1500	1.216102123	0.9684473872	-9.953911781
2	5	1400	0.7896073461	0.7188962698	-6.112304688
2	4	1400	0.8348366022	0.7850786448	-6.519702435
2	3	1400	0.9211603403	0.8249393702	-7.297254562
2	2	1400	0.6387543678	0.6664654613	-4.753512383
2	1	1400	1.299665451	1.012672544	-10.70659924
2	5	1300	0.6048442125	0.6057736278	-4.448070526
2	4	1300	0.8689470291	0.7560015917	-6.826948643
2	3	1300	0.8993613124	0.7494543791	-7.100902557
2	2	1300	0.6637576818	0.6591304541	-4.978727341
2	1	1300	1.341477156	1.014546037	-11.08321381
2	5	1200	0.8737737536	0.8253135681	-6.870424747
2	4	1200	0.7119201422	0.7205659747	-5.412545681
2	3	1200	0.7887482643	0.7054790854	-6.104566574
2	2	1200	0.7829755545	0.7316241264	-6.052569389
2	1	1200	1.48260808	1.115146518	-12.35443497
2	5	1100	0.7144044042	0.6503556371	-5.434922218
2	4	1100	0.8725556135	0.7658529878	-6.859452724
2	3	1100	0.8309870958	0.7320563793	-6.485028267
2	2	1100	0.7048960328	0.7000400424	-5.349276543
2	1	1100	1.300002575	0.9930742383	-10.70963573
2	5	1000	0.6938481331	0.7037286162	-5.249763966
2	4	1000	0.6989380717	0.6886147261	-5.295610905
2	3	1000	0.4989725947	0.5827876925	-3.49444294
2	2	1000	0.7986606956	0.7331919074	-6.193851948
2	1	1000	1.927476525	1.251676321	-16.36154175
2	5	900	0.7138508558	0.7107282877	-5.429935932

2	4	900	0.6443992853	0.6490980387	-4.804358482
2	3	900	0.919637382	0.7856777906	-7.283536911
2	2	900	0.8344554901	0.7845714688	-6.516269684
2	1	900	1.848977089	1.222257137	-15.65446663
2	5	800	0.5231150389	0.7038199306	-3.711903572
2	4	800	0.6808794737	0.6628366113	-5.132950306
2	3	800	0.852167964	0.7796586752	-6.675813198
2	2	800	0.595733285	0.6330820322	-4.366004467
2	1	800	2.504965782	1.46028924	-21.56321526
2	5	700	0.7396426797	0.7161124945	-5.66225338
2	4	700	0.7701472044	0.7216474414	-5.937019825
2	3	700	0.77451092	0.706956625	-5.976325035
2	2	700	1.050639153	0.9141227007	-8.463521004
2	1	700	2.124006271	1.33160758	-18.13176155
2	5	600	0.6743027568	0.6632645726	-5.073710918
2	4	600	0.6699153781	0.6492835283	-5.034192085
2	3	600	0.7156595588	0.7340865135	-5.446228027
2	2	600	0.5846118927	0.6367141008	-4.26583004
2	1	600	2.900306463	1.479292274	-25.12420464
2	5	500	0.6513075829	0.655412674	-4.866584301
2	4	500	0.694020927	0.7148762941	-5.251320362
2	3	500	0.9399372935	0.7996410131	-7.466385841
2	2	500	0.8059207797	0.7640784383	-6.259246349
2	1	500	3.650374174	1.744267702	-31.88035965
2	5	400	0.64277637	0.5438946486	-4.789740562
2	4	400	0.3861892223	0.5158202648	-2.47855854
2	3	400	0.6203001738	0.6998038292	-4.58728838
2	2	400	0.8731620908	0.812648952	-6.864915371
2	1	400	4.107433319	1.900986671	-35.99727249
2	5	300	0.4888264239	0.5455750823	-3.40305233
2	4	300	0.7896912694	0.7312172651	-6.113060474
2	3	300	0.8384999037	0.7623553276	-6.552699566
2	2	300	1.031083465	0.9061968923	-8.28737545
2	1	300	5.113160133	2.093953609	-45.05625153
2	5	200	0.5557614565	0.645383656	-4.005962372

2	4	200	0.4964937568	0.5621075034	-3.47211504
2	3	200	0.5634170175	0.5984631777	-4.074919224
2	2	200	1.114837408	0.9335843921	-9.041779518
2	1	200	6.219011784	2.304397821	-55.01708984
2	5	120	0.5554152131	0.6408662796	-4.002843857
2	4	120	0.6663722992	0.6585810781	-5.002278328
2	3	120	0.6464918256	0.7194212675	-4.823206902
2	2	120	1.551237583	1.081834555	-12.97260857
2	1	120	6.66322422	2.336665154	-59.01828766
2	5	60	1.15962863	0.9203531146	-9.445232391
2	4	60	0.8308532834	0.8103474379	-6.483823299
2	3	60	1.65702343	1.180403709	-13.92546368
2	2	60	3.531594038	1.672044992	-30.81046104
2	1	60	7.676782131	2.505890131	-68.14780426
2	5	30	2.397196293	1.426580667	-20.59249115
2	4	30	5.7546525	2.249124289	-50.83442307
2	3	30	2.58392477	1.496732473	-22.27443123
2	2	30	6.602755547	2.340584278	-58.47362518
2	1	30	8.560560226	2.647785664	-76.10834503
3	25	2500	0.1110382527	0.2918872833	-0.0001653432846
3	24	2500	0.07300168276	0.2381259501	0.3424450755
3	25	2400	0.1110233516	0.2918872833	-3.11E-05
3	24	2400	0.1110557467	0.2918523252	-0.0003229379654
3	25	2300	0.09595496207	0.266274631	0.1356958151
3	24	2300	0.08019564301	0.2557900846	0.2776462436
3	25	2200	0.1110268384	0.2918840945	-6.25E-05
3	24	2200	0.1110232323	0.2918845713	-3.00E-05
3	25	2100	0.1075260192	0.2878101468	0.0314707756
3	24	2100	0.08381401002	0.2399135083	0.2450541854
3	25	2000	0.1110445857	0.2918866277	-0.000222325325
3	24	2000	0.1110759005	0.2918871343	-0.0005044937134

3	25	1900	0.111087583	0.2918496132	-0.0006096363068
3	24	1900	0.111089088	0.2918871939	-0.0006232261658
3	25	1800	0.06250821799	0.2261473238	0.4369637966
3	24	1800	0.1101392284	0.2902720571	0.007932543755
3	25	1700	0.1114432588	0.2918868661	-0.003813385963
3	24	1700	0.1112674698	0.2918866575	-0.00223004818
3	25	1600	0.05500031635	0.203306824	0.5045904517
3	24	1600	0.1108430177	0.2913514078	0.001593232155
3	25	1500	0.1110839397	0.2918829918	-0.0005768537521
3	24	1500	0.111385271	0.2918978333	-0.003291130066
3	25	1400	0.1110212356	0.2918872237	-1.20E-05
3	24	1400	0.1110211164	0.2918873429	-1.10E-05
3	25	1300	0.1113458648	0.2918850482	-0.002936124802
3	24	1300	0.1113186479	0.2918856144	-0.002690911293
3	25	1200	0.07276926935	0.2329270393	0.3445385098
3	24	1200	0.1112826914	0.2918864191	-0.002367138863
3	25	1100	0.1113367304	0.2918698788	-0.002853870392
3	24	1100	0.05665197223	0.2078977525	0.4897133112
3	25	1000	0.11105977	0.2918847501	-0.0003591775894
3	24	1000	0.0486862734	0.183457464	0.5614635348
3	25	900	0.05651999265	0.1950805187	0.4909021258
3	24	900	0.04190809652	0.1787942946	0.6225172281
3	25	800	0.1110537052	0.2918422222	-0.0003044605255
3	24	800	0.04292089492	0.1755179465	0.6133945584
3	25	700	0.05201608688	0.1994673312	0.5314705968

3	24	700	0.04833192378	0.1959900409	0.564655304
3	25	600	0.04611790925	0.1996122897	0.584597826
3	24	600	0.0473054871	0.1902321875	0.5739008188
3	25	500	0.07252944261	0.226685375	0.3466987014
3	24	500	0.04468350485	0.1824360341	0.5975180864
3	25	400	0.04628177732	0.1883271635	0.5831217766
3	24	400	0.05154321343	0.1805745661	0.5357300043
3	25	300	0.1159437448	0.3002142906	-0.04435098171
3	24	300	0.05426152423	0.1981235445	0.5112450719
3	25	200	0.0839087218	0.2754082084	0.2442010045
3	24	200	0.04662164301	0.1754648685	0.580060482
3	25	120	0.05821399763	0.1807801723	0.4756435752
3	24	120	0.0358527936	0.1543809921	0.6770597696
3	25	60	0.08398748934	0.2282078713	0.2434915304
3	24	60	0.06552296877	0.208540678	0.4098087549
3	25	30	0.06620152295	0.1872998178	0.4036967754
3	24	30	0.2411623448	0.428437084	-1.17224431
3	23	2500	0.05443180352	0.2019831687	0.5097112656
3	22	2500	0.1110232249	0.2918871343	-2.99E-05
3	21	2500	0.06899578124	0.2130881846	0.3785278201
3	23	2400	0.1110246629	0.2918877602	-4.29E-05
3	22	2400	0.1110270396	0.2918873727	-6.44E-05
3	21	2400	0.1110215634	0.2918871045	-1.50E-05
3	23	2300	0.1113186926	0.2918860316	-0.00269138813
3	22	2300	0.04068202525	0.1645784378	0.6335609555
3	21	2300	0.3025299907	0.4376181066	-1.725006819
3	23	2200	0.111021176	0.2918872237	-1.14E-05
3	22	2200	0.06061366946	0.2278077155	0.4540287852
3	21	2200	0.1154756919	0.2918872237	-0.04013502598
3	23	2100	0.1025380716	0.2791032791	0.07639914751
3	22	2100	0.1108679324	0.2916163802	0.001368820667
3	21	2100	0.1083619446	0.2868568003	0.02394121885

3	23	2000	0.08463609964	0.2489167154	0.237649262
3	22	2000	0.1060279608	0.2709414065	0.04496431351
3	21	2000	0.05518687516	0.2044599503	0.5029100776
3	23	1900	0.1111877933	0.2918872833	-0.001512289047
3	22	1900	0.1110881194	0.2918536663	-0.0006145238876
3	21	1900	0.04353223369	0.1877359897	0.6078879833
3	23	1800	0.06751362979	0.2052899897	0.3918780684
3	22	1800	0.07172883302	0.217252925	0.3539101481
3	21	1800	0.07256424427	0.2317136824	0.3463852406
3	23	1700	0.1121871844	0.2918539047	-0.01051425934
3	22	1700	0.06578522921	0.2220045626	0.4074465036
3	21	1700	0.07281722873	0.2322777808	0.3441064954
3	23	1600	0.05582977459	0.1978107989	0.4971191883
3	22	1600	0.07046494633	0.2276350558	0.3652944565
3	21	1600	0.07304501534	0.2362892181	0.3420547247
3	23	1500	0.06981664151	0.2268825471	0.3711339831
3	22	1500	0.1117800027	0.2918739915	-0.006846547127
3	21	1500	0.04568492621	0.1778642684	0.5884978771
3	23	1400	0.06079841778	0.2146923989	0.4523646832
3	22	1400	0.04811684042	0.1888540685	0.5665926337
3	21	1400	0.04804809764	0.1946288645	0.5672118664
3	23	1300	0.06806495041	0.2354326695	0.3869121075
3	22	1300	0.08525302261	0.2554613054	0.2320923805
3	21	1300	0.05731578916	0.1888568848	0.4837340713
3	23	1200	0.08389496803	0.2567102909	0.2443249226
3	22	1200	0.1111596078	0.2918873429	-0.00125849247
3	21	1200	0.04968544096	0.2010252476	0.5524636507
3	23	1100	0.0469353497	0.1874916553	0.5772348046
3	22	1100	0.06324283779	0.2284230292	0.430346787
3	21	1100	0.06048053503	0.2142212391	0.4552279711
3	23	1000	0.05474214628	0.2071470767	0.5069159269

3	22	1000	0.0523510389	0.1895022392	0.5284535289
3	21	1000	0.05353762582	0.2021921128	0.517765522
3	23	900	0.05185344815	0.1941392869	0.5329355001
3	22	900	0.06074625254	0.182550475	0.4528345466
3	21	900	0.04880306125	0.1890242994	0.5604115725
3	23	800	0.08258542418	0.250246942	0.2561205029
3	22	800	0.06339354813	0.2040976584	0.4289892912
3	21	800	0.06243650243	0.2088722289	0.4376097918
3	23	700	0.03871754929	0.1777040958	0.6512557268
3	22	700	0.06129962951	0.2120242119	0.4478500485
3	21	700	0.1104403287	0.2909490168	0.005220413208
3	23	600	0.06683681905	0.1957320273	0.3979744315
3	22	600	0.03332091495	0.157207638	0.6998653412
3	21	600	0.1610855162	0.3141857088	-0.4509607553
3	23	500	0.05039899424	0.1995307505	0.5460363626
3	22	500	0.05011189729	0.1992826015	0.5486223698
3	21	500	0.04183370993	0.1747279167	0.6231873035
3	23	400	0.04979862273	0.1727909148	0.5514441729
3	22	400	0.04100429267	0.1815917045	0.6306581497
3	21	400	0.04121331871	0.1728555262	0.6287753582
3	23	300	0.03624789044	0.1556498557	0.6735009551
3	22	300	0.0492012687	0.1754148006	0.5568248034
3	21	300	0.0446713753	0.1861724406	0.5976272821
3	23	200	0.05367431045	0.1921755373	0.5165343285
3	22	200	0.03655837104	0.1708960533	0.6707043648
3	21	200	0.1022881046	0.2782921791	0.07865071297
3	23	120	0.1187946051	0.2956215739	-0.07002985477
3	22	120	0.02679927647	0.1410974562	0.7586083412
3	21	120	0.1207528338	0.3081608713	-0.08766841888
3	23	60	0.1102626473	0.2827867866	0.006820857525
3	22	60	0.1741522551	0.337430954	-0.5686579943
3	21	60	0.08115431666	0.2501170039	0.2690110803

3	23	30	0.1175768226	0.2983873487	-0.059060812
3	22	30	0.1340676546	0.3240211904	-0.2076002359
3	21	30	0.2640343308	0.4254637659	-1.378261328
3	20	2500	0.04753629118	0.1994734257	0.5718218684
3	19	2500	0.111218892	0.2918861806	-0.001792430878
3	18	2500	0.03988436237	0.1691472977	0.6407458186
3	17	2500	0.05751085281	0.1878142357	0.4819770455
3	16	2500	0.04908584431	0.1885896176	0.5578644276
3	20	2400	0.1486866027	0.3197612762	-0.3392788172
3	19	2400	0.04478037357	0.16523242	0.5966454744
3	18	2400	0.1084758267	0.2885731757	0.02291542292
3	17	2400	0.06245431304	0.2331207246	0.4374493957
3	16	2400	0.05231880024	0.1981946528	0.5287439823
3	20	2300	0.05152356625	0.1785681695	0.5359069109
3	19	2300	4.655450821	1.978467226	-40.93348312
3	18	2300	0.09901340306	0.2823559344	0.1081472635
3	17	2300	0.04594376683	0.1807819903	0.5861663818
3	16	2300	0.03230805695	0.1645548791	0.7089885473
3	20	2200	0.1077119708	0.2847166061	0.02979582548
3	19	2200	0.1110620648	0.2918840349	-0.0003798007965
3	18	2200	0.05657891557	0.2194543332	0.4903714061
3	17	2200	0.05880295113	0.1937700212	0.4703386426
3	16	2200	0.04579181969	0.2000173628	0.5875350237
3	20	2100	0.0583326444	0.2003731281	0.4745748639
3	19	2100	0.1157133132	0.2918868959	-0.04227542877
3	18	2100	0.05031643435	0.1895435303	0.5467799902
3	17	2100	0.05942158774	0.2167490423	0.4647663236
3	16	2100	0.06948943436	0.2107514441	0.374081254
3	20	2000	0.06044980884	0.2165994197	0.4555047154
3	19	2000	0.08649537712	0.2520065904	0.2209020257
3	18	2000	0.03538488597	0.1456139535	0.6812744141
3	17	2000	0.06319700181	0.2258064747	0.430759728

3	16	2000	0.2339546233	0.4391481876	-1.107321501
3	20	1900	0.05495425314	0.205054611	0.5050053596
3	19	1900	0.08920846134	0.2612292767	0.1964642406
3	18	1900	0.04420440644	0.1883094311	0.6018334627
3	17	1900	0.109292604	0.2894178331	0.01555842161
3	16	1900	0.06184327602	0.2122320682	0.442953229
3	20	1800	0.07751138508	0.2327284366	0.3018243313
3	19	1800	0.1113994941	0.2917886376	-0.003419160843
3	18	1800	0.1399719864	0.313177824	-0.2607828379
3	17	1800	0.05553389341	0.1876618862	0.4997843504
3	16	1800	0.03966777027	0.1559967101	0.6426967382
3	20	1700	0.09415221959	0.2354039252	0.1519338489
3	19	1700	0.04756863415	0.185510397	0.5715305805
3	18	1700	0.0482441932	0.1932933778	0.5654455423
3	17	1700	0.04473960027	0.1897227317	0.5970127583
3	16	1700	0.07215166092	0.2301855534	0.3501015306
3	20	1600	0.06169147044	0.2116172314	0.4443206191
3	19	1600	0.06530065835	0.2054815292	0.4118112326
3	18	1600	0.08078410476	0.2299974859	0.2723457217
3	17	1600	0.05201371759	0.1928555518	0.5314918756
3	16	1600	0.04261155054	0.173306182	0.6161809564
3	20	1500	0.05386957526	0.2024158984	0.5147755146
3	19	1500	0.1232805848	0.2910924852	-0.1104367971
3	18	1500	0.06056171656	0.2037340701	0.4544967413
3	17	1500	0.05474687368	0.1905516088	0.5068733692
3	16	1500	0.03439021483	0.1531648189	0.690233767
3	20	1400	0.1193075627	0.2918501496	-0.07465028763
3	19	1400	0.05533904955	0.2099437267	0.5015393496
3	18	1400	0.05971334502	0.1963789016	0.4621383548
3	17	1400	0.05155721307	0.2045080662	0.5356038809
3	16	1400	0.05269973353	0.1974882185	0.5253127217
3	20	1300	0.05266447738	0.1994238347	0.5256302953
3	19	1300	0.06012567133	0.2203466892	0.4584243298

3	18	1300	0.0628413409	0.2175791264	0.4339632392
3	17	1300	0.04701124877	0.19115448	0.576551199
3	16	1300	0.08639623225	0.2514062524	0.2217950821
3	20	1200	0.05682433397	0.1934872121	0.4881608486
3	19	1200	0.0533760488	0.1924649775	0.5192208886
3	18	1200	0.05932115763	0.2079523057	0.4656709433
3	17	1200	0.05517860502	0.204004243	0.5029845238
3	16	1200	0.05630999804	0.2072762549	0.4927936196
3	20	1100	0.05521078035	0.1969271153	0.502694726
3	19	1100	0.0493183285	0.1981327087	0.5557703972
3	18	1100	0.063618958	0.1984113157	0.4269589782
3	17	1100	0.05609704927	0.1975209266	0.4947117567
3	16	1100	0.04672566056	0.1815376282	0.5791235566
3	20	1000	0.04475651681	0.1882243603	0.5968604088
3	19	1000	0.07629434764	0.2327723056	0.3127866983
3	18	1000	0.06085272878	0.1953040063	0.4518754482
3	17	1000	0.04074807093	0.1642204821	0.6329660416
3	16	1000	0.02463254146	0.1254770309	0.7781249881
3	20	900	0.04380777851	0.1843322217	0.6054060459
3	19	900	0.08176730573	0.2232949734	0.2634896636
3	18	900	0.1873726845	0.4041230083	-0.6877396107
3	17	900	0.04527952522	0.1886460334	0.5921494961
3	16	900	0.09300187975	0.2858273089	0.1622954011
3	20	800	0.04342550784	0.1503044665	0.6088493466
3	19	800	0.046679575	0.1773989201	0.579538703
3	18	800	0.4282484949	0.6103364825	-2.85740304
3	17	800	0.02896596491	0.1432323903	0.7390921116
3	16	800	0.03015451133	0.1317990273	0.7283864021
3	20	700	0.04659727216	0.1950076073	0.5802800059
3	19	700	0.05420447513	0.1809243709	0.5117589235
3	18	700	0.04759217799	0.1892307252	0.5713185072
3	17	700	0.0309651345	0.1405880451	0.7210848331
3	16	700	0.04063538462	0.1667532921	0.6339810491
3	20	600	0.03692895919	0.1723003387	0.6673663259
3	19	600	0.05792163685	0.2009081393	0.478276968

3	18	600	0.0363685675	0.1723492593	0.6724139452
3	17	600	0.04180837423	0.1489436626	0.6234154701
3	16	600	0.02559508383	0.1306398362	0.7694549561
3	20	500	0.03702519834	0.1430658847	0.6664994359
3	19	500	0.04591336846	0.1680854857	0.5864402056
3	18	500	0.04420785606	0.1616846025	0.6018024087
3	17	500	0.1338980943	0.3434455991	-0.2060729265
3	16	500	0.03649710864	0.1687349379	0.6712561846
3	20	400	0.03826542199	0.1214228645	0.6553282738
3	19	400	0.03778574616	0.1609116048	0.6596488953
3	18	400	0.04510162398	0.175848335	0.5937519073
3	17	400	0.2791931033	0.4625441432	-1.514802456
3	16	400	0.03047797643	0.1414357722	0.7254728079
3	20	300	0.0338876918	0.1257009059	0.6947602034
3	19	300	0.08286403865	0.2351223975	0.253610909
3	18	300	0.1149648875	0.3154240549	-0.03553414345
3	17	300	0.08832412958	0.2511810362	0.2044297457
3	16	300	0.1097064465	0.2824031115	0.01183074713
3	20	200	0.07137736678	0.2103999108	0.3570759296
3	19	200	0.05545860529	0.1899425536	0.5004624724
3	18	200	0.1426755637	0.3331176937	-0.28513515
3	17	200	0.1009511128	0.2763992846	0.09069353342
3	16	200	0.1674636155	0.3543860912	-0.5084108114
3	20	120	0.05236627907	0.1946620941	0.5283162594
3	19	120	0.1836065501	0.3546386361	-0.6538165808
3	18	120	0.07093842328	0.2275736332	0.3610296845
3	17	120	0.09798577428	0.2467224598	0.1174035072
3	16	120	0.09012928605	0.2611634731	0.1881699562
3	20	60	0.1439733952	0.3044655919	-0.2968251705
3	19	60	0.04500363022	0.1738341749	0.5946345329
3	18	60	0.2695135176	0.4289689064	-1.42761445
3	17	60	0.1000528336	0.2589528263	0.09878468513
3	16	60	0.126989603	0.3049210012	-0.143845439
3	20	30	0.2930910289	0.4608353078	-1.639986515

3	19	30	0.09511777014	0.2566763461	0.1432366967
3	18	30	0.4195323586	0.5758959055	-2.778893232
3	17	30	0.4924069941	0.6666586995	-3.435304165
3	16	30	0.4532284141	0.6088117361	-3.082406998
3	15	2500	0.03449232876	0.1683033407	0.6893140078
3	14	2500	0.5305898786	0.6793702245	-3.779232025
3	13	2500	0.04293306172	0.1694807112	0.6132849455
3	12	2500	0.02785985731	0.1358167678	0.7490552664
3	11	2500	0.05114040524	0.2078501284	0.5393582582
3	15	2400	0.05090480298	0.2009512931	0.5414803624
3	14	2400	0.0518944785	0.2044355869	0.5325659513
3	13	2400	0.02481614053	0.1253556311	0.7764712572
3	12	2400	0.03644336388	0.1496064663	0.6717402339
3	11	2400	0.02973521687	0.1380278617	0.7321631908
3	15	2300	0.06500837207	0.2170573771	0.4144439697
3	14	2300	0.05089917034	0.1958883256	0.541531086
3	13	2300	0.06697358191	0.2393231839	0.3967425227
3	12	2300	0.05322803184	0.2016489059	0.5205541253
3	11	2300	0.05476243049	0.1787551939	0.5067331791
3	15	2200	0.04670553654	0.1750633717	0.5793048143
3	14	2200	0.2215168774	0.4205760956	-0.9952898026
3	13	2200	0.04306690767	0.184486717	0.6120793819
3	12	2200	0.05401661247	0.2124350965	0.5134510994
3	11	2200	0.03736256808	0.1556387395	0.6634606123
3	15	2100	0.04782372713	0.1804511547	0.5692328215
3	14	2100	0.04437293485	0.1835182607	0.6003154516
3	13	2100	0.05359141156	0.1868364811	0.5172810555
3	12	2100	0.2830391526	0.4630457759	-1.549445152
3	11	2100	0.1095970869	0.3012762666	0.01281583309
3	15	2000	0.0541575551	0.2070982903	0.5121815205
3	14	2000	0.04862220958	0.1995380372	0.5620405674
3	13	2000	0.03028875031	0.1575053185	0.7271772623
3	12	2000	0.4383288324	0.589648068	-2.948200703
3	11	2000	0.257876277	0.4564041495	-1.322793245
3	15	1900	0.0378934592	0.17309089	0.6586786509

3	14	1900	0.04167614132	0.169300124	0.6246066093
3	13	1900	0.04674950987	0.197296381	0.5789087415
3	12	1900	0.1654098332	0.3813554049	-0.4899115562
3	11	1900	0.0271739848	0.1235352978	0.7552332282
3	15	1800	0.04867019877	0.1791998893	0.5616083145
3	14	1800	0.02410347573	0.1254496127	0.7828904986
3	13	1800	0.03473573178	0.1563084126	0.6871215701
3	12	1800	0.3800812662	0.5446534753	-2.423542023
3	11	1800	0.5505331755	0.6425080299	-3.958869457
3	15	1700	0.05182235688	0.2045535147	0.5332155824
3	14	1700	0.03415963799	0.1564487964	0.6923106909
3	13	1700	0.03522241861	0.1643279493	0.6827377677
3	12	1700	0.03183155507	0.1416378468	0.7132806778
3	11	1700	0.05535251647	0.2221029699	0.5014180541
3	15	1600	0.05199846625	0.199185133	0.531629324
3	14	1600	0.03886052966	0.1633281708	0.6499679089
3	13	1600	0.1240392774	0.3246387541	-0.1172707081
3	12	1600	0.02623773552	0.1348438263	0.7636663914
3	11	1600	0.03778985888	0.1647861451	0.6596118212
3	15	1500	0.03034484014	0.1448692828	0.7266720533
3	14	1500	0.04140887409	0.1684571803	0.6270139217
3	13	1500	0.3667796552	0.4516468942	-2.303729057
3	12	1500	0.1647309959	0.3559846282	-0.4837969542
3	11	1500	0.069792144	0.2256258428	0.3713546395
3	15	1400	0.07502068579	0.2538440228	0.3242591023
3	14	1400	0.03810875118	0.1587851495	0.6567394733
3	13	1400	0.1718847603	0.393053472	-0.5482338667
3	12	1400	0.369235605	0.5196831226	-2.325850725
3	11	1400	0.3276097775	0.5159087181	-1.950910568
3	15	1300	0.05016740412	0.1905467957	0.548122406
3	14	1300	0.02964619361	0.1508479565	0.7329650521
3	13	1300	0.03826605156	0.1643777341	0.6553225517
3	12	1300	0.03684290498	0.1541282684	0.6681414247
3	11	1300	0.04933867604	0.2006001025	0.5555870533
3	15	1200	0.03699834645	0.1468269378	0.6667413116

3	14	1200	0.0476429984	0.1709887534	0.5708607435
3	13	1200	0.05161875486	0.186020568	0.5350495577
3	12	1200	0.1405670345	0.3418945968	-0.2661427259
3	11	1200	0.03948055953	0.1603856981	0.6443830729
3	15	1100	0.05811660364	0.2210304737	0.4765208364
3	14	1100	0.04030849785	0.1533771455	0.6369254589
3	13	1100	0.03854458407	0.1690615714	0.6528137326
3	12	1100	0.06974302232	0.2473153174	0.3717970848
3	11	1100	0.4312228262	0.5901277661	-2.884193897
3	15	1000	0.4142142832	0.5738848448	-2.730991364
3	14	1000	0.08841583133	0.214463383	0.2036037445
3	13	1000	0.03988670185	0.1628089845	0.6407247782
3	12	1000	0.5496519804	0.6366839409	-3.950932503
3	11	1000	0.1146163717	0.3015231192	-0.03239488602
3	15	900	0.04059938714	0.1568803787	0.6343052983
3	14	900	0.3432848155	0.5170342326	-2.092101812
3	13	900	0.1363262385	0.3417560458	-0.2279442549
3	12	900	0.1843441427	0.3934841752	-0.6604603529
3	11	900	0.1225906163	0.3335076272	-0.1042220592
3	15	800	0.03806037456	0.1605117321	0.6571751833
3	14	800	0.04431388527	0.1594256461	0.6008473635
3	13	800	0.1198100224	0.3140402436	-0.07917606831
3	12	800	0.0995054394	0.2983443737	0.1037153006
3	11	800	0.2384546697	0.4088555872	-1.147855282
3	15	700	0.03844466433	0.1699728966	0.6537137032
3	14	700	0.05665751174	0.2016839534	0.4896634817
3	13	700	0.08181859553	0.2522819638	0.263027668
3	12	700	0.5501095653	0.6370271444	-3.955053806
3	11	700	0.1055861488	0.3060652316	0.04894393682
3	15	600	0.0490523912	0.1904319227	0.5581657887
3	14	600	0.3929145932	0.5549289584	-2.539136648
3	13	600	0.3651163578	0.4812986255	-2.288747072
3	12	600	0.4445640147	0.5865249038	-3.004363537

3	11	600	0.6268642545	0.6670689583	-4.646413326
3	15	500	0.2654836774	0.4199748039	-1.391316175
3	14	500	0.09921765327	0.2822048664	0.106307447
3	13	500	0.261012882	0.438377142	-1.351046085
3	12	500	0.2938164175	0.459427774	-1.646520376
3	11	500	0.2068570405	0.4159549773	-0.8632428646
3	15	400	0.05586650968	0.2060305178	0.4967883229
3	14	400	0.1114536673	0.2999167144	-0.003907084465
3	13	400	0.2517357767	0.4366551042	-1.267483473
3	12	400	0.180265516	0.3659541011	-0.6237226725
3	11	400	0.256251514	0.4362451434	-1.308158398
3	15	300	0.1891047508	0.3704682589	-0.7033410072
3	14	300	0.2592539191	0.4259130359	-1.335202217
3	13	300	0.1215537563	0.3021413386	-0.09488260746
3	12	300	0.386895299	0.5086159706	-2.484918594
3	11	300	0.3477756381	0.5007630587	-2.132552385
3	15	200	0.2594571114	0.4558495581	-1.337032557
3	14	200	0.3022454381	0.4776443541	-1.722444057
3	13	200	0.2573117614	0.4261779785	-1.317708731
3	12	200	0.2118489742	0.4131186903	-0.9082072973
3	11	200	0.3163094223	0.4906403422	-1.849123716
3	15	120	0.2368592769	0.4365634024	-1.13348484
3	14	120	0.1875563562	0.3684366047	-0.6893939972
3	13	120	0.3406505883	0.455745697	-2.068374157
3	12	120	0.4587278366	0.6166249514	-3.131942749
3	11	120	0.2390273809	0.443652153	-1.153013945
3	15	60	0.2120733708	0.3943722248	-0.9102284908
3	14	60	0.3017348349	0.4624264836	-1.717844486
3	13	60	0.2374329567	0.3600088358	-1.138652325
3	12	60	0.4286468625	0.5720809698	-2.86099124
3	11	60	0.1993176192	0.4090894163	-0.7953324318
3	15	30	0.4681873918	0.5342746973	-3.217148304
3	14	30	0.6358308792	0.7134127617	-4.727179527

3	13	30	0.1766868979	0.3653252721	-0.5914885998
3	12	30	0.5039370656	0.6346769929	-3.539159775
3	11	30	0.2541417181	0.4298163056	-1.28915453
3	10	2500	0.2097087204	0.3862343132	-0.8889291286
3	9	2500	0.05844430998	0.2030024976	0.4735690355
3	8	2500	0.8354798555	0.7636087537	-6.525496483
3	7	2500	0.7543514967	0.7527862787	-5.794741154
3	6	2500	0.8665383458	0.7559987903	-6.805252552
3	10	2400	0.1498300284	0.3248079419	-0.3495781422
3	9	2400	0.4163327217	0.5800817609	-2.750072956
3	8	2400	0.818025887	0.7240356803	-6.368281841
3	7	2400	1.138143182	0.9101888537	-9.251704216
3	6	2400	0.8219183087	0.7793388963	-6.403342247
3	10	2300	0.03481064364	0.1533106267	0.6864467859
3	9	2300	0.8669617772	0.8427128792	-6.809066296
3	8	2300	0.3431138098	0.4876235127	-2.09056139
3	7	2300	0.4227895737	0.5365983248	-2.808232546
3	6	2300	0.8355385065	0.797612071	-6.526024818
3	10	2200	0.14770028	0.3668037057	-0.3303947449
3	9	2200	0.1753040254	0.3710781932	-0.5790325403
3	8	2200	0.6762173772	0.6784995198	-5.090956688
3	7	2200	0.6281622052	0.676936388	-4.65810442
3	6	2200	0.929130435	0.8026012182	-7.369044304
3	10	2100	0.4990566373	0.6140869856	-3.49519968
3	9	2100	0.3932485282	0.5149924755	-2.542144537
3	8	2100	0.537997365	0.6246198416	-3.845954418
3	7	2100	0.5026847124	0.6138242483	-3.527879238
3	6	2100	0.8268706203	0.7522708178	-6.447949886
3	10	2000	0.5550380349	0.6825636029	-3.999446392
3	9	2000	0.7049074173	0.7327398062	-5.349379063
3	8	2000	0.38738361	0.5217754245	-2.48931694
3	7	2000	0.5926531553	0.6322182417	-4.338261127
3	6	2000	1.036104441	0.8774614334	-8.332600594
3	10	1900	0.04220519587	0.1811280698	0.6198411584
3	9	1900	0.5170047283	0.5654504895	-3.656865597

3	8	1900	0.351529628	0.4930514395	-2.166365862
3	7	1900	0.343282938	0.4840732515	-2.092084885
3	6	1900	0.6808441877	0.6490339041	-5.132632256
3	10	1800	0.5826686621	0.6652897596	-4.248326778
3	9	1800	0.4523905814	0.6053931117	-3.074860096
3	8	1800	0.3526598215	0.509804368	-2.176546097
3	7	1800	0.4733554721	0.5645102262	-3.263699532
3	6	1800	0.7813290358	0.7303658128	-6.037738323
3	10	1700	0.4942711294	0.6165920496	-3.452095032
3	9	1700	0.1196311116	0.3135392666	-0.0775645971<br>3
3	8	1700	0.505848825	0.6484425068	-3.556379795
3	7	1700	0.6512054801	0.6587975621	-4.865664482
3	6	1700	0.5322008729	0.6514356732	-3.793743134
3	10	1600	0.286850214	0.4722332358	-1.583772898
3	9	1600	0.4818188548	0.5979391932	-3.339932442
3	8	1600	0.1546864361	0.3529633582	-0.3933217525
3	7	1600	0.4966211319	0.5797327757	-3.47326231
3	6	1600	0.591260016	0.6347306371	-4.325712204
3	10	1500	0.1771826446	0.3914192617	-0.5959540606
3	9	1500	0.8730872273	0.739599824	-6.864240646
3	8	1500	0.7438924313	0.7124683261	-5.700532436
3	7	1500	0.40941149	0.5769483447	-2.687730551
3	6	1500	0.8165995479	0.7884837985	-6.355434418
3	10	1400	0.3802132607	0.5377190113	-2.424730778
3	9	1400	0.3742284179	0.512694478	-2.370823145
3	8	1400	0.2577982843	0.4320706427	-1.322090864
3	7	1400	0.4916363657	0.6261675358	-3.428362846
3	6	1400	0.5531224012	0.6097181439	-3.982192039
3	10	1300	0.1811620295	0.4027108252	-0.6317977905
3	9	1300	0.1432014108	0.3536869884	-0.2898715734
3	8	1300	0.133097142	0.3249369562	-0.1988584995
3	7	1300	0.3435718417	0.4731320441	-2.094687223
3	6	1300	0.5894922018	0.6530867219	-4.309789181
3	10	1200	0.2430012524	0.443028748	-1.188807964

3	9	1200	0.1508417726	0.3186141849	-0.3586913347
3	8	1200	0.3343106806	0.4779129028	-2.011268139
3	7	1200	0.481770426	0.5890320539	-3.339496136
3	6	1200	0.6763288379	0.6774309874	-5.091960907
3	10	1100	0.09438646585	0.2968827188	0.149823904
3	9	1100	0.2331772149	0.4410339892	-1.100319147
3	8	1100	0.3056337833	0.4370369911	-1.75296402
3	7	1100	0.6264497638	0.653650403	-4.642680168
3	6	1100	0.7475384474	0.7560397387	-5.733373165
3	10	1000	0.5584397316	0.6450432539	-4.030086994
3	9	1000	0.1802157611	0.3540643752	-0.6232744455
3	8	1000	0.4146169126	0.5349257588	-2.734618187
3	7	1000	0.5071380734	0.5875257254	-3.567992687
3	6	1000	0.6778675914	0.6700912118	-5.105820656
3	10	900	0.05902297422	0.2213349789	0.4683567882
3	9	900	0.3084947765	0.4597342908	-1.778734207
3	8	900	0.4360812306	0.5151679516	-2.927955627
3	7	900	0.6759734154	0.6761571765	-5.088758945
3	6	900	0.5192183256	0.6125635505	-3.676804543
3	10	800	0.2810867429	0.4697312415	-1.531858921
3	9	800	0.2751958966	0.4586210847	-1.478798151
3	8	800	0.4782060981	0.6523936987	-3.307391167
3	7	800	0.6614031792	0.6777698398	-4.957519054
3	6	800	0.4760150909	0.5842723846	-3.28765583
3	10	700	0.4264668524	0.5354395509	-2.841355085
3	9	700	0.2019670308	0.3898088336	-0.819196701
3	8	700	0.4847242236	0.5993540883	-3.366102219
3	7	700	0.628860116	0.6304098964	-4.664391518
3	6	700	0.5929428935	0.6398125887	-4.34087038
3	10	600	0.5063812733	0.5858706236	-3.561175823
3	9	600	0.7477040887	0.703347981	-5.734865665
3	8	600	0.5955681801	0.6433649063	-4.364517689
3	7	600	0.494422853	0.6013911963	-3.453461647
3	6	600	0.7061172724	0.673404038	-5.360277176
3	10	500	0.3413658142	0.4867252707	-2.074816465

3	9	500	0.4841360152	0.5887368917	-3.360804081
3	8	500	0.5427834392	0.6083149314	-3.889064789
3	7	500	0.5479136109	0.6579679847	-3.935273647
3	6	500	0.6031028628	0.6735185981	-4.432385445
3	10	400	0.3195758462	0.4985817075	-1.878545761
3	9	400	0.3609471619	0.5011153221	-2.251193285
3	8	400	0.4963008761	0.6087295413	-3.470377922
3	7	400	0.5173900723	0.590346694	-3.660336494
3	6	400	0.7639085054	0.7305598855	-5.88082552
3	10	300	0.6040124893	0.6419479251	-4.440578938
3	9	300	0.4315962791	0.5810745358	-2.887557983
3	8	300	0.5913573503	0.6512562037	-4.326588631
3	7	300	0.532292366	0.5784538388	-3.794567108
3	6	300	0.5731955767	0.6347733736	-4.162998199
3	10	200	0.2893373668	0.4595284462	-1.6061759
3	9	200	0.4187418818	0.57741642	-2.771773338
3	8	200	0.5477715731	0.6219421625	-3.93399477
3	7	200	0.5216618776	0.6464799047	-3.698813915
3	6	200	0.5132824779	0.5868526101	-3.623337746
3	10	120	0.2524545193	0.4370770454	-1.273957491
3	9	120	0.3138757348	0.5340493917	-1.82720232
3	8	120	0.4763323665	0.5802372694	-3.290513515
3	7	120	0.436114639	0.5582095385	-2.928256512
3	6	120	0.5403326154	0.6450304985	-3.866988659
3	10	60	0.4591819346	0.5628284216	-3.136032581
3	9	60	0.2757654786	0.4334977567	-1.483928204
3	8	60	0.3627587855	0.5376432538	-2.267511368
3	7	60	0.331609726	0.458199203	-1.986939669
3	6	60	0.7024440765	0.679250896	-5.327190876
3	10	30	0.6121053696	0.6347699165	-4.513474464
3	9	30	0.3868932724	0.4541291595	-2.484900236
3	8	30	0.5609437823	0.6102092862	-4.052641869
3	7	30	0.523182869	0.6504212618	-3.7125144
3	6	30	0.8685010672	0.7935140133	-6.822931767
3	5	2500	0.7017585039	0.6841837764	-5.321015835

3	4	2500	0.8863674402	0.7611395717	-6.98386097
3	3	2500	0.8701300621	0.7668827772	-6.837604523
3	2	2500	0.9389898181	0.8096339107	-7.45785141
3	1	2500	0.810915947	0.7856298089	-6.30423975
3	5	2400	0.6903356314	0.7002407312	-5.218124866
3	4	2400	0.4252694249	0.5689550638	-2.830569267
3	3	2400	1.029397845	0.8637644053	-8.272192955
3	2	2400	0.6642119288	0.6558694243	-4.98281908
3	1	2400	0.7682515979	0.7474088669	-5.91994524
3	5	2300	0.6365194321	0.6394938231	-4.733381748
3	4	2300	0.9539063573	0.8092938662	-7.59221077
3	3	2300	0.5556106567	0.6304977536	-4.00460434
3	2	2300	0.9622338414	0.7715739012	-7.667219162
3	1	2300	0.9331838489	0.8348083496	-7.405554771
3	5	2200	0.6996846199	0.6722580194	-5.302335262
3	4	2200	1.017246962	0.8192921877	-8.162744522
3	3	2200	0.8923276067	0.7584578991	-7.037546158
3	2	2200	0.7876002192	0.7018071413	-6.094225883
3	1	2200	0.7957192659	0.7559363246	-6.167356968
3	5	2100	0.6670915484	0.7344101071	-5.008756638
3	4	2100	0.9059721828	0.7904731631	-7.160449028
3	3	2100	0.8475425839	0.7627186775	-6.634150028
3	2	2100	0.9090286493	0.783133626	-7.187979698
3	1	2100	0.9842048883	0.8490278125	-7.865120888
3	5	2000	0.9367431402	0.8217192888	-7.437615395
3	4	2000	0.6992141008	0.7133516073	-5.298097134
3	3	2000	0.7790306807	0.7279938459	-6.017036915
3	2	2000	0.730271101	0.7004202604	-5.577839851
3	1	2000	0.970887363	0.8569904566	-7.745165825
3	5	1900	0.8084160089	0.7404413223	-6.281721592
3	4	1900	0.6715849638	0.7237626314	-5.049231052
3	3	1900	0.7441237569	0.7300502658	-5.702616215
3	2	1900	0.7047249675	0.6858155727	-5.347735882
3	1	1900	0.9912864566	0.872115612	-7.928908348
3	5	1800	0.6823113561	0.6970853806	-5.145847321

3	4	1800	0.951169014	0.8418713808	-7.567554474
3	3	1800	0.9670904875	0.8348101377	-7.710965157
3	2	1800	0.5742152333	0.6680361032	-4.172183037
3	1	1800	1.200070262	0.9859806895	-9.809506416
3	5	1700	0.5719083548	0.6361756325	-4.151404381
3	4	1700	0.9170091748	0.7804607153	-7.2598629
3	3	1700	0.71913445	0.7010514736	-5.477527618
3	2	1700	0.7674911022	0.6685612798	-5.913094997
3	1	1700	0.9707929492	0.8605880737	-7.744315147
3	5	1600	0.6660881042	0.6716614962	-4.999718189
3	4	1600	0.8472118378	0.7816095352	-6.631171227
3	3	1600	0.9330692291	0.7925604582	-7.404522896
3	2	1600	0.7246664762	0.6834049225	-5.527357101
3	1	1600	1.050356507	0.8735157847	-8.460974693
3	5	1500	0.6913723946	0.7053711414	-5.227463722
3	4	1500	1.074560881	0.8411538005	-8.678994179
3	3	1500	0.8416944742	0.7462593913	-6.581473827
3	2	1500	0.821156323	0.7351629138	-6.396478653
3	1	1500	1.324714661	1.007921457	-10.93222713
3	5	1400	0.8841602206	0.8025819659	-6.963979721
3	4	1400	0.7038133144	0.6638501883	-5.339524269
3	3	1400	0.862303257	0.7737959027	-6.767105579
3	2	1400	0.7572025657	0.7300992012	-5.820422173
3	1	1400	1.327551126	1.019764304	-10.95777607
3	5	1300	0.9326297045	0.7807826996	-7.40056324
3	4	1300	0.9039767385	0.7796044946	-7.142475128
3	3	1300	0.9536536932	0.8110935092	-7.589935303
3	2	1300	0.8459534645	0.7567974329	-6.61983633
3	1	1300	1.354884148	1.030107141	-11.20397568
3	5	1200	0.7097711563	0.7191253901	-5.393188953
3	4	1200	0.5874361992	0.6293762922	-4.291269302
3	3	1200	0.7823443413	0.7067573667	-6.04688406
3	2	1200	0.5819029808	0.6169977784	-4.241429806
3	1	1200	1.687054873	1.17530787	-14.19596767
3	5	1100	0.9613142014	0.8127000928	-7.658936501

3	4	1100	0.7566622496	0.7382210493	-5.815555096
3	3	1100	0.9002227783	0.7911547422	-7.108661652
3	2	1100	0.7025947571	0.6893430948	-5.328547955
3	1	1100	1.608948469	1.145201564	-13.49243355
3	5	1000	0.8132913709	0.7618461847	-6.32563591
3	4	1000	0.8373368382	0.7405732274	-6.542222977
3	3	1000	0.8650194407	0.7924509645	-6.79157114
3	2	1000	0.7076436281	0.7079885006	-5.374025345
3	1	1000	2.096152782	1.303491235	-17.88087654
3	5	900	0.5132721066	0.6116210222	-3.623244286
3	4	900	0.626365304	0.6515914798	-4.641919136
3	3	900	0.7396469712	0.6857860684	-5.662292004
3	2	900	0.734768033	0.7273811102	-5.618345261
3	1	900	1.990099549	1.272477508	-16.9256115
3	5	800	0.7857322097	0.7249677181	-6.077400208
3	4	800	0.7122853994	0.6731104255	-5.415835381
3	3	800	0.6727029085	0.6961295009	-5.059300423
3	2	800	0.7870804667	0.7586711645	-6.089544296
3	1	800	2.129262924	1.341437459	-18.17911148
3	5	700	0.5959554911	0.6320098639	-4.368006229
3	4	700	0.6267901659	0.6456096172	-4.645746231
3	3	700	0.9714046717	0.8175237775	-7.749824524
3	2	700	0.9761068225	0.8284932971	-7.792179108
3	1	700	2.834783554	1.561148643	-24.53401375
3	5	600	0.6616383195	0.7022896409	-4.959637165
3	4	600	0.6879249811	0.6727839708	-5.19641161
3	3	600	0.6746448278	0.6673018932	-5.07679224
3	2	600	0.8763666153	0.8033110499	-6.893779755
3	1	600	3.113946438	1.586040854	-27.04854393
3	5	500	0.62010324	0.6197333336	-4.585514545
3	4	500	0.7446274757	0.7491189241	-5.70715332
3	3	500	0.7359020114	0.720949173	-5.628559589
3	2	500	0.7715210915	0.7407621145	-5.949394703
3	1	500	3.934187412	1.86346209	-34.43677521
3	5	400	0.9089044333	0.8134518862	-7.186861038

3	4	400	0.6616584063	0.624191761	-4.959817886
3	3	400	0.8756169081	0.8348014951	-6.887027264
3	2	400	0.8211296201	0.7645658851	-6.396238327
3	1	400	4.486609459	1.982925057	-39.41265869
3	5	300	0.5709542036	0.6241029501	-4.142809391
3	4	300	0.667273283	0.6913231015	-5.01039362
3	3	300	0.6759803295	0.6788933873	-5.088821411
3	2	300	0.7749000788	0.7719660997	-5.979830265
3	1	300	4.634092331	2.007420778	-40.7410965
3	5	200	0.563206315	0.5998520255	-4.073021412
3	4	200	0.8771208525	0.8309978247	-6.90057373
3	3	200	0.9138337374	0.7818589211	-7.2312603
3	2	200	1.166545987	0.8873117566	-9.507539749
3	1	200	5.817421436	2.182422161	-51.39980698
3	5	120	0.6863476038	0.7483873963	-5.18220377
3	4	120	0.441547215	0.5778015852	-2.977189779
3	3	120	0.6405943632	0.6770886779	-4.770086288
3	2	120	1.898901701	1.185647607	-16.10415649
3	1	120	7.458183289	2.457243204	-66.17879486
3	5	60	0.5389319062	0.6164159775	-3.854372025
3	4	60	1.125766993	0.8793735504	-9.140227318
3	3	60	1.668983102	1.183101058	-14.03318882
3	2	60	3.693370819	1.691582918	-32.26764679
3	1	60	8.83296299	2.673862696	-78.5619812
3	5	30	1.51333642	1.054230809	-12.63121796
3	4	30	4.003577232	1.862878084	-35.0617981
3	3	30	4.404891014	1.87431109	-38.67658997
3	2	30	6.213890553	2.300964355	-54.97096252
3	1	30	8.533503532	2.670676231	-75.86463165
