# Supplementary figures and images for: Non-lethal imaging and modeling approaches for estimating dry mass in aquatic larvae
Source: PLoS One. 2026 Apr 17;21(4):e0345767. doi: 10.1371/journal.pone.0345767 (PMC13089712; doi:10.1371/journal.pone.0345767)

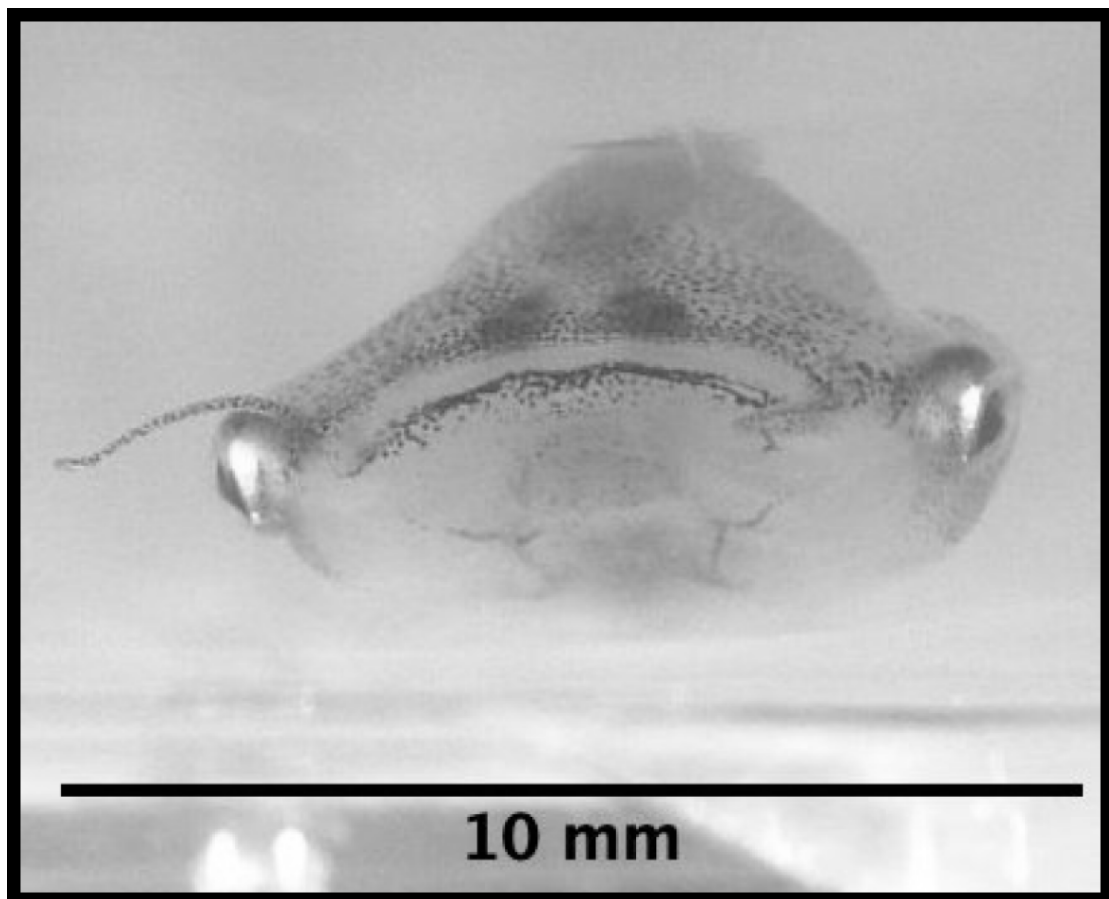

Supplement: S1 Fig — The only measurements obtained from the frontal view were the body width (longest distance from eye to eye) and body area. (PDF) [file pone.0345767.s004.pdf]
